# Unidirectional genomic introgression facilitates the colonization of an invasive orchid in arid, metal-enriched sedimentary habitats

**DOI:** 10.1016/j.xplc.2025.101561

**Published:** 2025-10-13

**Authors:** Zhenbin Jiao, Zhiyao Ren, Chao Hu, Xiaokai Ma, Guo-Qiang Zhang, Li-Jun Chen, Gang Wei, Dong-Hui Peng, Siren Lan, Yi-Bo Luo, Zhong-Jian Liu

**Affiliations:** 1 Key Laboratory of Orchid Conservation and Utilization of National Forestry and Grassland Administration at College of Landscape Architecture and Art, Fujian Agriculture and Forestry University, Fuzhou 350002, China; 2 State Key Laboratory of Systematic and Evolutionary Botany, Institute of Botany, Chinese Academy of Sciences, Beijing 100093, China; 3 University of Chinese Academy of Sciences, Beijing 100049, China; 4 Guangzhou Geriatric Hospital, Guangzhou 510180, China; 5 Eastern China Conservation Centre for Wild Endangered Plant Resources, Shanghai Chenshan Botanical Garden, Shanghai 201602, China; 6 Center for Genomics and Biotechnology, Haixia Institute of Science and Technology, School of Future Technology, Fujian Agriculture and Forestry University, Fuzhou 350002, China; 7 Shenzhen Key Laboratory for Orchid Conservation and Utilization and The National Orchid Conservation Center of China, The Orchid Conservation and Research Center of Shenzhen, Shenzhen 518114, China; 8 School of Pharmaceutical Sciences, Guangzhou University of Chinese Medicine, Guangzhou 510006, China

**Keywords:** introgression, invasive species, distantly related species, metal-ion stress, *Dendrobium*

## Abstract

Genes that introgress between species can influence the evolutionary and ecological fate of recipients exposed to novel environments. However, key questions on the patterns and molecular mechanisms of introgression in perennial herbaceous plants, which enable distantly related invasive species to thrive in extreme habitats, remain largely unanswered. Here, we report unidirectional introgression from the local species *Dendrobium huoshanense* to the distantly related invasive species *Dendrobium catenatum* (*Dendrobium officinale*) in lithophytic habitats of eastern China. The introgressed regions, which comprise approximately 1% of the genome, contain genes that regulate responses to drought, cold, and metal-ion stresses. Notably, introgressed loci such as *CDPK*, *HHP*, *PIF*, *BRI1*, and *FY* show distinct selection signatures and differential expression compared with their paralogs, each playing a distinct role in drought and cold-stress responses. In addition, *CIPK23*, *PDR9*, and *HAM* demonstrate differential expression relative to their paralogous genes and alleles within introgressed loci, indicating their potential involvement in responses to metal-ion stress. Introgression thus facilitates the colonization of arid, metal-enriched sedimentary habitats by *D. catenatum*. These findings enhance our understanding of Orchidaceae evolution and reveal the evolutionary role of unidirectional introgression in the adaptation of perennial herbaceous plants to extreme environments.

## Teaser

This study demonstrates unidirectional genomic introgression from *Dendrobium huoshanense* to the invasive species *D. catenatum* in lithophytic habitats. The introgressed regions harbor genes that regulate responses to drought and metal-ion stress, enabling *D. catenatum* to colonize arid, metal-enriched sedimentary environments.

## Introduction

Adaptation is crucial for plant survival in diverse habitats. When species colonize new habitats through natural or human-assisted dispersal, they can experience significant changes in their environment, resulting in population declines and increased selection pressures. Adaptation requires genetic variation from new mutations, standing genetic variation, and gene flow (VanWallendael et al., 2019). However, new mutations arise slowly, and standing genetic variation may be insufficient. A declining population size coupled with immediate threats to fitness may constrain the sources of genetic variation.

Introgression serves as an important mechanism for transferring genetic variation among established groups, enabling the rapid reshuffling of diverse adaptations and complex modifier systems (Oziolor et al., 2019; Edelman and Mallet, 2021). In nature, the occurrence and direction of introgression can be influenced by factors such as pre- and post-zygotic isolation and population size (Viard et al., 2020). A long-standing concern is that introgression occurs primarily from invasive species to closely related native species. However, we remain confronted by our ignorance of the fundamental evolutionary processes by which introgression from native species facilitates the colonization of extreme environments by newcomers, particularly in the case of distantly related species. Interestingly, perennial herbaceous plants have traits that may promote introgression, such as outcrossing and incomplete reproductive isolation, perennial habit, and apomictic or vegetative reproduction (Ellstrand et al., 1996).

The Danxia landform is classified as a red terrestrial clastic rock formation, characterized by distinctive red cliffs and escarpments. This landform is found primarily in China, North America, Europe, and Australia and has long been recognized as a significant component of the Earth’s surface ecosystem and environment (Peng, 2020). The red stratified sediments of the Danxia landform are characterized by high levels of metal ions, including iron (Fe^3+^), calcium (Ca^2+^), and magnesium (Mg^2+^) (Zhu et al., 2015; Xiao et al., 2018). This region experiences significant annual fluctuations in temperature and humidity and has a limited capacity for water storage (Ma et al., 2006). The Danxia landform has been recognized as a “natural laboratory” for studying responses to extreme environmental stresses, including drought, extreme temperatures, and elevated levels of metal ions.

Orchids (Orchidaceae) comprise over 25 000 species, accounting for approximately 10% of the world’s flowering plants, and have successfully colonized almost every habitat on Earth (Roberts and Dixon, 2008; Givnish et al., 2016). Epiphytic orchids comprise 67.6% of all epiphytic species (21 169 out of 31 311) (Zotz et al., 2021) and nearly all belong to the Epidendroideae (Zotz and Winkler, 2013), the largest subfamily within the Orchidaceae. The genus *Dendrobium* (Epidendroideae, Orchidaceae) is among the largest genera of orchids, found primarily in Asia and Oceania and exhibiting epiphytic and lithophytic habits (Wood, 2006; Chen et al., 2009; Pridgeon et al., 2014). Similar to other orchid species, *Dendrobium* species produce small, wind-dispersed seeds that lack an endosperm and exhibit incomplete reproductive barriers that may facilitate both dispersal and introgression (Pinheiro et al., 2015; Zhang et al., 2017; Kotilínek et al., 2020). Both nuclear and organellar phylogenies have revealed inconsistencies at several nodes in *Dendrobium* (Wang et al., 2023). *Dendrobium huoshanense* (2n = 2x = 38) exhibits a lithophytic habit and is found primarily in the Dabieshan, Huangshan, Longhushan, and Funiushan mountain ranges of eastern China (Tang and Cheng, 1984; Niu et al., 2020; Jiao and Luo, 2021)*. Dendrobium catenatum* (*Dendrobium officinale*) (2n = 2x = 38) is distantly related to *D. huoshanense*, having diverged from a common ancestor approximately 6.4 million years ago (Mya) (Xiang et al., 2016). It exhibits both epiphytic and lithophytic habits and likely originated in the South Yungui Plateau before dispersing to eastern China (Hou et al., 2017). *D. catenatum* populations that persist in the habitats of the Danxia landform exhibit resistance to extreme environmental conditions and have physiological characteristics distinct from those of populations in southwestern China (Ren et al., 2020). However, little is known about the molecular basis of their apparently evolved resistance to drought, extreme temperatures, and elevated levels of metal-ion stress in the Danxia landform of eastern China.

In this study, we aimed to address the following questions regarding the two distantly related species: (1) Does *D. catenatum* exhibit signatures of introgression from *D. huoshanense*? (2) Is this introgression unique to populations collected from extreme environments? (3) Do the introgressed genes show evidence of response to drought, extreme temperatures, and elevated levels of metal-ion stress in sedimentary habitats? To investigate these questions, we performed an introgression analysis between the *D. huoshanense* and *D. catenatum* populations and examined the expression profiles of introgressed genes in response to abiotic stress. To explore the potential functional roles of the introgressed genes, we performed a transcriptomic analysis comparing these genes with their paralogs and alleles in the introgressed loci. This study enhances our understanding of orchid evolution and reveals that introgression from distantly related local species may serve as a significant source of adaptive variation, facilitating the rapid colonization of novel habitats by newcomers.

## Results

### Habitat shifts and population structure of *D. catenatum* and its congeneric species

**Figure 1:**
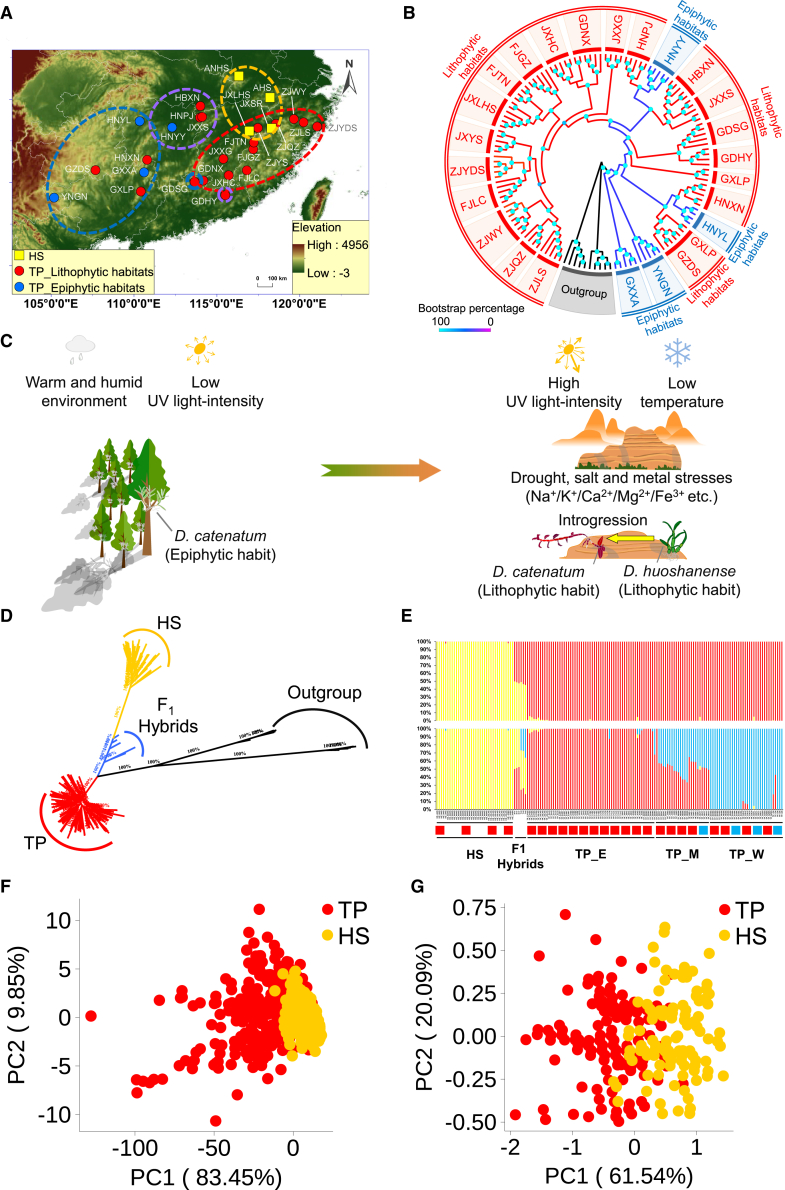
Geographic distribution, population substructure, and phenotypic diversity of *D. catenatum* and *D. huoshanense*. **(A)** Geographic distribution of *D. catenatum* and *D. huoshanense* populations in China. Symbols represent species samples: circles denote *D. catenatum*, and squares denote *D. huoshanense*. Colors represent specific habitats: red indicates lithophytic habitats, and blue indicates epiphytic habitats. Each circular curve corresponds to a specific ecotype or species: red for TP_E, purple for TP_M, blue for TP_W, and yellow for HS. **(B)** ML tree of *D. catenatum* populations, with red branches indicating lithophytic habitats and blue branches indicating epiphytic habitats. **(C)** Transition from epiphytic to lithophytic habitats of *D. catenatum*. **(D)** ML tree of *D. catenatum*, *D. huoshanense*, and artificial F_1_ hybrid populations. **(E)** Population structure inferred from an admixture analysis of *D. catenatum* and *D. huoshanense* individuals (*K* = 2–3), with red squares representing lithophytic habitats and blue squares representing epiphytic habitats. **(F)** Principal-component analysis of phenotypic data for stems, comparing *D. catenatum* (red) and *D. huoshanense* (yellow). **(G)** Principal-component analysis of phenotypic data for flowers, comparing *D. catenatum* (red) and *D. huoshanense* (yellow).

To investigate the habitat shifts and population structure of the two *Dendrobium* congeners, we sampled three populations of *D. huoshanense* (37 samples), 24 populations of *D. catenatum* (119 samples), five individuals of *Dendrobium chrysotoxum*, five individuals of *Flickingeria albopurpurea*, and six individuals of F_1_ hybrids (Figure 1A; Supplemental Figure 1; Supplemental Table 1). We performed restriction-site-associated DNA sequencing (RAD-seq) on the samples using Illumina paired-end 150-bp sequencing and generated 447.9 Gb of data in total, with an average of 2.6 Gb of reads per sample (Supplemental Table 1). These sequences were mapped to the *D. catenatum* genome, and 5 913 809 SNPs were identified.

To reveal the genetic relationships among the geo-referenced populations of *D. catenatum*, we constructed a population phylogeny for this species. The results showed that *D. catenatum* populations clustered according to their geographic locations (Figure 1B). Notably, the populations of *D. catenatum* found primarily in southwestern China were basal in the phylogenetic tree. This suggests that *D. catenatum* originated in southwestern China before dispersing to lithophytic habitats in eastern China (Figure 1B and 1C). Furthermore, *D*. *huoshanense* and *D. catenatum* belong to distinct lineages (Figure 1D).

Analysis of population structure grouped the studied individuals into two clusters: *D. huoshanense* (HS) and *D. catenatum* (TP) (Figure 1E; Supplemental Figures 2 and 3). The latter was further subdivided into three ecotypes—western (TP_W), central (TP_M), and eastern (TP_E)—at *K* = 3 (*K* = 2 was identified as the best-fitting value) (Figure 1E; Supplemental Figure 2). Populations in TP_E are primarily located in eastern China, and those in TP_W, found predominantly in southwestern China, are basal in the phylogenetic tree (Figure 1B). The mean nucleotide diversity of TP_E was 0.083, indicating a higher level of genetic diversity compared with the other two ecotypes of *D. catenatum* (Supplemental Table 2). Genetic diversity among *D. catenatum* populations increased from western to eastern China (Supplemental Figures 4 and 5; Supplemental Table 2). A lower degree of genetic differentiation was observed between TP_E and TP_M, and a higher level of genetic differentiation was noted between ecotypes that were separated by greater geographic distances, such as TP_E and TP_W (Supplemental Table 3).

### Unidirectional genomic introgression from *D. huoshanense* to *D. catenatum*

**Figure 2:**
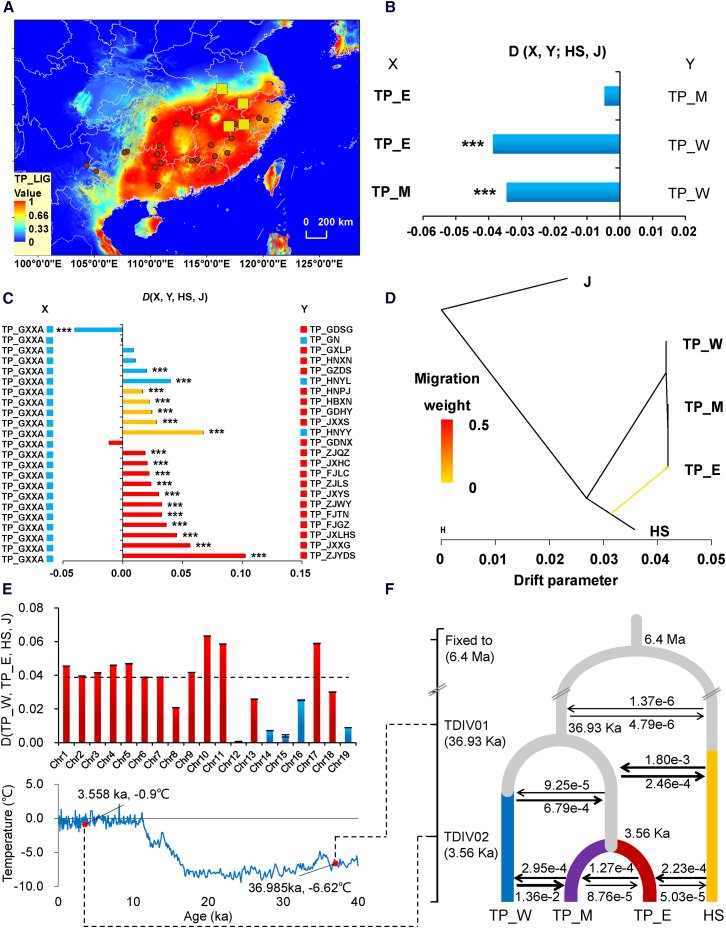
Multiple lines of evidence support gene flow from *D. huoshanense* to *D. catenatum*. **(A)** Predicted distributions of *D. catenatum* during the Last Interglacial (LIG) and the current geographic distribution of *D. huoshanense*. **(B)** The *D* statistic is denoted as *D* (X, Y; HS, J), where X and Y represent different ecotypes of *D. catenatum*. **(C)***D* (X, Y; HS, J) at all sites, where Y represents a present-day population of *D. catenatum*. Red squares denote lithophytic habitats, and blue squares denote epiphytic habitats. Asterisks denote significant differences (∗*p* < 0.05, ∗∗*p* < 0.01, ∗∗∗*p* < 0.001) based on a block jackknife approach. **(D)** An ML tree indicating a significant migration event from *D. huoshanense* (HS) to the eastern ecotype of *D. catenatum* (TP_E), with a weight of 0.0126 ± 0.0006 standard errors (*p* < 2.225 × 10^−308^). **(E)** Distribution of *D* (TP_W, TP_E, HS, J) values across chromosomes. *D* values that differ significantly from zero are represented in red, and those that do not differ significantly from zero are represented in blue. **(F)** Inferred demographic history of *D. catenatum* (TP_E, TP_M, and TP_W) and *D. huoshanense* (HS).

Multiple lines of evidence support the potential for gene flow between *D. huoshanense* and *D. catenatum*. (1) The distribution of *D. catenatum* partially overlaps with that of *D. huoshanense* in the Danxia landform of eastern China (Figure 1A). (2) Although they differ in some phenotypic traits of stems and flowers (Figure 1F and 1G; Supplemental Table 4), individuals in sympatric or parapatric populations of *D. huoshanense* and *D. catenatum* exhibit notable morphological similarities (Supplemental Figure 6). (3) Populations of the eastern ecotype of *D. catenatum* (TP_E) show a closer genetic relationship to *D. huoshanense* than do those of the other ecotypes (Supplemental Figure 7). (4) A neighbor-net tree revealed two distinct clusters between the populations of *D. catenatum* and *D. huoshanense*, demonstrating a notably higher degree of reticulation, particularly between *D. huoshanense* and the eastern ecotype of *D. catenatum* (Supplemental Figure 8). (5) Ecological niche modeling (ENM) indicated that the most suitable distribution of *D. catenatum* likely overlaps with that of *D. huoshanense* (Figure 2A; Supplemental Figures 9 and 10).

To examine gene flow between *D. huoshanense* and *D. catenatum*, we calculated Patterson’s *D* statistics using *F. albopurpurea* (J) as an outgroup. In all *D*-statistic tests, we observed a significantly negative *D* (TP_E, TP_W, HS, J) with a mean *Z* score of −18.28 (Figure 2B; Supplemental Tables 5 and 6), indicating gene flow between HS and TP_E. We analyzed Patterson’s *D* statistics between HS and various populations of *D. catenatum*. *D* (X, Y, HS, J) values greater than 0 showed significant signals (*Z* > 3.0) in most tested quadruples (Figure 2C) and displayed a negative correlation trend with geographic distance (Supplemental Figures 11 and 12), supporting the hypothesis of introgression between *D. huoshanense* and *D. catenatum* in the Danxia landform of eastern China (Supplemental Figure 13).

We examined the direction of gene flow between *D. huoshanense* (HS) and the eastern ecotype of *D. catenatum* (TP_E) by analyzing coding and non-coding loci on chromosome 1 as distinct datasets. We applied two models to the data: model I (HS to TP_E introgression) and model O (TP_E to HS introgression) (Supplemental Figure 14). For model I, the estimated introgression probability was *φ*_H_ = 0.076 (95% highest probability density (HPD) credibility interval [CI]: 0.056–0.098) for non-coding data and *φ*_H_ = 0.011 (95% HPD CI: 0.005–0.018) for coding data (Supplemental Tables 7 and 8). For model O, the estimated introgression probability was *φ*_S_ = 0.025 (95% HPD CI: 0.011–0.040) for non-coding data and *φ*_S_ = 0.021 (95% HPD CI: 0.011–0.032) for coding data (Supplemental Tables 7 and 8). We compared the two models by calculating Bayes factors through thermodynamic integration. For the coding regions, the log marginal likelihoods for model I and model O were −35 226.45 and −35 236.07, respectively, with *K* set to 16 quadrature points in Gaussian quadrature. The posterior probability ratio for models I and O (*B*_IO_) was 1.51E+04 (Supplemental Table 9). In non-coding regions, the log marginal likelihoods for model I and model O were −31 329.31 and −31 359.54, respectively. The posterior probability ratio for models I and O (*B*_IO_) was 1.34E+13 (Supplemental Table 9). A 1% cutoff was applied, with *B*_IO_ > 100 signifying support for model I. Therefore, both coding and non-coding datasets provided evidence for introgression from HS to TP_E. Moreover, TreeMix analysis indicated that 1.25% (*p* < 2.23E−308) of the genome was introgressed from HS to TP_E (Figure 2D; Supplemental Figures 15 and 16). We next investigated introgression across the chromosomes and found that 14 chromosomes exhibited significant positive *D* statistics (Figure 2E). These results indicate a unidirectional and low proportion of introgression from *D. huoshanense* to the eastern ecotype of *D. catenatum*.

On the basis of the results presented above, we used *fastsimcoal2* (Excoffier et al., 2013) to trace the divergence and gene flow between *D. huoshanense* and the three ecotypes of *D. catenatum* (Figure 2F; Supplemental Figure 17). The main findings were as follows: (1) Approximately 36.93 thousand years ago (kya), *D. catenatum* dispersed from southwestern China to central or eastern China, leading to divergence between TP_W and the common ancestor of TP_E and TP_M (Figure 2F). According to a previous study, the divergence of *D. huoshanense* from its sister species *Dendrobium henanense* is estimated to have occurred around 110 kya (Xu, 2015), coinciding with the Last Interglacial period (LIG). Based on results from this molecular clock analysis, we constructed potential distribution areas with climates suitable for *D. catenatum* in China during the LIG, the Last Glacial Maximum (LGM), the Mid-Holocene, and the present time (Supplemental Figure 9). During the LGM, the most suitable distribution of *D. catenatum* was primarily concentrated in the Danxia landform in eastern China. This distribution covered a smaller range and contained some areas of unsuitable habitat compared with the LIG (Supplemental Figure 9). The distribution of *D. catenatum* partially overlapped with that of *D. huoshanense* during the LIG. Consistent with the predicted distributions of *D. catenatum* during the LIG and LGM, we also observed gene flow between HS and the common ancestor of TP_E and TP_M. (2) Approximately 3.56 kya, the common ancestor of TP_E and TP_M colonized the Danxia landform, leading to their divergence (Figure 2F). These results suggest that gene flow occurred primarily from the local species *D. huoshanense* to *D. catenatum*.

### Population genetic characteristics of unidirectionally introgressed genomic regions

To investigate the introgression between *D. huoshanense* and *D. catenatum*, we used an ABBA–BABA model approach and calculated the *f*_dM_ introgression statistic (Martin et al., 2015). Both estimators yield positive values if introgression occurs between P2 (TP_E) and P1 (HS) and negative values if introgression occurs between P3 (TP_W) and P1 (HS) on a scale from −1 to 1. We identified 181 outlier genomic regions across the 19 chromosomes that exhibited signatures of introgression (top 5%, *f*_*dM*_ = 0.255). These regions were distributed non-randomly across the chromosomes, as indicated by the Kolmogorov‒Smirnov test (*Z* = 10.551) (Supplemental Figures 18 and 19). We next examined the population genetic indices of the introgressed regions. Approximately two-thirds of these regions exhibited higher recombination rates and increased heterozygosity. However, nearly one-third of the regions exhibited lower recombination rates and negative Tajima’s *D* values, and the average total between-species sequence divergence (*D*_xy_) was much lower than the between-species population differentiation (*F*_ST_). This suggests recent ecological selection in sedimentary habitats (Supplemental Figures 20–23; Supplemental Tables 10, 11, 12, and 13).

A total of 159 genes were identified in the introgressed genomic regions. Analysis of Gene Ontology (GO) terms and Kyoto Encyclopedia of Genes and Genomes (KEGG) annotations revealed that these genes were associated with abiotic stresses, including drought, extreme temperatures, and elevated levels of metal-ion stress (Supplemental Figure 24; Supplemental Table 14), as well as pathways such as plant-pathogen interaction, plant hormone signal transduction, and mitogen-activated protein kinase signaling (Supplemental Table 15). The orthologs of the introgressed genes in *Arabidopsis thaliana* are involved in responses to abiotic stress (Supplemental Table 16), organ development (Supplemental Table 17), DNA repair (Supplemental Table 18), and defense responses (Supplemental Table 19). The annotations of the introgressed genes thus suggest that they are associated with the response of *D. catenatum* to abiotic stress under extreme environmental conditions.

### Unidirectional introgression may enhance plant responses to drought and temperature stress

Considering that *D. catenatum* in the lithophytic habitats of eastern China shows evidence of introgression from the local species *D. huoshanense*, this unidirectional introgression is likely linked to the species’ response to extreme environmental conditions. To evaluate whether these introgressed genes are involved in abiotic stress responses, we performed differential expression analyses under drought- and temperature-stress conditions. Twenty-nine introgressed genes were differentially expressed under drought stress, and 35 were differentially expressed under temperature stress (Supplemental Figure 25). Notably, 3 of the 29 genes—*CDPK*, *HHP*, and *PIF3*—have previously been associated with drought-stress responses (Urao et al., 1994; Chen et al., 2010; Wang et al., 2020), and 4 of the 35 genes—*BRI1*, *HHP*, *PIF3*, and *FY*—have been linked to the cold-stress response (Simpson et al., 2003; Oh et al., 2009; Chen et al., 2010; Wang et al., 2020).

**Figure 3:**
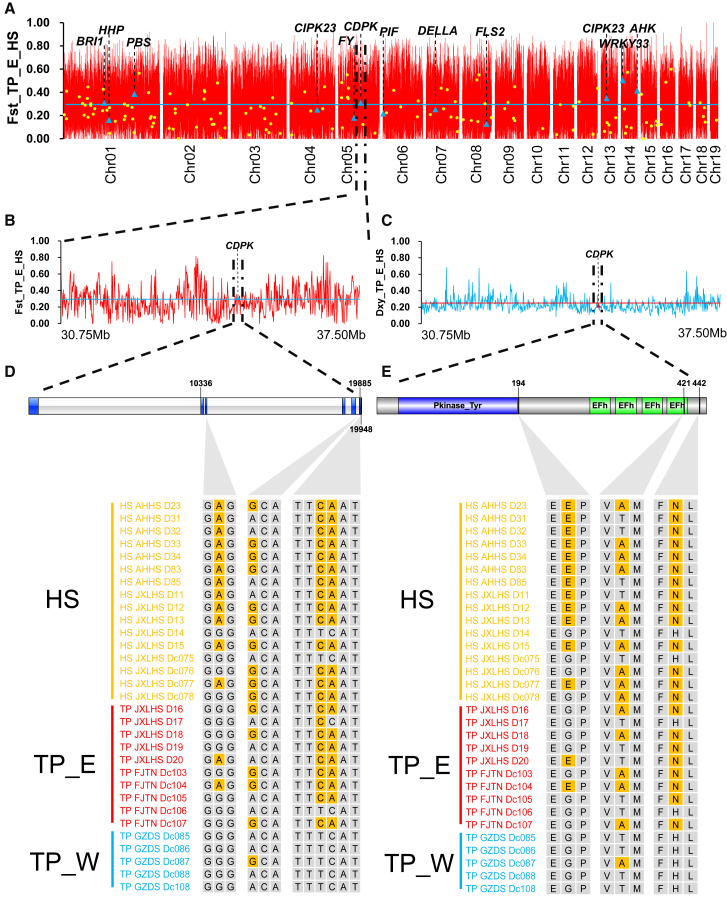
Evidence of introgression at the *CDPK* locus. **(A)** Fixation index (*F*_ST_) values were calculated in 10-kb windows across 19 chromosomes, comparing the eastern ecotype of *D. catenatum* (TP_E) with *D. huoshanense* (HS). **(B and C)** Fixation index (*F*_ST_) values **(B)** and absolute divergence (*D*_xy_) values **(C)** across the *CDPK* region. **(D and E)** Representative substitutions of *CDPK* in samples of *D. huoshanense* (yellow) and the eastern (red) and western (blue) ecotypes of *D. catenatum*. Substitutions (brown) in nucleotides **(D)** and amino acids **(E)** were observed in representative populations of *D. catenatum* and *D. huoshanense.* The horizontal line indicates the average value across the genome.

**Figure 4:**
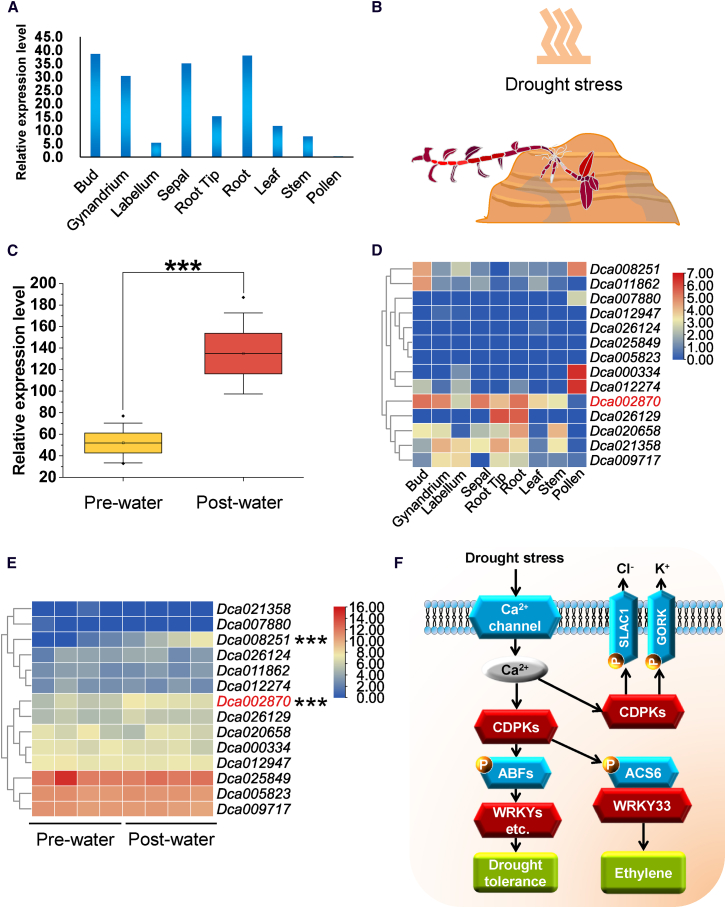
Changes in *CDPK* expression are correlated with responses to abiotic stress in *D. catenatum*. **(A)** Expression of *CDPK* across nine tissues of *D. catenatum*. **(B)** Image of *D. catenatum* under drought stress. **(C)** Boxplot illustrating the differential expression of *CDPK* under drought stress. **(D)** Heatmap illustrating the expression of *CDPK* and its paralogous genes in nine tissues. **(E)** Heatmap illustrating the expression of *CDPK* and its paralogous genes under drought stress. **(F)** A hypothetical drought-stress signaling pathway in *D. catenatum*. Introgressed genes are highlighted in red. Asterisks denote significant differences (∗*p* < 0.05, ∗∗*p* < 0.01, ∗∗∗*p* < 0.001) based on the Benjamini–Hochberg procedure.

*CDPK* likely enhances the response of *D. catenatum* to drought stress. Here, we found that *CDPK* exhibited relatively large genetic divergence (*F*_ST_ = 0.31, *D*_xy_ = 0.22) and contained nonsynonymous mutations observed in TP_E and HS. At these loci, some individuals from the eastern ecotype of *D. catenatum* and *D. huoshanense* were grouped by geographic distribution patterns (Figure 3; Supplemental Figure 26). Molecular diversity analysis indicated that the Tajima’s *D* value for *CDPK* was negative (Tajima’s *D* = −2.0898, *p* < 0.05; Fu and Li’s *D* = −3.0423, *p* < 0.05; Fu and Li’s *F* = −3.1173, *p* < 0.05; *K*_a_/*K*_s_ ratio < 1, *p* value [Fisher] < 0.05) (Supplemental Figure 27). These findings indicate that *CDPK* has been subjected to negative (purifying) selection in *D. catenatum* populations. In addition, *CDPK* exhibited higher expression levels in the roots, buds, sepals, and gynandrium (Figure 4A), suggesting its association with root and flower development. Differential expression analyses revealed that *CDPK* expression was significantly higher under post-watering conditions, with a 2.667-fold increase (adjusted *p* = 1.3E−06) (Figure 4B and 4C). We also examined the expression of *CDPK*’s paralogs (Figure 4D and 4E). They displayed various expression levels across nine tissues and were distinct from those of *CDPK* (Figure 4D). Notably, one paralog (*Dca008251*) exhibited significantly higher expression under post-watering conditions, with an adjusted *p* value greater than that of *CDPK* (Figure 4E). These results indicate that *CDPK* likely enhances the response of *D. catenatum* to drought stress (Figure 4F).

*HHP* and *PIF3* likely enhance the response of *D. catenatum* to both drought and temperature stress. *HHP* exhibited relatively large genetic divergence (*F*_ST_ = 0.16, *D*_xy_ = 0.15) and contained synonymous mutations observed in TP_E and HS (Supplemental Figure 28). Molecular diversity analysis indicated that the Tajima’s *D* value for *HHP* was negative (Tajima’s *D* = −1.0126, 0.10 < *p*; Fu and Li’s *D* = −2.2301, 0.05 < *p* < 0.10; Fu and Li’s *F* = −2.0512, 0.05 < *p* < 0.10; *K*_a_/*K*_s_ ratio < 1, *p* value [Fisher] < 0.05) (Supplemental Figure 27). These findings suggest that *HHP* has been subjected to negative selection in *D. catenatum* populations. *HHP* exhibited significantly lower expression levels under drought stress (adjusted *p* = 1.3E−02) and low-temperature conditions (adjusted *p* = 5.8E−04) (Supplemental Figure 29). Notably, its two paralogs (*Dca002032* and *Dca023806*) exhibited the opposite expression pattern to *HHP* under drought-stress conditions (Supplemental Figure 29). These results indicate that *HHP* may enhance the response to drought and temperature stress. Furthermore, *PIF3* exhibited relatively large divergence (*F*_ST_ = 0.22, *D*_xy_ = 0.13) and contained synonymous mutations observed in TP_E and HS (Supplemental Figure 30). Molecular diversity analysis indicated that the Tajima’s *D* value for *PIF3* was negative (Tajima’s *D* = −1.1440, 0.10 < *p*; Fu and Li’s *D* = −1.1833, 0.10 < *p*; Fu and Li’s *F* = −1.3951, 0.10 < *p*; *K*_a_/*K*_s_ ratio < 1, *p* value [Fisher] < 0.05) (Supplemental Figure 27), suggesting that *PIF3* may have been subjected to soft selection in *D. catenatum* populations. *PIF3* showed significantly lower expression levels under drought stress (adjusted *p* = 2.9E−02) and low-temperature conditions (adjusted *p* = 1.5E−03) (Supplemental Figure 31). Notably, one paralog (*Dca004599*) of *PIF3* exhibited a significantly lower expression level under low-temperature conditions, with a greater adjusted *p* value than that of *PIF3* (Supplemental Figure 31). These findings indicate that *HHP* and *PIF3* likely enhance the responses of *D. catenatum* to both drought and temperature stress.

*FY* and *BRI1* may be associated with flowering, which responds indirectly to cold stress. *FY* exhibited relatively large divergence between TP_E and HS (*F*_ST_ = 0.18, *D*_xy_ = 0.13) and contained mutations observed in both TP_E and HS (Supplemental Figure 32). It was likely subjected to negative selection in *D. catenatum* populations (Tajima’s *D* = −1.7365, 0.02 < *p* < 0.10; Fu and Li’s *D* = −2.7288, *p* < 0.05; Fu and Li’s *F* = −2.7178, *p* < 0.05; *K*_a_/*K*_s_ ratio < 1, *p* value [Fisher] < 0.05) (Supplemental Figure 27). *FY* showed a higher expression level in pollen and significantly lower expression under low-temperature conditions (adjusted *p* = 5.7E−05) (Supplemental Figure 32). *BRI1* also exhibited relatively large divergence between TP_E and HS (*F*_ST_ = 0.31, *D*_xy_ = 0.12) and contained nonsynonymous mutations observed in both TP_E and HS (Supplemental Figure 33). It was likely subjected to soft selection in *D. catenatum* populations (Tajima’s *D* = −0.6625; Fu and Li’s *D* = −2.1892, 0.05 < *p* < 0.10; Fu and Li’s *F* = −1.8128, *p* < 0.10; *K*_a_/*K*_s_ ratio < 1, *p* value [Fisher] < 0.05) (Supplemental Figure 27). *BRI1* exhibited higher expression under low-temperature conditions (adjusted *p* = 5.7E−04) (Supplemental Figure 34). Consistent with the putative functional roles and expression patterns of *FY* and *BRI1*, the flowering time of TP_E was much later than that of TP_W (Supplemental Figure 35). Collectively, these results indicate that unidirectional introgression may have enhanced the responses of *D. catenatum* to drought and temperature stress.

### Unidirectionally introgressed genes may underlie the acquisition and homeostasis of metal ions

**Figure 5:**
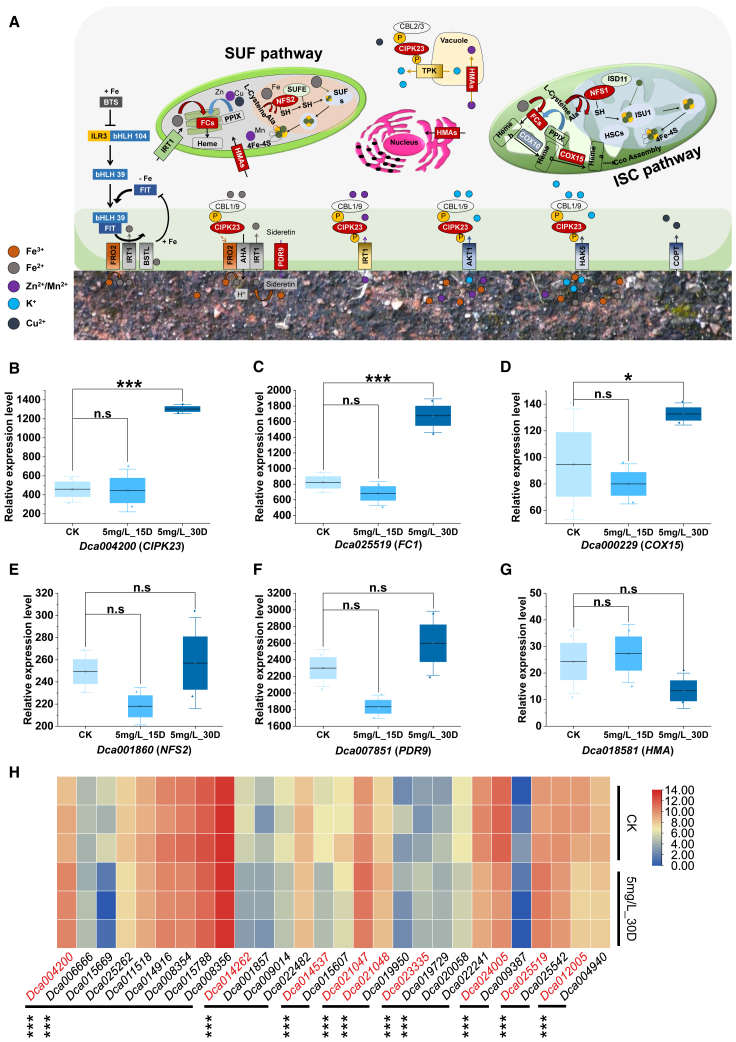
Introgressed genes respond to various metal-ion stresses by regulating transporters and ion channels. **(A)** Image illustrating the intracellular uptake and homeostasis of metal ions across various cellular organelles, including mitochondria, chloroplasts, and vacuoles. Circular dots represent metal ions, and arrows indicate the direction of metal-ion transport, which is essential for maintaining metal homeostasis. Elements highlighted in red denote introgressed genes or proteins. **(B–G)** Boxplots illustrating the differential expression of introgressed genes associated with metal-ion stress under various conditions of metal-ion stress. **(H)** Heatmap illustrating the expression of introgressed genes and their paralogous genes under cadmium (Cd)-stress conditions. Asterisks denote significant differences (∗*p* < 0.05, ∗∗*p* < 0.01, ∗∗∗*p* < 0.001) based on the Benjamini–Hochberg procedure and log2 fold change (log_2_FC). n.s., no significant difference (*p* > 0.05).

To identify introgressed genes involved in pathways related to metal-ion uptake and homeostasis, we performed BLAST and KEGG annotation analyses. This process led to the identification of introgressed genes encoding three enzymes responsible for metal uptake from the rhizosphere, one enzyme for transport within organelles, and three enzymes that facilitate various cellular processes. The identified genes included *CIPK23*, *PDR9*, *HMA*, *FC1*, *NFS2*, and *COX15* (Figure 5A).

To determine whether the introgressed genes were indeed involved in the response to metal-ion stress, we analyzed the transcriptome profiles of *D. catenatum* roots treated without Cd (control, CK) or with 5 mg/L Cd for 15 (5 mg/L_15D) or 30 days (5 mg/L_30D). Compared with the CK, 1085 differentially expressed genes (DEGs) were identified in the 5 mg/L_30D treatment. More DEGs were detected after 30 days than after 15 days (Supplemental Figure 36), indicating a greater number of transcriptional changes at 30 days. In particular, the DEGs between CK and the 5 mg/L_30D treatment included nine introgressed genes, suggesting that these genes may be involved in the response of *D. catenatum* to Cd stress (Supplemental Figure 36). Interestingly, proteins encoded by six of these genes—*CIPK23*, *FC1*, *COX15*, *NFS2*, *PDR9*, and *HMA*—participate in metabolic pathways that play a role in the uptake, transport, and detoxification of metal ions (Figure 5B–5G). Previous studies have also indicated that *PDR9* is involved in the response to iron (Fe) deficiency (Fourcroy et al., 2014). *COX15*, *NFS2*, and *HMA* are involved in the transport of metal ions and the biosynthesis of heme, likely responding to metal-ion stress through indirect mechanisms. Consequently, *CIPK23* and *FC1* exhibited significant differential expression under conditions of metal-ion stress (Figure 5B and 5C).

To investigate the potential functional role of the introgressed genes, we compared their expression with that of their paralogs and alleles from *D. huoshanense* (HS-derived) under various Cd-stress conditions. Paralogs of the nine genes were identified and displayed various expression levels (Figure 5H). Notably, one paralog of *Dca004200* and one paralog of *Dca023335* exhibited significantly lower expression under 5 mg/L_30D conditions (Figure 5H) compared with *Dca004200* and *Dca023335*, respectively. The adjusted *p-*values for the two genes were significantly lower than those of their paralogs (Figure 5H).

**Figure 6:**
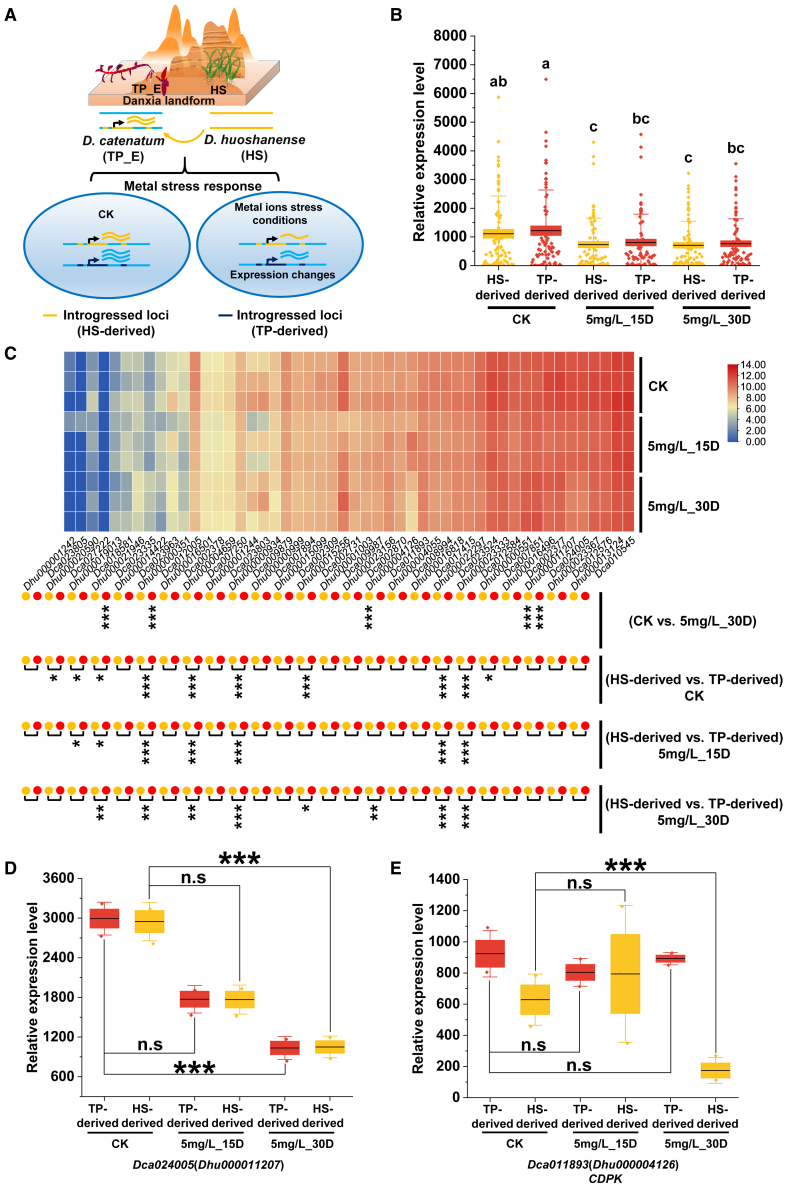
Differential expression of introgressed genes derived from *D. huoshanense* (HS-derived) and their corresponding genes from *D. catenatum* (TP-derived) under various cadmium (Cd)-stress conditions. **(A)** Schematic illustration depicting the introgressed genes from *D. huoshanense* (HS-derived) and their corresponding genes from *D. catenatum* (TP-derived) in response to different metal-ion stresses. **(B and C)** Boxplot **(B)** and heatmap **(C)** displaying the expression levels of introgressed genes under various Cd-stress conditions. Genes derived from *D. huoshanense* are shown as yellow solid circles, and the corresponding genes derived from *D. catenatum* are shown as red solid circles. Significant differential expression of genes between the CK and the 5-mg/L_30D treatment is indicated by the adjusted *p* value. Significant differential expression between the introgressed genes from *D. huoshanense* (HS-derived) and their corresponding genes from *D. catenatum* (TP-derived) under various Cd-stress conditions is indicated by lowercase letters. Different lowercase letters indicate statistically significant differences according to a one-way ANOVA followed by Fisher’s least significant difference test (*p* < 0.05). **(D)** Boxplot illustrating the relative expression levels of *Dca024005* (TP-derived) and *Dhu000011207* (HS-derived) under various Cd-stress conditions. **(E)** Boxplot illustrating the relative expression levels of *Dca011893* (TP-derived) and *Dhu000004126* (HS-derived) under various Cd-stress conditions. Asterisks denote significant differences (∗*p* < 0.05, ∗∗*p* < 0.01, ∗∗∗*p* < 0.001) based on the Benjamini–Hochberg procedure and log2 fold change (log2FC). n.s., no significant difference (*p* > 0.05).

In addition, we identified the introgressed alleles derived from *D. huoshanense* (HS-derived) and compared the expression levels of their respective introgressed genes under various Cd-stress conditions (Figure 6A). A total of 4640 genes from the transcriptomic data of *D. catenatum* were mapped to the genomic reference of *D. huoshanense*, and 324 DEGs were identified between the CK and 5 mg/L_30D treatments (Supplemental Figure 37). Notably, 25 introgressed genes derived from *D. huoshanense* were identified and expressed. The introgressed genes were downregulated under Cd-stress conditions compared with the control treatment (Figure 6B). Notably, 11 of the introgressed genes demonstrated differential expression under various Cd-stress conditions (Figure 6C). This observation suggests potential functional differences between the introgressed genes and their corresponding alleles.

Two introgressed genes displayed differences in expression under various Cd-stress conditions (Figure 6D and 6E). When compared with the CK, the adjusted *p*-value of *Dhu000011207* was 70 times lower than that of *Dca024005* in the 5 mg/L_30D treatment. Notably, the introgressed gene *CDPK*, which is involved in the response to drought stress, was differentially expressed under Cd stress and showed lower expression than its allele. These results indicate that unidirectional introgression from *D. huoshanense* may have enhanced the ability of *D. catenatum* to thrive in habitats with high metal-ion concentrations.

### Genomic signatures of selection and local adaptation

We identified 255 genes under selection in *D. huoshanense* (HS) and the eastern ecotype of *D. catenatum* (TP_E). Functional enrichment analysis revealed that some of these genes were significantly enriched in KEGG pathways related to transfer RNA biogenesis (ko03016, *p* = 1.20E−02) and in the GO term “regulation of transcription elongation by RNA polymerase II” (GO:0034243, *p* = 1.76E−03) (Supplemental Tables 20 and 21). Among the genes under selection, seven (*LCMT1*, *KCS11*, *IRE1A*, *FAR3*, *ACA5*, *PGK3*, and *CAO*) were associated with responses to abiotic stress (Supplemental Figure 38), six (*XPB1*, *SMC3*, *REV3*, *RECQL2*, *ATCSA-1*, and *MER3*) were involved in regulating the response to DNA damage stimuli (Supplemental Figure 38), and seven (*VIP4*, *MADS2*, *DCL1*, *ABH1*, *ARF3*, *MKK6*, and *CAS1*) were associated with the development of reproductive organs (Supplemental Figure 38). Notably, the regulation of flowering, specification of stamen identity, and determinacy of floral meristems are critical for male gametophyte function.

For TP_E in lithophytic habitats and TP_TREE in epiphytic habitats, we identified 299 genes under selection, some of which were enriched in the transcription machinery pathway (ko03021, *p* = 1.35E−02) and GO terms related to post-transcriptional gene silencing (GO:0016441, *p* = 9.51E−05), immune effector process (GO:0002252, *p* = 6.52E−03), sites of DNA damage (GO:0090734, *p* = 1.84E−02), and calcium ion binding (GO:0005509, *p* = 1.64E−02) (Supplemental Tables 22 and 23). Nine of these genes (i.e., *MYC2*, *CCR1*, *PIAL1*, *HPT1*, *BAS1*, *MPK1*, *KAPP*, *SIP2-1*, and *AAO*) were associated with responses to abiotic stress (Supplemental Figure 38), and eleven (e.g., *RECG*, *ATRX*, *RAD5A*, *MHF2*, *BRCA2A*, and *ATM*) were involved in regulating the response to DNA damage stimuli (Supplemental Figure 38). In addition, the introgressed gene *polypyrimidine tract-binding protein homolog 1* (*PTB1*), which plays a role in pre-mRNA splicing (Rühl et al., 2012), was under strong selection. A previous study demonstrated that misexpression of *PTB* alters the alternative splicing of *PHYTOCHROME INTERACTING FACTOR 6*, which coincides with changes in abscisic acid–dependent seed germination rates (Rühl et al., 2012).

## Discussion

This study demonstrates that *D. catenatum* likely originated in epiphytic habitats in southwestern China and subsequently dispersed to lithophytic habitats in eastern China. The findings reveal unidirectional signals and a low proportion of introgression from the distantly related local species *D. huoshanense* to *D. catenatum*. The introgressed regions exhibit higher recombination rates and increased heterozygosity, and the introgressed genes are involved in responses to drought, temperature fluctuations, and metal-ion stress. Overall, these findings suggest that unidirectional introgression has likely enhanced the ability of *D. catenatum* to colonize extreme habitats.

### Habitat shifts create opportunities for unidirectional introgression

Habitat shifts can disrupt geographic isolation and create opportunities for gene flow. In general, habitat interruptions impose significant barriers to species dispersal and have influenced phylogeographic patterns of genetic differentiation across geographic distances (Sefc et al., 2017). When species invade and occupy a new habitat, they are likely to come into contact and hybridize with local relatives. *Dendrobium* species, like most epiphytic orchids, produce dust-like, windborne seeds, which may facilitate dispersal and habitat shifts (Kotilínek et al., 2020). In this study, we found that *D. huoshanense* thrives on the sediment surface of the Danxia landform in eastern China. Population phylogenetic analysis suggested that *D. catenatum* expanded from epiphytic habitats in southwestern China to the Danxia landform in eastern China. The expansion routes of *D. catenatum* from western to eastern China align with the aforementioned findings. After occupying lithophytic habitats, *D. catenatum* likely encountered and interbred with the local, distantly related species *D. huoshanense*. Similar to other orchid species, the seeds of *D. catenatum* lack endosperm and thus depend exclusively on mycorrhizal fungi for germination in their natural environments. This dependence results in a significantly reduced germination rate, which is further limited by the availability of suitable habitats (McCormick et al., 2018). Moreover, pollen dispersal is constrained by the ability of pollinators to fly between geographically isolated populations (Michener, 1979). This constraint poses a challenge to the survival and long-distance dispersal of *D. catenatum* in nature.

The dust-like seeds of *D. catenatum* are unlikely to disperse rapidly through natural mechanisms from the Yungui Plateau to eastern China. We found that haplotypes of *D. catenatum* common in some populations of eastern China were also present in western China (Supplemental Figure 26). However, ENM analysis indicated a lack of suitable habitats between the Yungui Plateau and the Danxia landform habitats in eastern China. The genetic diversity of the eastern ecotype of *D. catenatum* is similar to that of the western ecotype found in the putative source regions. The eastern and western ecotypes of *D. catenatum* exhibit much lower genomic divergence, with *F*_ST_ values being significantly higher in several genomic regions (e.g., Chr14), indicating recent ecological selection. Previous studies have also suggested that haplotypes prevalent in populations of eastern China are unlikely to have dispersed naturally from populations in southwestern China (Hou et al., 2017). *Dendrobium* species have been used as traditional medicines in China for many centuries. For the past 100 years, humans have been collecting and processing *D. catenatum* throughout the country, facilitating its dispersion (Hou et al., 2017). These findings suggest that natural colonization of *D. catenatum* is likely to have been strongly limited by the availability of suitable habitats. Historical human-mediated transfer therefore appears to be the predominant mechanism facilitating long-distance geographic range expansion.

### A lower proportion of introgression occurs between distantly related species that exhibit incomplete reproductive isolation

A lower proportion of introgression occurs between distantly related species that exhibit incomplete reproductive isolation. Introgression is common in nature but is typically hindered by reproductive isolation (Edelman and Mallet, 2021). Evidence of introgression is primarily observed in closely related species. We continue to face challenges in understanding the genomic characteristics of introgression between distantly related species. In this study, we observed a low proportion of introgression from the local species *D. huoshanense* to *D. catenatum* in the Danxia landform of eastern China. According to previous studies, *Dendrobium* exhibits incomplete reproductive barriers (Pinheiro et al., 2015). *D*. *catenatum* and *D. huoshanense* are distantly related species, classified within the *Catenatum* group and the *D. moniliforme* complex, respectively (Xiang et al., 2013). The estimated time of their initial divergence from a common ancestor is approximately 6.4 mya (Xiang et al., 2016).

Introgressed regions can have various evolutionary fates in the recipient species. In general, introgressed haplotypes are often fragmented by recombination after initial introgression. Introgressed variants that have negative fitness effects are more likely to be eliminated in regions with lower recombination rates, whereas adaptive introgressed alleles may reach fixation (Martin and Jiggins, 2017). In this study, introgressed regions were distributed non-randomly in the genome, with approximately two-thirds of these regions exhibiting higher recombination rates and increased heterozygosity. Nearly one-third of the regions displayed lower recombination rates and negative Tajima’s *D* values, and the average total between-species sequence divergence (*D*_xy_) was much lower than the between-species population differentiation (*F*_ST_). This suggests that the introgressed regions may be influenced by recent ecological selection. Furthermore, *Dendrobium* is an outcrossing perennial that exhibits reproductive strategies promoting hybridity, such as vegetative propagation, which aids in the preservation of introgressed variants (Ellstrand et al., 1996). Consequently, a low proportion of introgression and introgressed genes with various evolutionary trajectories may be prevalent among distantly related species.

### Unidirectional introgression from local species into invasive species

According to previous studies, a long-standing concern is that introgression occurs primarily from invasive species to closely related native species. This process may lead to a loss of genetic integrity in native species whose habitats are invaded by these newcomers (Frank and Scott, 2011). In contrast to this concern, a recent study demonstrated asymmetric introgression from the native *Helicoverpa zea* into the invasive *Helicoverpa armigera* in Brazil (Valencia-Montoya et al., 2020). Another study revealed slight but detectable introgression of native *Mytilus trossulus* alleles into the genome of the invading *Mytilus galloprovincialis* (Saarman and Pogson, 2015). A model proposed by Currat et al. suggests that when one species colonizes an area already occupied by a closely related species, significant introgression of neutral genes is likely to occur primarily from the local species to the invasive species (Currat et al., 2008). In addition, Zhang developed a model that illustrates symmetric introgression, which contrasts with the substantial and asymmetric introgression predicted by the original model of Currat et al. (Zhang, 2014). In the present study, we identified introgression from the local species *D. huoshanense* to *D. catenatum*, providing new genomic evidence for introgression from local species into newcomers.

Population density and flowering time are likely to be two primary factors that influence the direction of introgression. In this study, genetic diversity analyses suggested that *D. catenatum* exhibited lower abundance than the local species *D. huoshanense*, and unidirectional introgression was observed from *D. huoshanense* into *D. catenatum*. *D. huoshanense* flowers earlier than the populations of *D. catenatum* found in eastern China. During the flowering period, the number of flowering individuals in *D. huoshanense* surpassed that in *D. catenatum*. Consequently, gene flow likely occurs from the dense population of *D. huoshanense* into the sparser population of *D. catenatum*.

### Unidirectional introgression may enable orchids to thrive in harsh, arid sediments

When species disperse and colonize new habitats, they encounter various abiotic stresses that limit their growth and development (An et al., 2024). The Danxia landform experiences significant annual fluctuations in temperature and humidity and has a limited capacity for water storage (Ma et al., 2006). In general, the genomes of native species are already adapted to their local environment, whereas those of invasive species must contend with new environmental conditions. Consequently, invasive species may exploit pre-existing adaptive alleles from native species (Viard et al., 2020). This study identified introgressed loci associated with responses to abiotic stress, including *CDPK*, *HHP*, *PIF*, *BRI1*, and *FY*. These genes are recognized as key components of responses to drought and cold stress in *A. thaliana* (Urao et al., 1994; Simpson et al., 2003; Zhu et al., 2007; Oh et al., 2009; Chen et al., 2010; Wang et al., 2020).

Introgressed genes likely enhance the response to abiotic stress through regulated expression. In this study, a *CDPK* haplotype was shared between *D. huoshanense* and the eastern ecotype populations of *D. catenatum*. Nonsynonymous mutations in the coding regions and EFh domain of *CDPK* are crucial for calcium ion binding and may influence the expression of *CDPK*. *CDPK* exhibited reduced expression under drought-stress conditions, similar to the expression of its orthologous gene in *A. thaliana*. In addition, *CDPK* exhibited distinct expression patterns compared with its paralogs in various tissues and demonstrated contrasting expression trends under drought-stress conditions, suggesting a unique role in the response to abiotic stress. In addition, haplotypes of *HHP* and *PIF3* are shared between *D. huoshanense* and the eastern ecotype of *D. catenatum* populations. Both genes exhibited reduced expression levels under drought and temperature-stress conditions. They also demonstrated distinct expression patterns compared with their paralogs across various tissues and displayed different expression trends relative to their paralogs under abiotic stress. Therefore, each of these genes may play a unique role in responses to abiotic stress through differences in expression regulation.

*BRI1* and *FY* are likely to be involved in regulating flowering time and responding to abiotic stress through indirect mechanisms. *BRI1* and *FY* play central roles in regulating plant phenology signaling pathways (Simpson et al., 2003; Oh et al., 2009). In this study, *D. catenatum* populations from the Danxia landform in eastern China flowered later than the western ecotype, consistent with previous studies. *BRI1* and *FY* haplotypes are shared between *D. huoshanense* and the eastern ecotype populations of *D. catenatum*. Under cold conditions, they exhibited increased and decreased expression, respectively. Therefore, introgressed genes may play indirect roles in responding to abiotic stress through modulation of gene expression.

### Unidirectional introgression may enhance the response of orchids to high-metal sediments

Metal ions, including calcium (Ca^2+^), iron (Fe^3+^), and magnesium (Mg^2+^), are essential micronutrients needed for a variety of physiological processes. However, these metals can be toxic at supraoptimal concentrations. When plants grow in high-metal habitats, they must develop finely tuned homeostatic mechanisms. Metal homeostasis includes transportation, chelation, and sequestration processes (Clemens, 2001), as well as the regulation of transporters or enzymes to ensure proper delivery and distribution of metal ions, resulting in a basic level of metal tolerance. Here, we identified introgressed genes associated with metal homeostasis, including *CIPK23*, *PDR9*, *FC*, *COX15*, *HMA*, and *NFS2* (Balk and Lobréaux, 2005; Verbruggen et al., 2009; Fourcroy et al., 2014; Brzezowski et al., 2015; Espinas et al., 2016; Meyer et al., 2019; Ródenas and Vert, 2021).

These introgressed genes may regulate the uptake of metal ions through expression regulation. The CIPK23 kinase serves as a crucial hub in mediating root responses to magnesium, ammonium, and non-iron metal toxicities (Ródenas and Vert, 2021). Notably, *CIPK23* regulates the IRT1 root transporter, a broad-spectrum, high-affinity metal transporter that facilitates uptake of not only Fe^2+^ but also closely related divalent metals, including Zn^2+^, Mn^2+^, Co^2+^, and Cd^2+^ (Vert et al., 2002; Barberon et al., 2011). In this study, differential expression analysis indicated that *CIPK23* was likely involved in the response to metal-ion stress. Coumarins have also emerged as key players in the solubilization and uptake of iron (Fe). A previous study reported that the *PDR9* gene is upregulated in response to Fe deficiency (Fourcroy et al., 2014). In this study, *PDR9* was not significantly differentially expressed under metal-ion stress conditions, suggesting that *D. catenatum* may have reduced iron uptake on the Danxia landform. Thus, introgressed genes may enhance the response of orchids to high-metal sedimentary habitats through regulated expression.

Introgressed genes may enhance the transport and storage of metal ions in cellular organelles through regulated expression. Organelles such as chloroplasts and mitochondria require metal ions (e.g., iron) to carry out various metabolic processes and serve as reservoirs for the storage of metal ions for later use. Here, we identified the introgressed genes *FC*, *COX15*, *HMA*, and *NFS2*, which are related to the transport of metal ions and the biosynthesis of heme. For heme biosynthesis, *FC1* supplies heme at all growth stages (Brzezowski et al., 2015; Espinas et al., 2016). *COX* is involved in oxidative phosphorylation and catalyzes the transfer of electrons from cytochrome *c* to molecular oxygen. Specifically, *COX10* and *COX15* modify heme to produce heme *a* and heme *a3* in terrestrial plants (Meyer et al., 2019). *HMA* plays a crucial role in the transport of metal ions (Verbruggen et al., 2009). Results from null mutants and overexpression experiments indicate that *HMA4* is involved in zinc homeostasis, Cd detoxification, and the translocation of these metals from roots to shoots (Hussain et al., 2004; Verret et al., 2004; Mills et al., 2005). *NFS2* is involved in the biosynthesis of iron–sulfur clusters in the iron–sulfur cluster and sulfur mobilization pathways (Balk and Lobréaux, 2005). Our differential expression results suggest that *FC* and *COX15* play a role in regulating the pathways responsible for transport and storage of metal ions within cellular organelles. These findings suggest that introgressed genes may improve the orchid’s response to habitats with high-metal sediments through their expression regulation.

This paper presents evidence that a low proportion of unidirectional genomic introgression from distantly related local species likely facilitates the colonization of extreme lithophytic habitats by invasive species. This study demonstrates that introgressed genes may enhance the ability of orchids to respond to abiotic stress through their expression regulation. Our findings provide insight into the evolution of Orchidaceae and clarify the molecular mechanisms underlying unidirectional introgression in response to extreme environments faced by perennial herbaceous plants. The genome-wide introgression data developed in this study provide an opportunity to evaluate its functional legacy. Nonetheless, the study has several limitations: the RAD-seq data exhibit lower genomic coverage, and the sample sizes for the different species are unequal. These factors may affect the accurate estimation of certain population genomic parameters. Further research using high-depth sequencing, larger sample sizes, and molecular experiments focused on introgressed genes associated with abiotic stress will be essential to accurately determine how introgression contributes to the evolvability and diversity of Orchidaceae and other perennial herbaceous plants.

## Methods

### Sampling and genomic DNA extraction for sequencing

We collected leaf samples from 172 individuals from various geographic populations for RAD-seq: 37 *D*. *huoshanense* from three populations, 119 *D. catenatum* from 24 populations, five *D. chrysotoxum* from one population, and five *F. albopurpurea* from one population. In addition, we created F_1_ hybrids as control samples, generating six individuals through artificial hybridization in 2017. We also collected leaf samples from 44 individuals for whole-genome resequencing: 10 *D. huoshanense* individuals from a single population, 25 *D. catenatum* individuals from three populations, four F_1_ hybrids of *D. huoshanense* and *D. catenatum*, and five *F. albopurpurea* individuals from one population. Leaf samples were collected from a common garden at the China National Orchid Conservation Center. Young leaves were used for DNA extraction and sequencing. Detailed information on the geographic distribution and data pertaining to the samples used in this study is provided in Figure 1A and Supplemental Table 1.

Genomic DNA was extracted from the leaf tissue of each sample using a plant total genomic DNA kit (Tiangen). For RAD-seq, a double digest restriction-site associated DNA (ddRAD) sequencing library was prepared using the Illumina TruSeq Nano DNA Library Prep Kit with *Ras*I and *Hae*III restriction enzymes. Both RAD-seq and resequencing were performed by Novogene in Nanjing, China, on the Illumina NovaSeq 6000 platform to generate 150-bp paired-end reads.

### Mapping and SNP calling

Raw reads were processed by Novogene, including the removal of poor-quality base calls and adapter sequences, to obtain clean data. The clean reads were aligned to the Hi-C genome of *D. catenatum* (2n = 2x = 38, 1.1 Gb) reference sequence using BWA v0.7.12-r1039 with default parameters (Li and Durbin, 2009). Uniquely mapped reads were sorted and indexed using Picard v1.56 (https://github.com/broadinstitute/picard). The Genotyper module from Sentieon Genomics software (version Sentieon-genomics-201911) was used to estimate the variants in each individual. Hard filtering of individual SNP calls was performed using the parameters -window 35 -Cluster 3 -filterName FisherStrand (FS) -filter “FS > 30.0” -filterName QualByDepth (QD) -filter “QD < 2.0” with GenomeAnalysisTK v3.8. All SNPs with a mapping quality ≤20.0 and a missing rate of >80% were excluded using VCFtools v0.1.13 (Danecek et al., 2011).

For resequencing data, variants were called using the Genotyper module in Sentieon Genomics software (version Sentieon-genomics-201911). Hard filtering of SNPs was performed using GenomeAnalysisTK v3.8 with the following criteria: QD < 2.0, FS > 60.0, RMSMappingQuality (MQ) < 40.0, MQRankSum < −12.5, ReadPosRankSum < −8.0, and StrandOddsRatio (SOR) > 3.0. After initial filtering, 94 854 916 SNPs remained. Subsequent filtering retained 654 343 SNPs that had less than 20% missing calls, a minimum quality score (minQ) greater than 30, and a minor allele frequency exceeding 0.05. Intergenic regions accounted for 51.54% of the SNPs, and 4.32% and 19.38% were located in exons and introns, respectively.

To evaluate the accuracy of SNP identification and genotyping, we selected 15 pairs of individuals from the RAD-seq and resequencing datasets across multiple populations. Hard filtering of SNP calls was performed using GenomeAnalysisTK v3.8 with the following criteria: QD < 2.0, FS > 60.0, MQ < 40.0, MQRankSum < −12.5, ReadPosRankSum < −8.0, and SOR > 3.0. Summary statistics were generated using SnpEff v4.3t (Cingolani et al., 2012), and SNP density per chromosome was calculated in a 1-Mb sliding window using VCFtools v0.1.17 (Danecek et al., 2011). In the RAD-seq dataset, 48.77% of SNPs were located in intergenic regions, 9.15% in exons, and 18.86% in introns (Supplemental Table 24). Similarly, in the resequencing dataset, 48.71% of SNPs were found in intergenic regions, 7.19% in exons, and 20.52% in introns (Supplemental Table 25). Both datasets demonstrated a strong positive correlation in genomic distribution and variation density (Supplemental Figures 39 and 40).

### Phylogenetic analysis

We performed a genome-wide phylogenetic analysis of *D. huoshanense* and *D. catenatum*, using *D. chrysotoxum* and *F.* *albopurpurea* as outgroups. Maximum-likelihood (ML)-based inference of the evolutionary tree was performed using RAxML (RAxML-VI-HPC) on the CIPRES Portal 2.0 (http://www.phylo.org/portal2/home.action) with default settings. To prevent mutual interference between *D. huoshanense* and *D. catenatum*, we generated an ML tree specifically for *D. catenatum*. All phylogenetic trees were visualized using FigTree v1.4.3 (http://tree.bio.ed.ac.uk/). We used the neighbor-net algorithm in SplitsTree6 v6.4.7 with default settings (Huson and Bryant 2024) on the population genomic data of *D. catenatum* and *D. huoshanense*. SplitsTree6 provides the Delta score and *Q*-residuals for each network. These statistics evaluate and delineate the network’s reticulation level and its “tree-like” properties. For resequencing data, we constructed an ML phylogenetic tree using FastTree v2.1.11 (Price et al., 2009) with default parameters. The phylogenetic analysis revealed that *D. huoshanense* and *D. catenatum* formed distinct lineages, with *D. catenatum* populations clustered according to their geographic locations (Supplemental Figure 41).

### Population structure and principal-component analysis (PCA)

We used ADMIXTURE v1.3.0 (Alexander et al., 2009) with cluster numbers (*K*) ranging from 1 to 4 to estimate the ancestry of the *D. huoshanense* and *D. catenatum* populations. In addition, we selected the F_1_ generation of artificial hybrid specimens as proxies for the hybrid population, from which the admixture proportion was estimated to be 50%. We performed a principal-component analysis with GCTA64 (Yang et al., 2011) using this same set of SNPs and recorded the first three components.

### Linkage disequilibrium, nucleotide diversity, and *F*_ST_ calculation

The linkage disequilibrium (r^2^) of all populations of *D. huoshanense* and *D. catenatum* was estimated for all biallelic SNPs using PopLDdecay v3.40 (Zhang et al., 2019) with the parameter “-MaxDist” set to 5 and “-OutType” set to 2. To minimize biases related to sample size, we randomly selected five individuals from each population. The nucleotide diversity (*π*), observed heterozygosity (*H*_*O*_), and expected heterozygosity (*H*_*E*_) of the *D. huoshanense* and *D. catenatum* populations, as well as the differentiation (*F*_ST_) between these two species, were calculated using Arlequin v3.5.2.2 (Excoffier and Lischer, 2010). Tajima’s *D* for *D. huoshanense* and the *D. catenatum* ecotype was computed using VCFtools v0.1.13 (Danecek et al., 2011) with a 10-kb non-overlapping window; any windows smaller than 10 kb were excluded from the analysis.

For resequencing data, we estimated genome-wide linkage disequilibrium (LD) decay (r^2^) between pairwise loci using PopLDdecay (Zhang et al., 2019). The LD-decay analysis revealed significant differences among populations of *D. huoshanense* and *D. catenatum*. The LD decay reached half of the maximum average r^2^ at approximately 21.5 kb in TP_YNGN and as little as 0.3 kb in HS_JXLHS, reflecting distinct demographic histories between the *D. huoshanense* and *D. catenatum* populations (Supplemental Figure 42). Notably, the LD decay observed in resequencing data was greater than that observed in RAD-seq data. In addition, nucleotide diversity (averaged across loci) (*π*), observed heterozygosity (*H*_O_), and expected heterozygosity (*H*_*E*_) were calculated for *D. huoshanense* and *D. catenatum* populations, along with inter- and intra-specific differentiation (*F*_ST_), using Arlequin v3.5.2.2 (Excoffier and Lischer, 2010). To compare genetic diversity across genomic regions, we partitioned the genome into coding and genomic-background regions for further analysis. Among *D. catenatum* populations, TP_YNGN had the highest nucleotide diversity (*π* = 0.36), whereas TP_JXLHS had the lowest (*π* = 0.27). Similar diversity patterns were observed in the coding regions (Supplemental Table 26). High genetic differentiation (*F*_ST_ ≥ 0.25) was observed between *D. huoshanense* and *D. catenatum*, and moderate differentiation (0.05 < *F*_ST_ < 0.15) was detected among the three *D. catenatum* populations (Supplemental Table 27). Nucleotide diversity estimated from resequencing data was higher than that obtained from RAD-seq data, and resequencing data revealed lower interspecies differentiation but higher intraspecies differentiation than RAD-seq data.

### Analysis of population phenotypic traits

Twelve phenotypic traits were measured in 414 individuals of *D. catenatum* and 355 individuals of *D. huoshanense*: stem length, stem diameter, length-to-diameter ratio of the stem, internode length, number of internodes, dorsal sepal length, dorsal sepal width, length-to-width ratio of the dorsal sepal, petal length, petal width, length-to-width ratio of the petal, and pedicel length. The samples were measured while growing under the common-garden conditions of the *Dendrobium* planting base in Huoshan County, Anhui Province, East China. Principal-component analyses were performed using the ggplot2 package (https://cran.r-project.org/web/packages/ggplot2/index.html) in R v4.0.5. Significant differences between the two species were assessed by one-way ANOVA, followed by the Tamhane test, performed with SPSS v19.0.

### Ecological niche modeling and dispersal corridors

We used ENM to predict the distribution of *D. catenatum* during four periods: the present, the mid-Holocene, the LGM (0.021–0.018 mya), and the LIG (0.14–0.12 mya). The ecological data used to perform ENM were derived from environmental variables extracted from the WorldClim database (http://www.worldclim.org/) (Hijmans et al., 2005). This dataset includes 19 bioclimatic variables and records that cover nearly the entire distribution range of native *D. catenatum* populations. Distribution predictions for the LGM and the present were generated using the CCSM4, MIROC-ESM, and MPI-ES-P models at a resolution of 2.5 arc minutes, whereas the prediction for the LIG was performed at a resolution of 30 arc seconds. To prevent model overfitting, we minimized the environmental variables. We selected five variables with pairwise Pearson correlation coefficients (*r* < 0.8) that made the most significant contributions to the niche models for subsequent analysis.

On the basis of the occurrence sites of *D. catenatum* and five environmental variables (bio2, bio10, bio15, bio17, and bio19), we constructed ecological niche models using current data and then projected these models for three additional time periods using maximum entropy with the default settings of Maxent v3.3.3 (Phillips et al., 2006). The predictive power of each model in the calibration region was assessed by comparing the model outputs, using 25% of the species records for testing and 75% for training the model.

To identify the dispersal corridors of *D. catenatum*, we integrated the distribution predictions from the LGM with the least-cost path method. This approach enabled us to predict the dispersal routes on the basis of publicly available chloroplast haplotype data (Hou et al., 2017) using ArcGIS v10.2.

### ABBA–BABA test at the ecotype, population, and chromosome levels

We used Patterson’s *D* statistic (Martin et al., 2015) to examine introgression between *D. huoshanense* and the three ecotypes of *D. catenatum*. Defining *F. albopurpurea* as the outgroup (O), we assessed the *D* statistics of the tree structure (((P1, P2), P3), O), where P3, P2, and P1 represent *D. huoshanense*, the eastern ecotype of *D. catenatum*, and the western ecotype of *D. catenatum*, respectively. The numbers of ABBA and BABA patterns in each block were calculated using the default parameters of ANGSD v0.918 (Korneliussen et al., 2014). To address the issue of non-independence within the sequence, we used a block-jackknifing procedure to assess statistical significance. To eliminate the possibility that the choice of outgroup (*F. albopurpurea*) influenced the *D* statistic, we repeated the tests using *D. chrysotoxum* as the outgroup. We also calculated Patterson’s *D* statistic to evaluate introgression between individual populations of *D. huoshanense* and *D. catenatum*. To further investigate the introgression between *D. huoshanense* (HS) and the eastern ecotype of *D. catenatum* (TP_E) across all 19 chromosomes, we used Patterson’s *D* statistic as described previously. We tested for excess allele sharing for each value of “-rf” set to the regions of the 19 chromosomes to evaluate Patterson’s *D* statistic. A *Z* value greater than 3 was considered a significant indicator of introgression.

### Inference of gene-flow direction

We analyzed the genomic sequencing data from *D. huoshanense* (HS) and the eastern ecotype (TP_E) of *D. catenatum*, extracting 205 coding and 225 non-coding loci on chromosome 1, following the methodology of Thawornwattana et al. (2022). For every locus, we derived one unphased diploid sequence from each species. We analyzed each heterozygous unphased diploid sequence as a resolved haplotype using BPP v4.8.4 (Flouri et al., 2018). We evaluated two MSC-I models that represented opposing directions of introgression: model I (HS to TP_E introgression) and model O (TP_E to HS introgression). We set the following priors: *τ* ∼ gamma (1, 10), *θ* ∼ gamma (2, 76), and *φ* ∼ beta (6, 200). We performed 10 000 iterations for burn-in and then recorded 100 000 samples, capturing one every 15 iterations. Analyzing one replicate dataset required roughly 24 h, given that *L* = 225 loci were processed with 20 threads. To ensure consistency across runs, we executed 10 independent runs for each model. We aggregated the obtained Markov chain Monte Carlo (MCMC) samples to produce the final posterior estimates.

We computed Bayes factors through thermodynamic integration with Gaussian quadrature, using the method described by Rannala and Yang (2017). The BFdriver program processed our control file to produce 16 control files with various beta values. These files facilitated the execution of BPP v4.8.4, enabling sampling from distinct power posterior distributions. Following the program run, we transferred the ElnfX values to an Excel file and calculated the logarithm of the marginal likelihood by summing the expression “weights × ElnfX/2” across the 16 data points. The ratio of posterior probabilities for the two models was approximated as *P*_1_/*P*_2_ = exp(*M*_1_ − *M*_0_).

### Inference of migration with TreeMix

To investigate genetic divergence and hybridization between *D. huoshanense* and various ecotypes of *D. catenatum*, we constructed an ML tree using TreeMix v1.13 (Pickrell and Pritchard, 2012), accounting for LD by grouping sites in blocks of 1000 SNPs, with migration events (-m) set to 0. For this analysis, *F. albopurpurea* was designated as the root. Bootstrap replicates and standard errors were used to assess the significance of migration events and the confidence in the inferred tree topology. After construction of an ML tree of *D. huoshanense* and various ecotypes of *D. catenatum*, migration events were incorporated (-m) and iterated 10 times for each value of m (1–3) to assess convergence regarding the model’s likelihood with each added migration event. The inferred ML trees and corresponding residuals were visualized using the built-in R-script plotting functions in TreeMix v1.13 (Pickrell and Pritchard, 2012).

For resequencing data, to investigate genetic divergence and hybridization between *D. huoshanense* (HS_JXLHS) and three populations of *D. catenatum* (TP_JXLHS, TP_HBXN, and TP_YNGN), we performed a TreeMix analysis using TreeMix v1.12 (Pickrell and Pritchard, 2012), grouping genomic sites into blocks of 500 SNPs. *F. albopurpurea* was designated as the outgroup root. Bootstrap replicates and standard errors were used to assess the significance of migration events and the confidence in the tree topology. After generation of the initial ML tree, migration events (-m) were iteratively tested for values of m ranging from 1 to 3, with 10 replicates per value to ensure model convergence. The final ML trees and residual plots were visualized using the built-in R-script plotting functions in TreeMix v1.12 (Pickrell and Pritchard, 2012). To confirm or refute the gene-flow events inferred by TreeMix, we calculated *f*_3_ statistics (Reich et al., 2009) using TreeMix v1.12 (Pickrell and Pritchard, 2012), with standard errors estimated via a block jackknife approach using blocks of 500 SNPs (significance threshold: *Z* < −3). The TreeMix analysis identified one migration edge from HS_JXLHS to TP_HBXN (Supplemental Figure 43), indicating introgression from *D. huoshanense* into *D. catenatum*. Various *f*_3_-statistic combinations tested gene flow between HS_JXLHS and TP_HBXN and between HS_JXLHS and TP_JXLHS, yielding *Z* scores below −3.0 (Supplemental Table 28). These results are consistent with our previous findings of introgression from the distantly related local species *D. huoshanense* into *D. catenatum*.

### Estimation of the mutation rate

The mutation rate was calculated using the formula *T* = *K*_S_/2*r*, where *K*_S_ represents the synonymous substitutions per site, *r* denotes the rate of synonymous substitutions per site, and *T* indicates the divergence time. The *K*_S_ values of paralogs in the *Apostasia shenzhenica* genes were approximately 1. Similar *K*_S_ values were identified in 11 other orchids across all five orchid subfamilies, ranging from 0.7 to 1.1 (Cai et al., 2015; Zhang et al., 2016, 2017). The absolute age of the whole-genome duplication event was estimated to be nearly 74 million years (Zhang et al., 2017). This estimated date for the *A. shenzhenica* lineage coincides with the date estimated for *Phalaenopsis equestris* (Cai et al., 2015).

### Simulation of evolutionary scenarios

We modeled the demographic history and performed model selection using *fastsimcoal2* (Excoffier et al., 2013), which uses the composite-likelihood method to infer demographic parameters based on the site frequency spectrum. Using SNP data in Variant Call Format (VCF) from *EasySFS* (https://www.github.com/isaacovercast/easySFS), we generated an unfolded joint site frequency spectrum (SFS) of *D. huoshanense* and three ecotypes of *D. catenatum*. We used the SFS to simulate demographic histories across multiple models, assessing the fit of the simulations to the empirical SFS data. This analysis was performed assuming a mutation rate of 6.675 × 10^−8^ mutations per site per generation. This rate was inferred from our mutation rate estimation, along with a generation time of 10 years, which was based on the estimated lifespans of the two species in the wild.

We tested four demographic models that represented isolation with gene flow between the eastern lineage of *D. catenatum* (TP_E) and *D. huoshanense* (HS): (1) early gene flow, (2) different gene flow, (3) constant gene flow, and (4) recent gene flow. The models used two populations: the HS_JXLHS population and the TP_JXLHS population.

For each model, we performed 100 independent runs of *fastsimcoal2* (Excoffier et al., 2013), with 200 000 simulations for each likelihood estimation and 40 cycles of the likelihood-maximization algorithm. The best model was identified on the basis of the ML values. We compared the simulated results of the four models with the observed site frequency spectra to assess the fit of the best demographic model.

Once the optimal model had been identified, we performed a final parameter estimation using all-SNP blocks with 10 individuals per ecotype. The distribution and sampling ranges of the model parameters were as follows (distribution; range): effective population sizes (log-uniform distribution; 1000 −60 000 haploid individuals), migration rates (log-uniform distribution; 0.00001–0.01 individuals per generation), divergence time between the western ecotype of *D. catenatum* (TP_W) and the ancestors of the central (TP_M) and eastern (TP_E) ecotypes of *D. catenatum* (log-uniform distribution; 0.001–1 years), and divergence time between the eastern (TP_E) and central (TP_M) ecotypes of *D. catenatum* (log-uniform distribution; 0.001–1 years). Finally, we established the divergence between *D. catenatum* and *D. huoshanense* as 6.4 mya (Xu, 2015; Xiang et al., 2016) to calibrate the parameters into absolute values. In addition, we presented the isotopic temperature of the atmosphere over the past 40 000 years (Petit et al., 1999).

### Flowering phenology

For each species, the number of flowering and non-flowering populations was recorded across all 27 populations of *D. huoshanense* and *D. catenatum* under common-garden conditions from April 24, 2018, to May 24, 2018. A flowering population was defined as one in which at least one flowering individual was observed, whereas a non-flowering population was defined as one in which no flowering individuals were observed during this period.

### Estimation of introgression signals

We calculated *f*_*dM*_ using the form *D* (TP_W, TP_E, HS, J) to scan the whole genome in 10-kb non-overlapping sliding windows using a publicly available script (Martin et al., 2015). Considering the majority of TP_W in the arboreal habitat (TP_TREE), four populations belonging to the karst landform were excluded. Before scanning, we filtered the raw VCF file using the parameters minQual < 30, DP < 3, and skipIndels. Windows that contained fewer than five sites were excluded; the median number of SNPs per window was 1001. Only positive *f*_*dM*_ values indicate excess allele sharing between TP_E and HS.

### Population genetic characteristics across different genomic regions

We calculated and compared the genetic diversity of introgressed regions and the genomic background among different lineages using 10- and 100-kb non-overlapping sliding windows with a publicly available script (Martin et al., 2015). Considering the majority of TP_W in the arboreal habitat (TP_TREE), we excluded four populations belonging to the karst landform. Prior to scanning, we filtered the raw VCF file using the parameters minQual < 30, DP < 3, and skipIndels. For each window, we estimated several population genomic indicators: total between-species sequence divergence (*D*_xy_), between-species population differentiation (*F*_ST_), within-species polymorphism level or nucleotide diversity (*π*), and SNP density. The population recombination rate (*ρ*) of TP_E was measured with FastEPRR using 10-kb windows (Gao et al., 2016). Tajima’s *D* was calculated with VCFtools v0.1.13 (Danecek et al., 2011) using 10-kb non-overlapping windows, and any windows smaller than 10 kb were discarded.

Nucleotide diversity (*π*), observed heterozygosity (*H*_*O*_), and expected heterozygosity (*H*_*E*_) at the ecotype or population level were calculated using Arlequin v3.5.2.2 software (Excoffier and Lischer, 2010). These calculations were based on the SNPs found in the coding regions of the introgressed genes and the genomic-background genes, provided that the missing rate was lower than 10%. We calculated the ratios of nonsynonymous substitution rates to synonymous substitution rates (*K*a/*K*s) for five introgressed genes (*CDPK*, *HHP*, *PIF*, *FY*, and *BRI1*) in *D. catenatum* populations and one individual of *D. huoshanense* (Han et al., 2020) using KaKs_Calculator v2.0 (Wang et al., 2010) with the YN model.

### Identification of paralogs of introgressed genes in the *D. catenatum* genome

To identify the paralogs of the introgressed genes in *D. catenatum*, the amino acid sequences of the introgressed genes were used as queries for a local BLASTP search against the *D. catenatum* genome database, with an *E*-value threshold of <1.0E−05. KofamKOALA (https://www.genome.jp/tools/kofamkoala/) was used to confirm the functions of the candidate paralogs. Multiple amino acid sequence alignments were generated using the default parameters of MUSCLE in MEGA v5.2.2 (Tamura et al., 2011). An ML tree of paralogs was constructed by aligning the amino acid sequences of candidate paralogs using RAxML (RAxML-VI-HPC) at CIPRES Portal 2.0 (http://www.phylo.org/portal2/home.action) with default settings. We checked the paralogs using ORTHOFINDER v3.0.1b1 (Emms and Kelly, 2019) with the parameter (-S blast) to classify the orthogroups of proteins from four *Dendrobium* species genomes: *D. catenatum* (Zhang et al., 2016), *D. huoshanense* (Han et al., 2020), *Dendrobium nobile* (Xu et al., 2022), and *D. chrysotoxum* (Zhang et al., 2021).

### Expression levels of unidirectionally introgressed genes in different tissues of *D. catenatum*

Expression data from various tissues of *D. catenatum* were analyzed on the basis of a previous study (Zhang et al., 2016). Samples of individual organs (flower bud, gynandrium, labellum, sepal, root tip, root, leaf, stem, and pollen) were pooled prior to sequencing, using three or more different plants.

### Differential expression of *D. catenatum* under drought and temperature stress

Expression data from *D. catenatum* under drought and temperature stress were analyzed on the basis of previously published transcriptomic profiling performed on *D. catenatum* under drought conditions (Wan et al., 2018). For the drought-stress experiment, four individuals were grown under simulated drought conditions, with the volumetric water content of the base material decreasing to 0% (Day_R_0_1, Day_R_0_2, Day_R_0_3, and Day_R_0_4). In addition, four individuals were grown with the volumetric water content of the base material reduced to between 30% and 35% (Day_R_30-35_1, Day_R_30-35_2, Day_R_30-35_3, and Day_R_30-35_4). For the temperature-stress experiment, seven individuals were cultivated in a greenhouse at 22°C (Night_L_10-15_1, Night_L_10-15_2, Night_L_10-15_3, Night_L_10-15_4, Night_L_10-15_5, Night_L_10-15_6, and Night_L_10-15_7), and six individuals were grown at 28°C (Day_L_10-15_1, Day_L_10-15_2, Day_L_10-15_3, Day_L_10-15_4, Day_L_10-15_5, and Day_L_10-15_6). The DESeq2 R package was used to identify DEGs between groups (Anders and Huber, 2010). To quantify the extent of differential expression of the introgressed genes, we selected the empirical 5% quantiles from the adjusted *p*-value distributions corresponding to each tissue.

### Differential gene expression in *D. catenatum* under different Cd treatments

Transcriptome data available for Cd stress in *D. catenatum* were analyzed in accordance with previously published physiological and transcriptomic studies on the Cd-stress response (Jiang et al., 2020). Seedlings were transferred to Murashige and Skoog (MS) medium supplemented with 0 mg L^−1^ (control, CK) or 5 mg L^−1^ CdSO_4._ Roots of control (CK) and Cd-treated plants were harvested after 15 and 30 days of treatment.

FastQC was used to process raw FASTQ reads and eliminate low-quality sequences. Clean reads were mapped to the *D. catenatum* genome using HISAT2 v2.2.1 software. The DESeq2 R package was used to identify DEGs between groups. Genes were considered differentially expressed if they had an adjusted *p* value < 0.01 and |log2FoldChange| > 1, as determined by DESeq2 (Anders and Huber, 2010).

### Identification of introgressed alleles derived from *D. huoshanense*

To identify the potential introgressed alleles derived from *D. huoshanense*, the amino acid sequences of the introgressed genes were used as queries to perform BLAST (Protein–Protein BLAST 2.15.0+) searches against the *D. huoshanense* genome database, with an *E*-value threshold of <1.0E−40 (Han et al., 2020). KofamKOALA (https://www.genome.jp/tools/kofamkoala/) was used to confirm the functions of the candidate orthologs. The most appropriate ortholog, which exhibited the lowest *E* value and aligned with the KofamKOALA results for the corresponding introgressed gene, was selected as the introgressed allele derived from *D. huoshanense* (HS-derived).

### Differential expression of introgressed alleles derived from *D. huoshanense* under various Cd treatments

The transcriptome data described above for Cd stress in *D. catenatum* were reanalyzed. FastQC was used to process the raw FASTQ reads and eliminate low-quality sequences. The clean reads were mapped to the *D. huoshanense* genome (Han et al., 2020) using HISAT2 v2.2.1 software. The DESeq2 R package was used to identify DEGs between groups. Genes were considered differentially expressed if they had an adjusted *p* value < 0.01 and |log2FoldChange| > 1, as determined by DESeq2 (Anders and Huber, 2010). Genes that were mapped to the genomic reference of *D. huoshanense* were named according to their corresponding genes in the *D. huoshanense* genome.

### Identification of candidate genes under positive selection

Positive selection typically leads to reduced genetic diversity within populations and increased genetic differentiation between populations (Wu et al., 2014). The genetic differentiation index *F*_ST_ (Weir and Cockerham, 1984) and the average proportion of pairwise mismatches across all compared sequences, θ_π_ (Tajima, 1983), have been used extensively to detect selection (Wu et al., 2014). To identify selection signals potentially associated with local adaptation, we calculated the genome-wide distributions of *F*_ST_ values and θ_π_ ratios for both interspecies (HS and TP_E) and intraspecies (TP_E and TP_TREE) comparisons using non-overlapping sliding windows of 10 kb. This analysis was performed using a publicly available script (Martin et al., 2015). We applied a *Z* transformation to the *F*_ST_ values and a log_10_ transformation to the θ_π_ ratios, designating windows within the top 5% of *Z*(*F*_ST_) and log_10_(θ_π_ ratio) values as candidate outliers indicative of strong selection. All outlier windows were then assigned to their corresponding genes.

## Data and code availability

The raw RAD-seq and whole-genome resequencing data have been deposited in the China National Genomics Data Center (https://ngdc.cncb.ac.cn) under accession number CNP0008137.

## Funding

This work was supported by the Funds for the Forestry Peak Discipline Construction Project of Fujian Agriculture and Forestry University (grant no. 72202200205).

## Acknowledgments

The authors declare no competing interests.

## Author contributions

Z.-J.L., Y.-B.L., S.L., and Z.J. conceived the project. Z.-J.L. and L.-J.C. collected and cultivated the plant materials. Z.R. and Z.J. prepared the samples. Z.J., Z.R., Z.-J.L., G.-Q.Z., and L.-J.C. conducted sequencing and processed the raw data. Z.J. and C.H. performed the introgression analysis. Z.R., G.-Q.Z., and X.M. carried out the transcriptome analysis. Z.J. and Z.R. analyzed the population genetic diversity. Z.J. and Z.-J.L. wrote the manuscript. Z.J., Z.R., X.M., C.H., Z.-J.L., Y.-B.L., S.L., D.-H.P., and G.W. revised the manuscript and provided valuable discussions.

## Supplemental information

Document S1. Supplemental Figures 1–43 and Supplemental Tables 2–10, 13–19, and 26–28

Supplemental Table 1. Overview of sample information and sequencing statistics

Supplemental Table 11. Sample size and genetic diversity of *D. huoshanense* and *D. catenatum* populations Ts/Tv, ratio of transition to transversion sites; RTSS, ratio of transition sites; RTVS, ratio of transversion sites; RPS, ratio of polymorphic sites; RPSS, ratio of private substitution sites.

Supplemental Table 12. Sample size and genetic diversity of *D. huoshanense* and *D. catenatum* populations in introgressed regions Ts/Tv, ratio of transition to transversion sites; RTSS, ratio of transition sites; RTVS, ratio of transversion sites; RPS, ratio of polymorphic sites; RPSS, ratio of private substitution sites.

Supplemental Table 20. Significantly enriched GO terms for biological processes associated with the 255 genes under selection between the eastern ecotype of *D. catenatum* (TP_E) and *D. huoshanense* (HS) populations, identified using θ_π_ ratios and Z(*F*_ST_) metrics

Supplemental Table 21. Significantly enriched KEGG pathways for the 255 selected genes differentiating the eastern ecotype of *D. catenatum* (TP_E) and *D. huoshanense* (HS) populations, identified using θ_π_ ratios and Z(*F*_ST_) metrics

Supplemental Table 22. Significantly enriched GO terms for biological processes associated with the 299 genes under selection between the eastern TP_E and western TP_TREE ecotypes of *D. catenatum* populations, identified using θ_π_ ratios and Z(*F*_ST_) metrics

Supplemental Table 23. Significantly enriched KEGG pathways for the 299 selected genes differentiating the eastern TP_E and western TP_TREE ecotypes of *D. catenatum* populations, identified using θ_π_ ratios and Z(*F*_ST_) metrics

Supplemental Table 24. Regional SNP distribution in 15 RAD-seq samples

Supplemental Table 25. Regional variation counts across 15 individual resequencing datasets

Document S2. Article plus supplemental information
