# Supplementary material for: Unidirectional genomic introgression facilitates the colonization of an invasive orchid in arid, metal-enriched sedimentary habitats
Source: Plant Commun. 2025 Oct 13;7(1):101561. doi: 10.1016/j.xplc.2025.101561 (PMC12902288; doi:10.1016/j.xplc.2025.101561)
Supplement: Document S1. Supplemental Figures 1–43 and Supplemental Tables 2–10, 13–19, and 26–28 [file mmc1.pdf]

Plant Communications, Volume 7

Supplemental information

Unidirectional genomic introgression facilitates the colonization of an invasive orchid in arid, metal-enriched sedimentary habitats

Zhenbin Jiao, Zhiyao Ren, Chao Hu, Xiaokai Ma, Guo-Qiang Zhang, Li-Jun Chen, Gang Wei, Dong-Hui Peng, Siren Lan, Yi-Bo Luo, and Zhong-Jian Liu

Supplemental information

Unidirectional genomic introgression facilitates the colonization of invasive orchid in arid metal-enriched sedimental habitats

Zhenbin Jiao[1,2,3,9], Zhiyao Ren[4,9], Chao Hu[2,3,5], Xiaokai Ma[1,6], Guo-Qiang Zhang[7], Li-Jun Chen[7], Gang Wei[8], Dong-Hui Peng[1], Siren Lan[1,*], Yi-Bo Luo[2,*], Zhong-Jian Liu[1,*]

Unidirectional genomic introgression facilitates the colonization of invasive orchid in arid metal-enriched sedimental habitats

Zhenbin Jiao[1,2,3,9], Zhiyao Ren[4,9], Chao Hu[2,3,5], Xiaokai Ma[1,6], Guo-Qiang Zhang[7], Li-Jun Chen[7], Gang Wei[8], Dong-Hui Peng[1], Siren Lan[1,*], Yi-Bo Luo[2,*], Zhong-Jian Liu[1,*]

[1] Key Laboratory of Orchid Conservation and Utilization of National Forestry and Grassland Administration at College of Landscape Architecture and Art, Fujian Agriculture and Forestry University, Fuzhou 350002, China

[2] State Key Laboratory of Systematic and Evolutionary Botany, Institute of Botany, Chinese Academy of Sciences, Beijing 100093, China

[3] University of Chinese Academy of Sciences, Beijing 100049, China

[4] Guangzhou Geriatric Hospital, Guangzhou 510180, China

[5] Eastern China Conservation Centre for Wild Endangered Plant Resources, Shanghai Chenshan Botanical Garden, Shanghai 201602, China

[6] Center for Genomics and Biotechnology, Haixia Institute of Science and Technology, School of Future Technology, Fujian Agriculture and Forestry University, Fuzhou 350002, China

[7] Shenzhen Key Laboratory for Orchid Conservation and Utilization and The National Orchid Conservation Center of China, The Orchid Conservation and Research Center of Shenzhen, Shenzhen 518114, China

[8] School of Pharmaceutical Sciences, Guangzhou University of Chinese Medicine, Guangzhou 510006, China

[9] These authors contributed equally to this article.

* Correspondence: Siren Lan (lkzx@fafu.edu.cn), Yi-Bo Luo (luoyb@ibcas.ac.cn), Zhong-Jian Liu (zjliu@fafu.edu.cn).

Short summary: Our study demonstrates unidirectional genomic introgression from *Dendrobium huoshanense* to the invasive species *D. catenatum* in lithophytic habitats. The introgressed regions contain genes that regulate responses to drought and metal ion stress, facilitating the colonization of *D. catenatum* in arid, metal-enriched sedimentary environments. These findings elucidate the evolutionary role of unidirectional introgression in the environmental adaptation of perennial herbaceous plants.

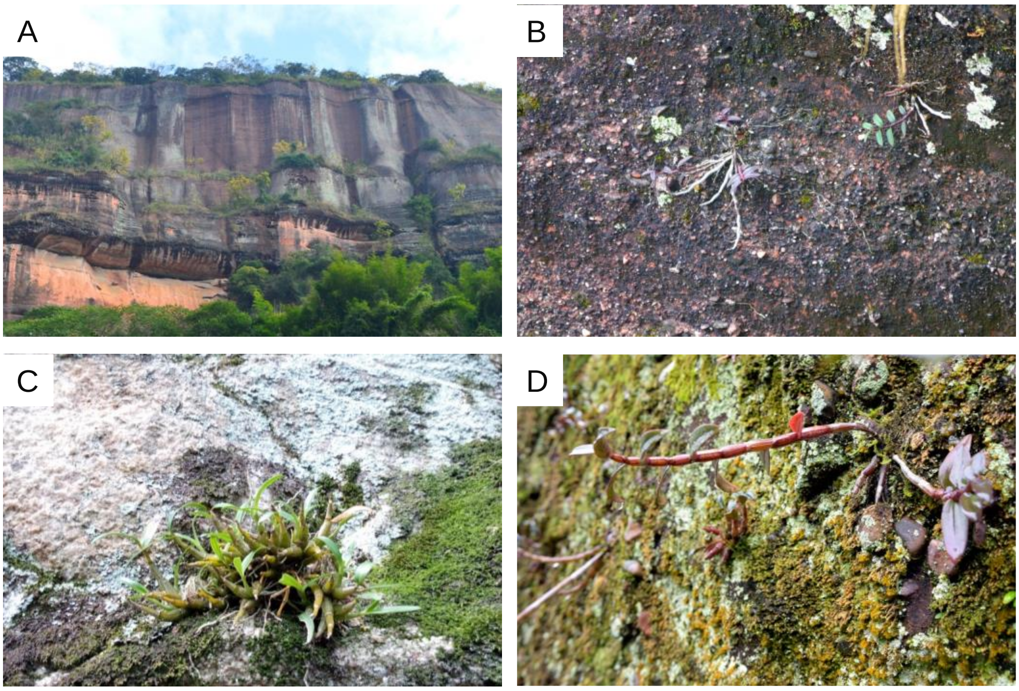

Supplemental Figure 1. Photographs of *Dendrobium catenatum* and *D. huoshanense*.

(A) A birds-eye view of the Danxia landform in Shaoguan, Guangdong Province (photo credit: Gang Wei).

(B) An image of the red sedimentary rock characteristic of the Danxia landform in Danxiashan Mountain, Guangdong Province (photo credit: Gang Wei).

(C) *D. huoshanense* on the cliff in Huoshan County, Anhui Province (HS_ANHS) (photo credit: Zhenbin Jiao).

(D) *D. catenatum* in Danxiashan Mountain, Guangdong Province (TP_GDSG) (photo credit: Gang Wei).

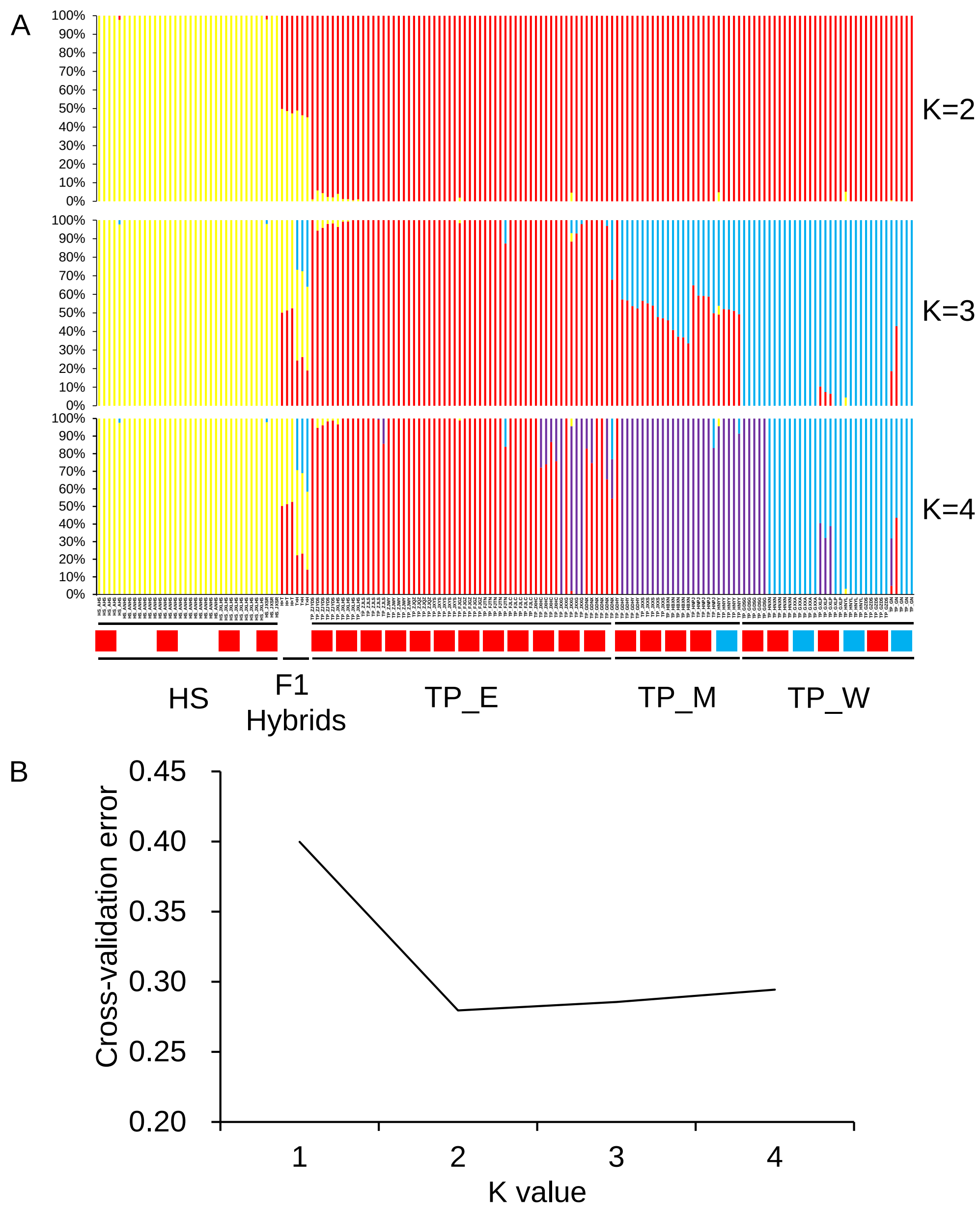

Supplemental Figure 2. The population structure of *Dendrobium catenatum* and *D. huoshanense* individuals was revealed through admixture analysis.

(A) The population structure inferred from an admixture analysis of *D. catenatum* and *D. huoshanense* individuals (*K* = 2 to 4) is presented, with red squares representing lithophytic habitats and green squares representing epiphytic habitats.

(B) Cross-validation errors for each *K* and those for *K* from 1 to 4 are shown in the panel.

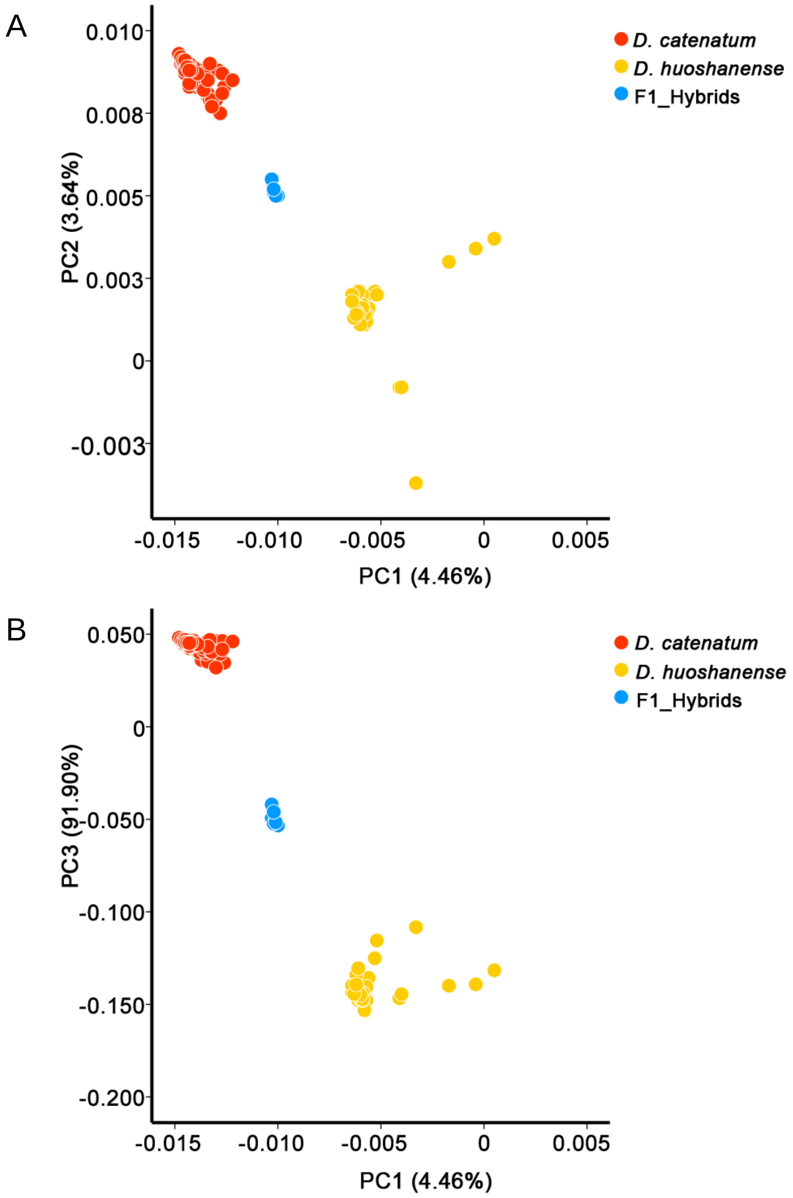

Supplemental Figure 3. PCA plots of SNP data for *Dendrobium catenatum* (red), *D. huoshanense* (yellow), and artificial F_1 hybrids (blue).

(A) PCA plots of PC1 and PC2.

(B) PCA plots of PC1 and PC3.

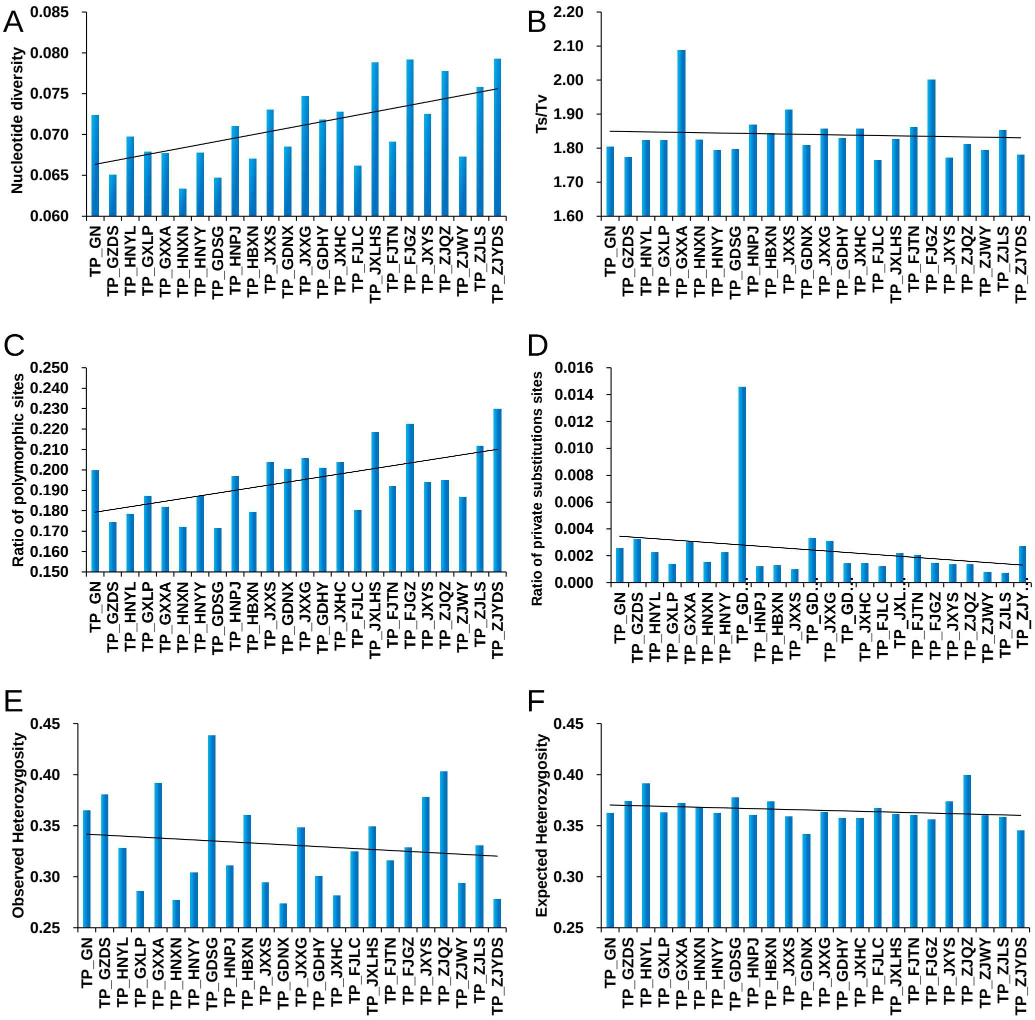

Supplemental Figure 4. Molecular diversity indices of *Dendrobium catenatum*.

(A) Nucleotide diversity.

(B) Transition-Transversion Ratio (Ts/Tv).

(C) Ratio of polymorphic sites.

(D) Ratio of private substitution sites.

(E) Observed heterozygosity.

(F) Expected heterozygosity.

Populations are displayed from western to eastern China.

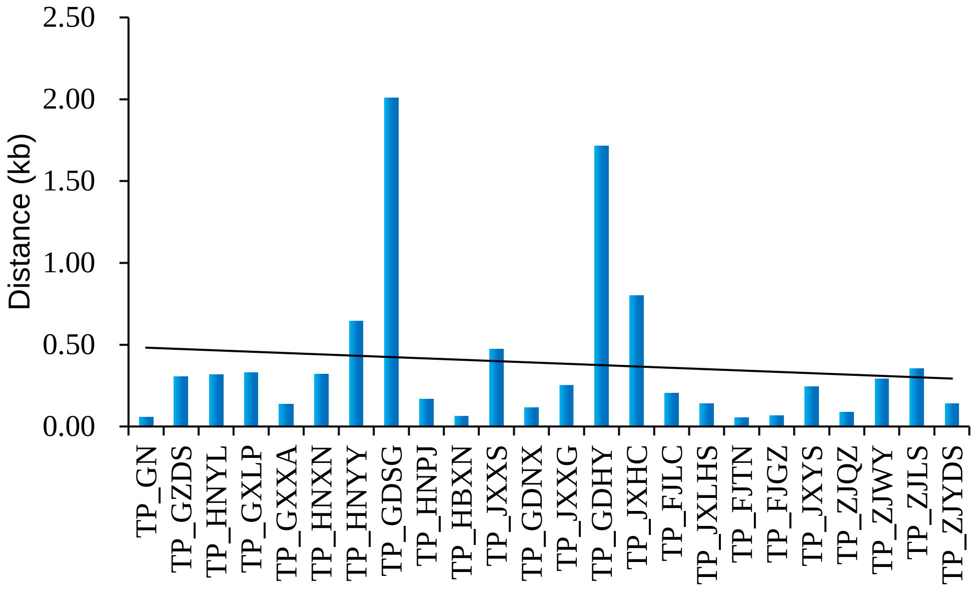

Supplemental Figure 5. Bar plots illustrating the decay distance of linkage disequilibrium in *Dendrobium catenatum* populations, based on whole-genome SNPs data.

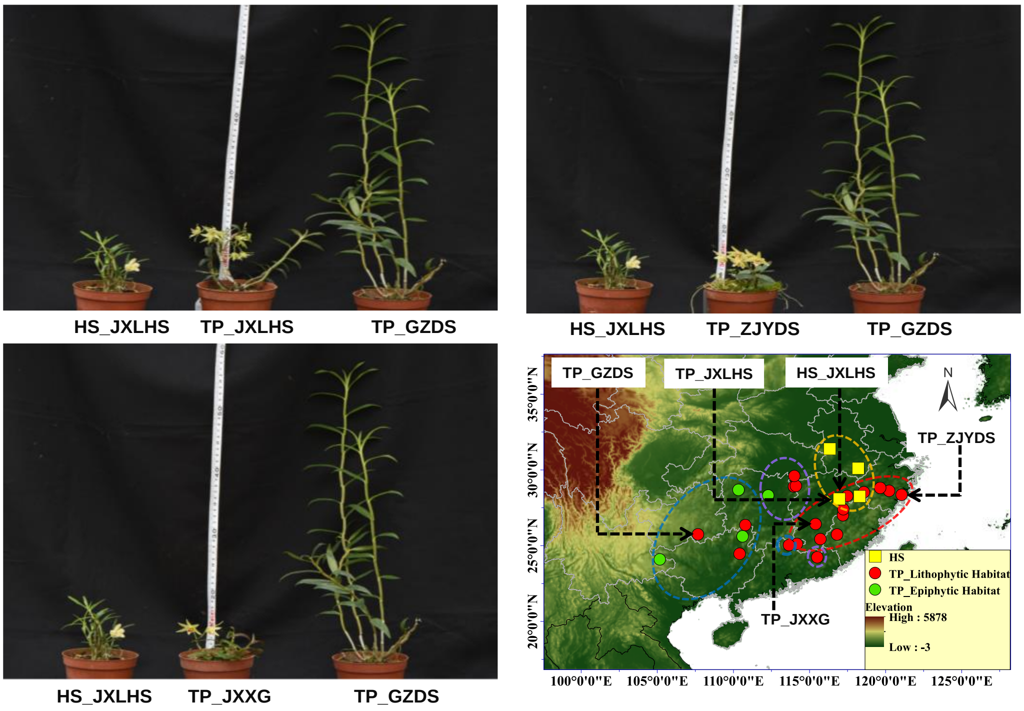

Supplemental Figure 6. Morphology characteristics of *Dendrobium huoshanense* and various populations of *D. catenatum* individuals.

D. huoshanense in Longhushan Mountain, Jiangxi Province (HS_JXLHS). *D. catenatum* in Longhushan Mountain, Jiangxi Province (TP_JXLHS). *D. catenatum* in Yandang Mountain, Zhejiang Province (TP_ZJYDS). *D. catenatum* in Xingguo County, Jiangxi Province (TP_JXXG). *D. catenatum* in Dushan County, Guizhou Province (TP_GZDS). Symbols correspond to samples of species as follows: circle represent *D. catenatum*, while square represent *D. huoshanense*. Each circular curve corresponds to a specific ecotype or species, indicated by the following colors: red for TP_E, purple for TP_M, blue for TP_W, and yellow for HS.

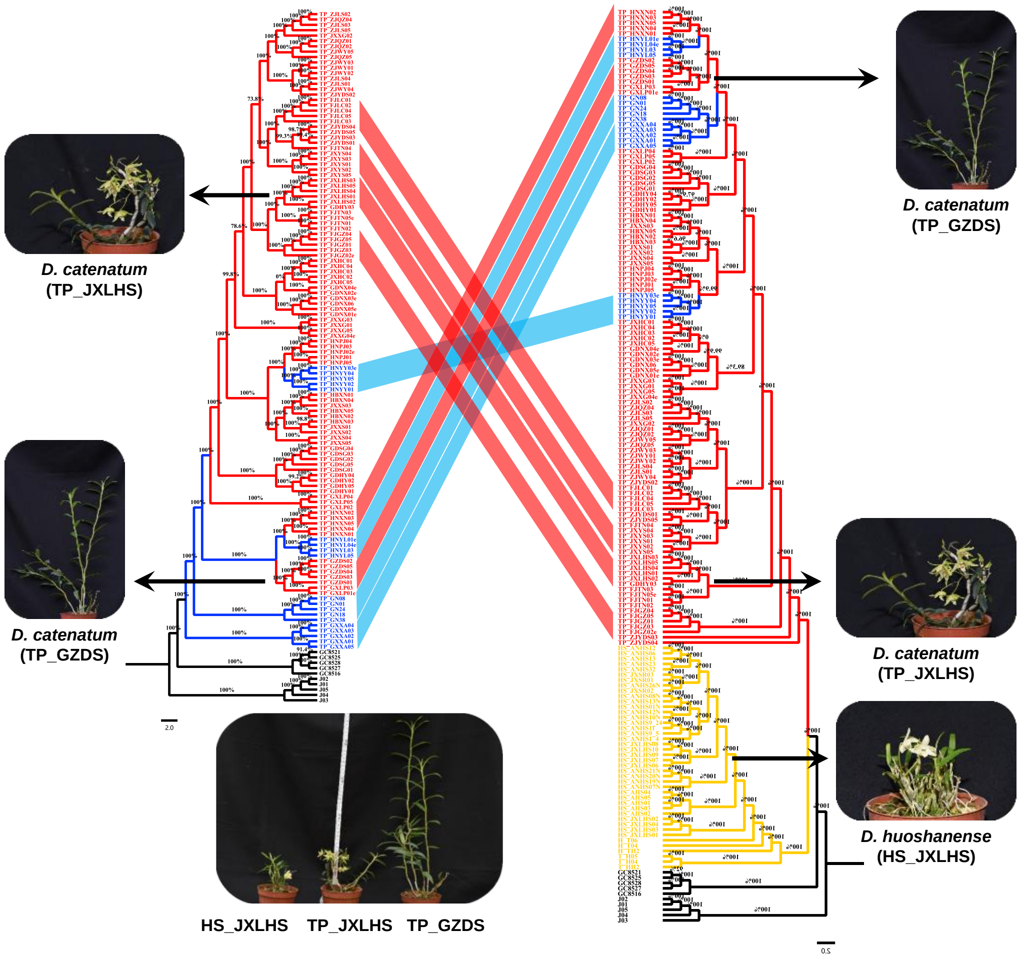

Supplemental Figure 7. Inconsistent genomic phylogenetic topologies among populations of *Dendrobium catenatum* and *D. huoshanense*.

A cladogram illustrating the relationships among various populations of *D. catenatum* and *D. huoshanense*, accompanied by representative photos of the phenotypic traits of each species. *D. chrysotoxum* and *Flickingeria albopurpurea* are outgroups. Colors correspond to a specific habitat as follows: red, lithophytic habitat; blue, epiphytic habitat. Photos: *D. huoshanense* in Longhushan Mountain, Jiangxi Province (HS_JXLHS). *D. catenatum* in Longhushan Mountain, Jiangxi Province (TP_JXLHS). *D. catenatum* in Dushan County, Guizhou Province (TP_GZDS).

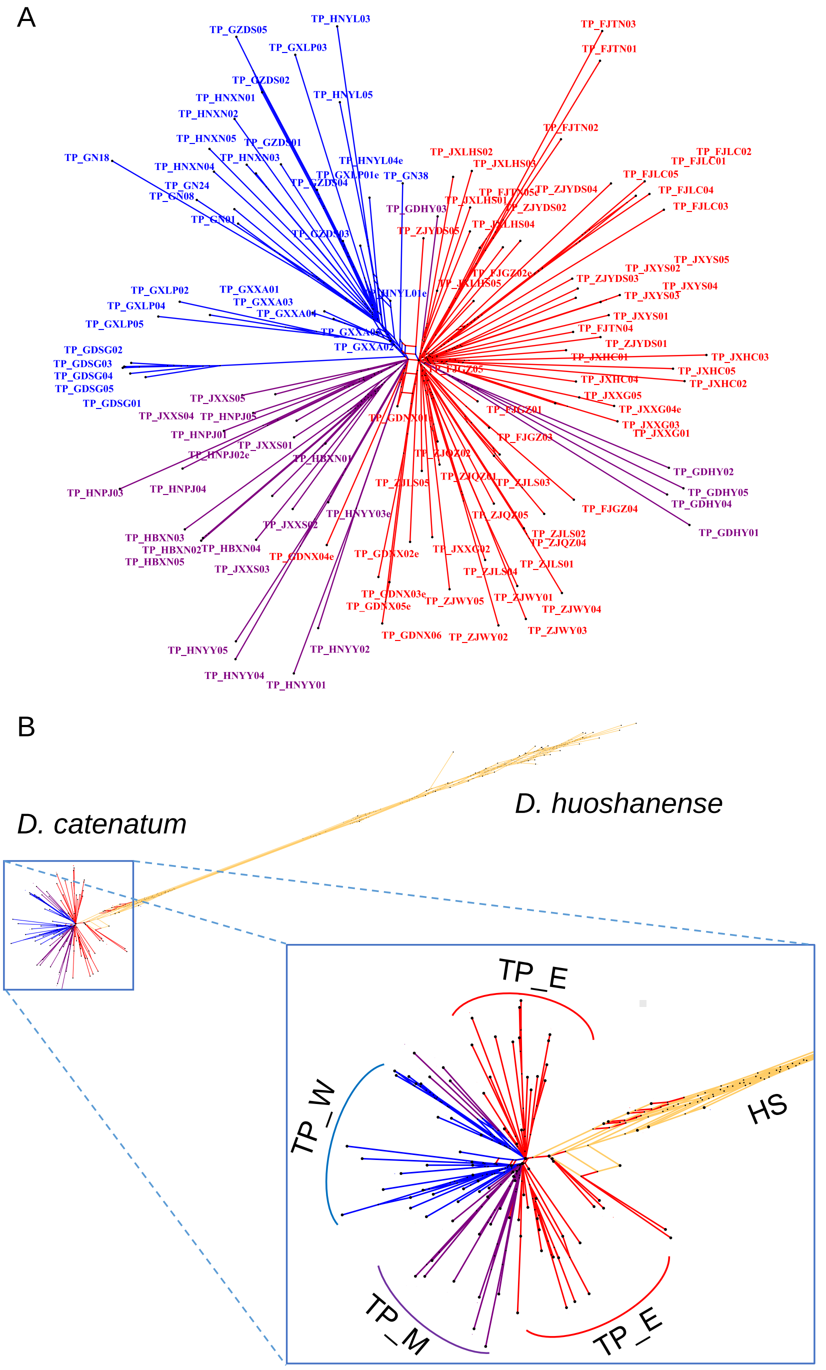

Supplemental Figure 8. Neighbor-net analysis of *D. catenatum* and *D. huoshanense* individuals at the population level.

(A) The neighbor-net analysis utilized population genomic data from *D. catenatum* populations. The neighbor-net for the *D. catenatum* population has a Delta score of 0.4095 and a *Q*-residual of 0.008005.

(B) The neighbor-net analysis was conducted using the population genomic databases of both *D. catenatum* and *D. huoshanense* populations. For the combined populations of *D. catenatum* and *D. huoshanense*, the neighbor-net yields a Delta score of 0.3555 and a *Q*-residual of 0.05933. Each color corresponds to a specific ecotype or species: red for TP_E, purple for TP_M, blue for TP_W, and yellow for HS.

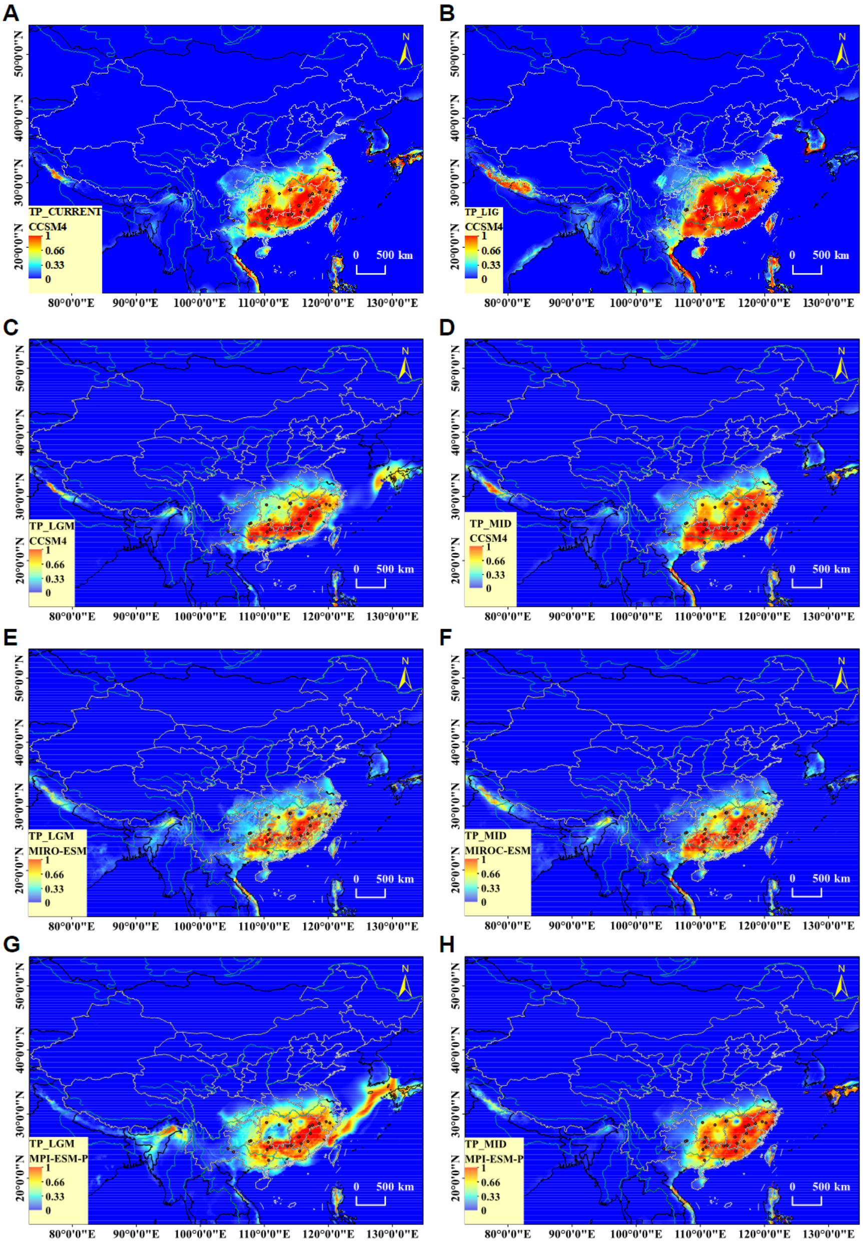

Supplemental Figure 9. Predicted distributions of *Dendrobium catenatum*.

(A) the present with CCSM4 model.

(B) the Last Interglacial (LIG) with CCSM4 model.

(C) the Last Glacial Maximum (LGM) with CCSM4 model.

(D) the mid-Holocene (MID) with CCSM4 model.

(E) the LGM with MIROC-ESM model.

(F) the MID with MIROC-ESM model.

(G) the LGM with MPI-ESM-P model.

(H) the MID with MPI-ESM-P model.

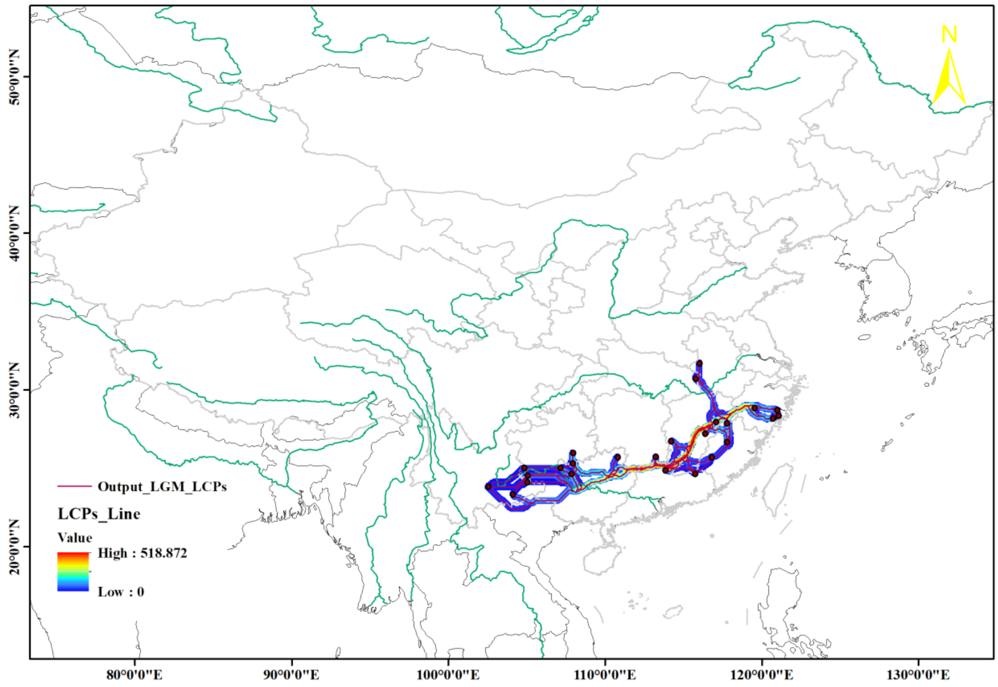

Supplemental Figure 10. Proposed expansion routes of *Dendrobium catenatum* from southwestern to eastern China.

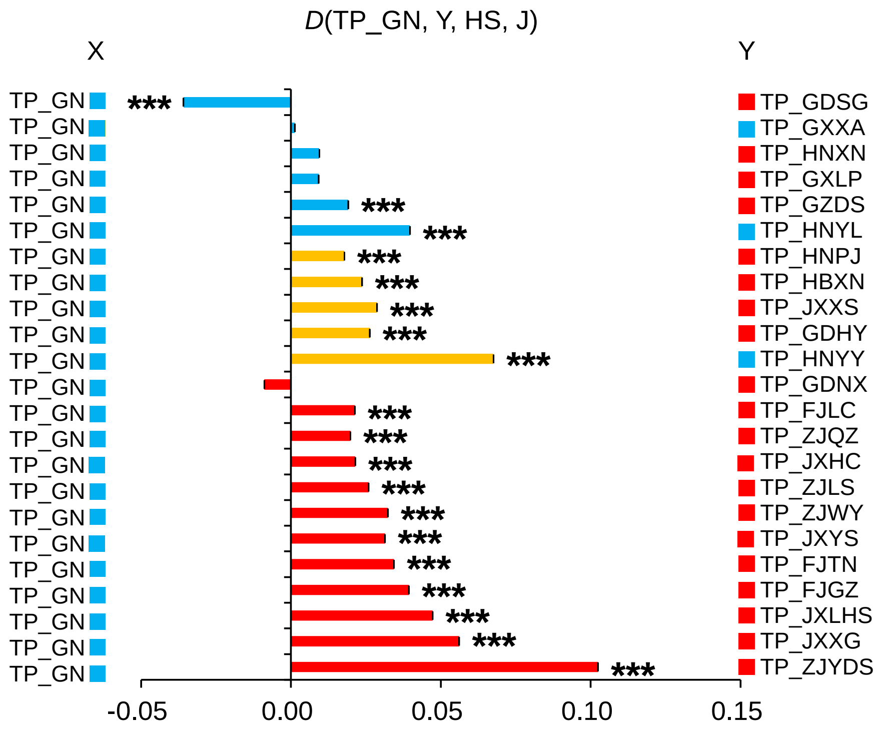

Supplemental Figure 11. *D*-statistics of potential gene flow between *Dendrobium huoshanense* (HS) and various ecotypes of *D. catenatum*.

Populations with red, yellow, and blue histograms correspond to the eastern, central, and western ecotypes, respectively. The *D* (X, Y, HS, J) value that significantly deviates from zero (i.e., greater than three times the standard error) is represented by the *Z* value. The red squares indicate lithophytic habitats, while the blue squares represent epiphytic habitats. Asterisks denote significant differences (*, $P < 0.05$; **, $P < 0.01$; ***, $P < 0.001$) based on a block jackknife approach.

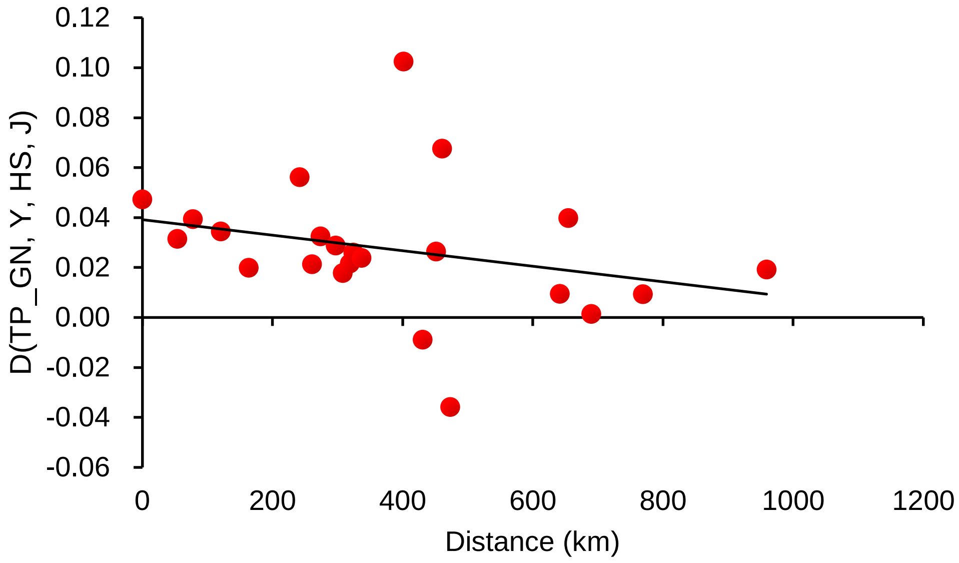

Supplemental Figure 12. Negative correlation trend between *D* (TP_GN, Y, HS, J) and the geographic distance.

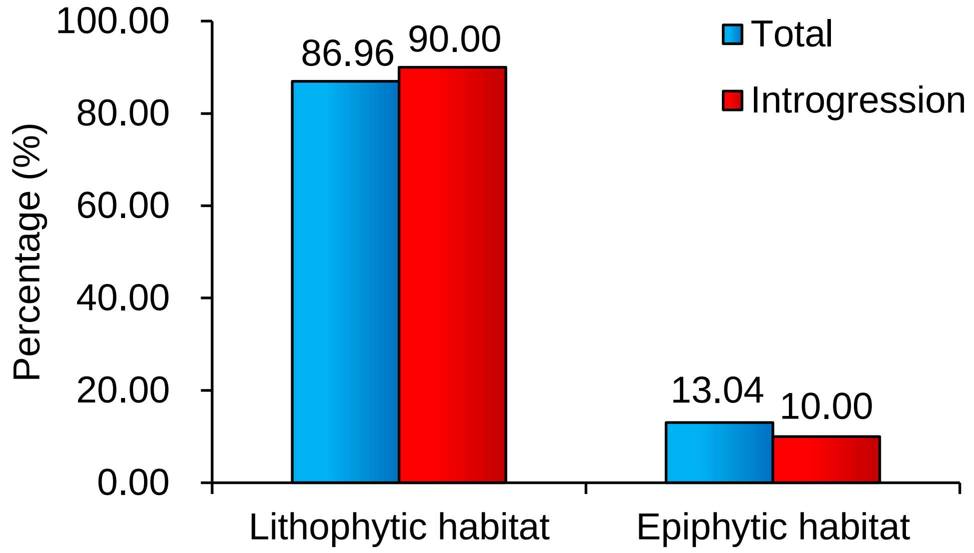

Supplemental Figure 13. Habitat type distribution of all and introgressed populations of *Dendrobium catenatum*.

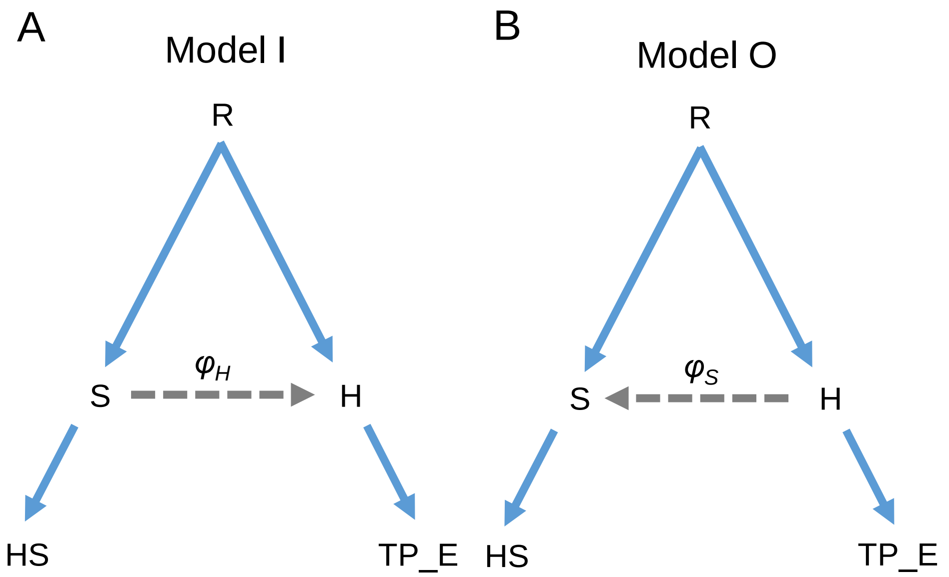

Supplemental Figure 14. MSC-I models for two species with different introgression directions in coding and non-coding regions of Chromosome 1.
(A) HS → TP_E introgression with $\Theta_I = (\theta_{HS}, \theta_{TP\_E}, \theta_R, \theta_S, \theta_H, \tau_R, \tau_S, \tau_H, \varphi_H)$, **(B)** TP_E → HS introgression with $\Theta_O = (\theta_{HS}, \theta_{TP\_E}, \theta_R, \theta_S, \theta_H, \tau_R, \tau_S, \tau_H, \varphi_S)$. The magnitude of introgression is measured by the introgression probability: φ_H in **(A)** or φ_S in **(B)**. Horizontal arrows (S-H and H-S) represent introgression events rather than real populations and have no θ associated with them. The arrow points to introgression direction in the real world (forward in time).

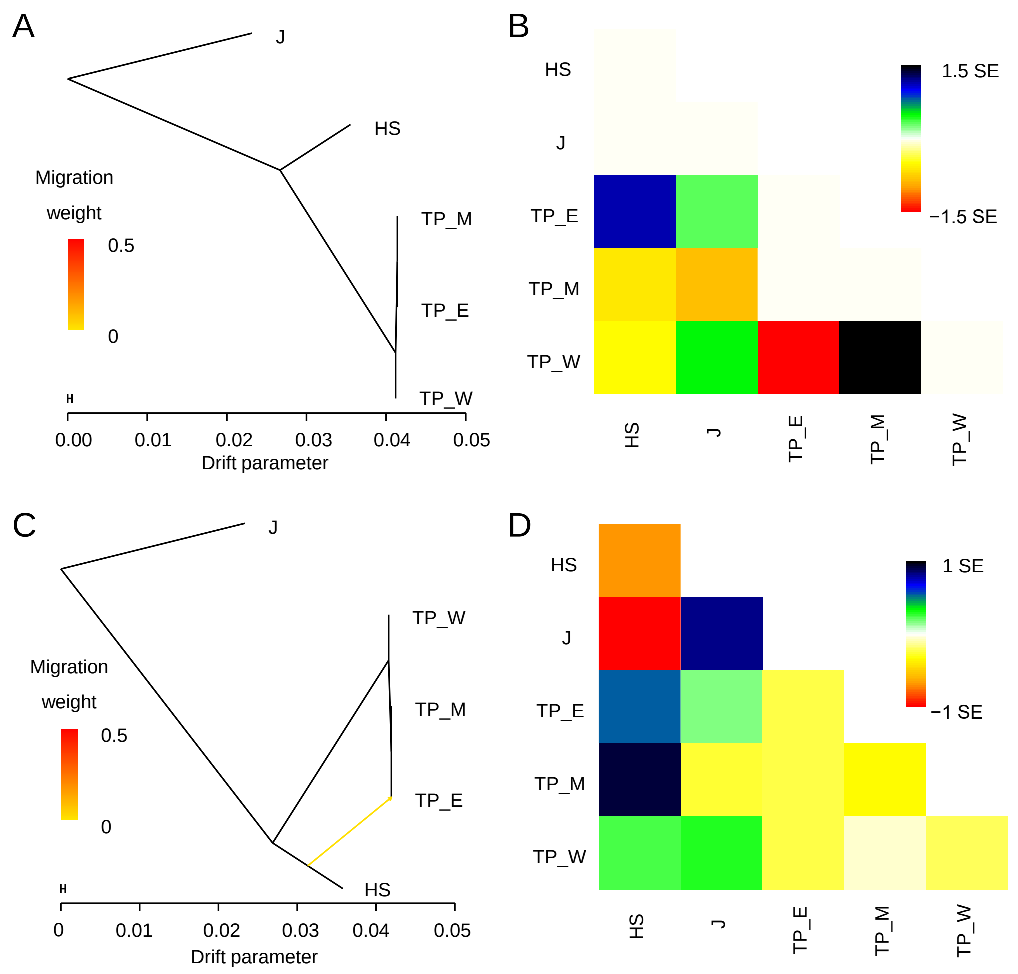

Supplemental Figure 15. Migration events between *Dendrobium huoshanense* and three ecotypes of *D. catenatum* were estimated using TreeMix.

(A) The maximum likelihood tree was inferred using TreeMix without any migration edges.

(B) The residuals of the model fit, which does not account for migration events, exhibit high positive standard error (SE) values.

(C) The maximum likelihood tree inferred using TreeMix, with one allowed migration edge, is illustrated with arrows that are colored according to the migration weight.

(D) The residuals of the model fit with a single migration event.

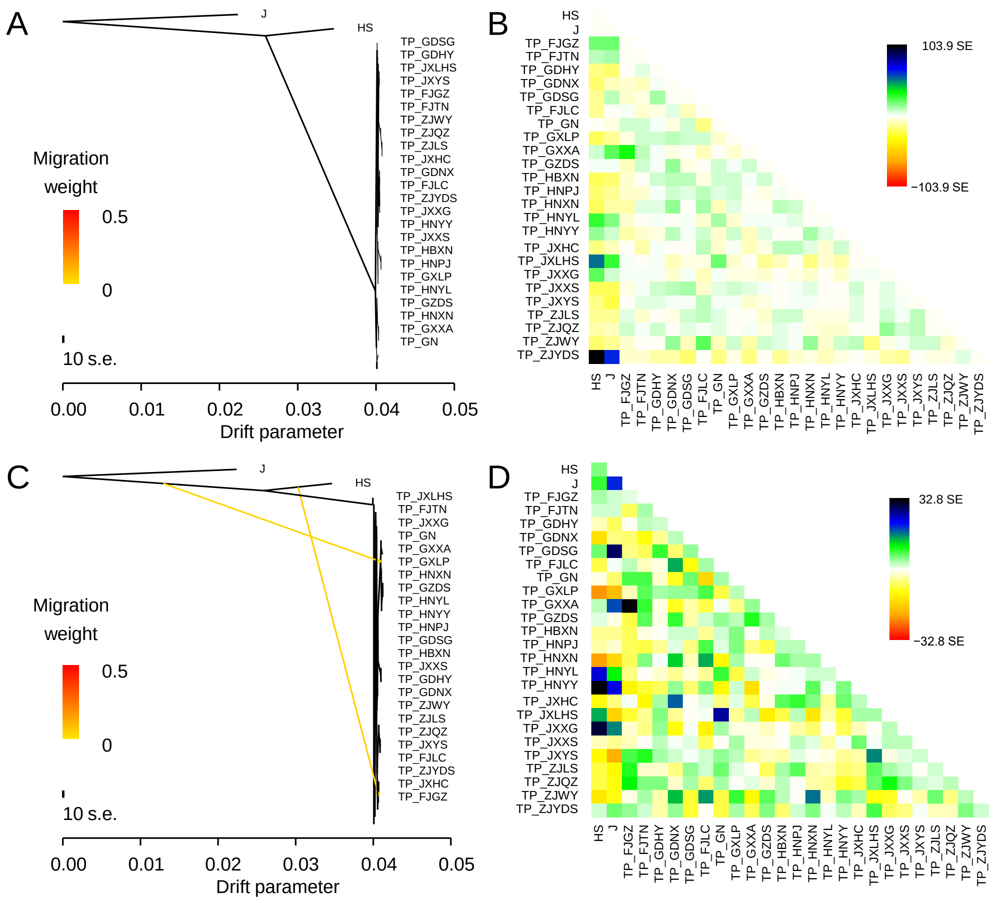

Supplemental Figure 16. Migration events between *Dendrobium huoshanense* and *D. catenatum* populations were estimated using TreeMix.

(A) The maximum likelihood tree was inferred using TreeMix without any migration edges.

(B) The residuals of the model fit, which does not account for migration events, exhibit high positive standard error (SE) values.

(C) The maximum likelihood tree inferred using TreeMix, with two allowed migration edges, is illustrated with arrows that are colored according to their migration weights.

(D) The residuals of the model fit with two migration events.

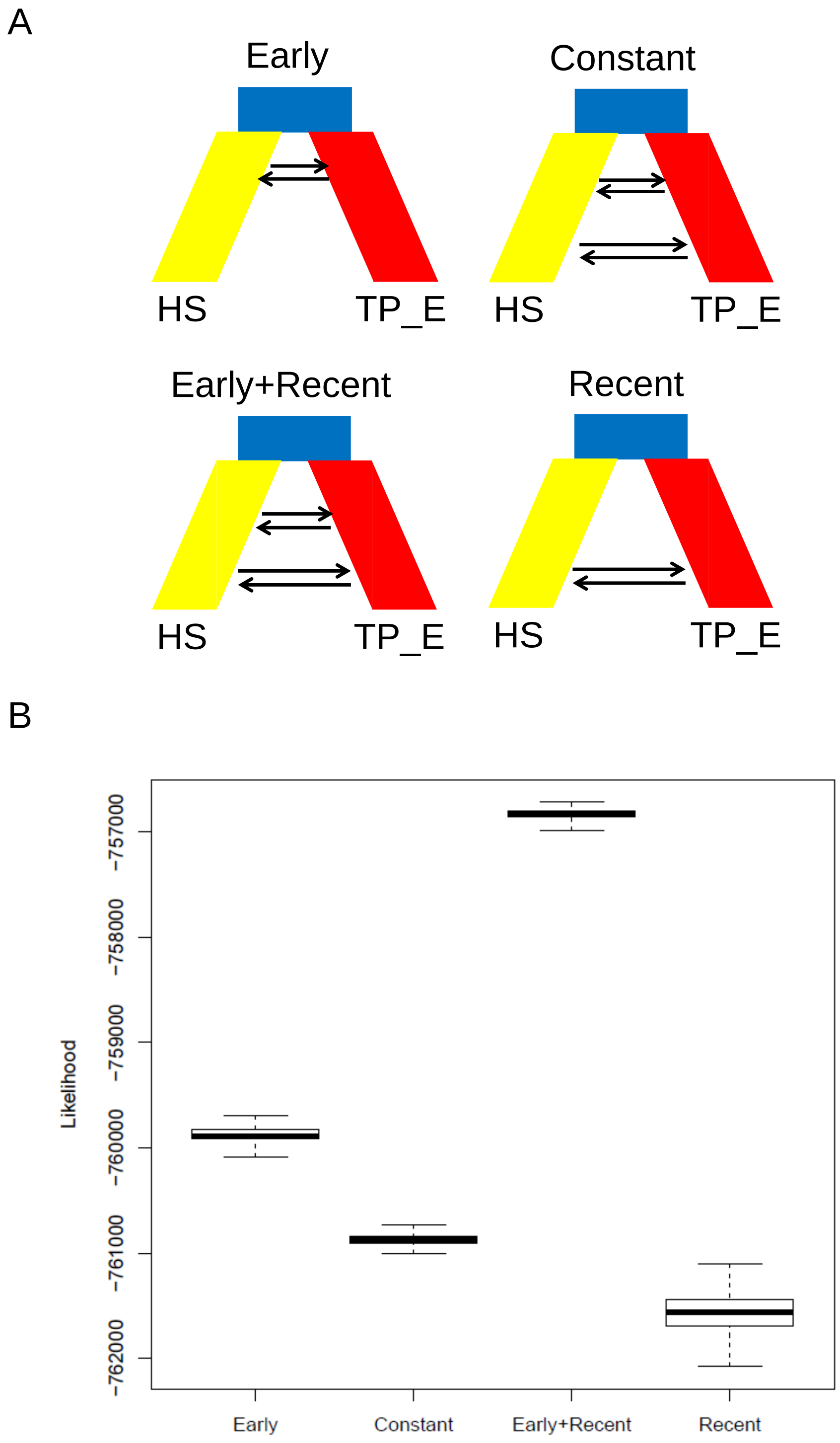

Supplemental Figure 17: Schematic representation and likelihood analysis of various alternative gene flow scenarios.

(A) Schematic representation of the different models tested to infer the gene flow between *D. catenatum* and *D. huoshanense*.

(B) Analysis of likelihood for various gene flow scenarios.

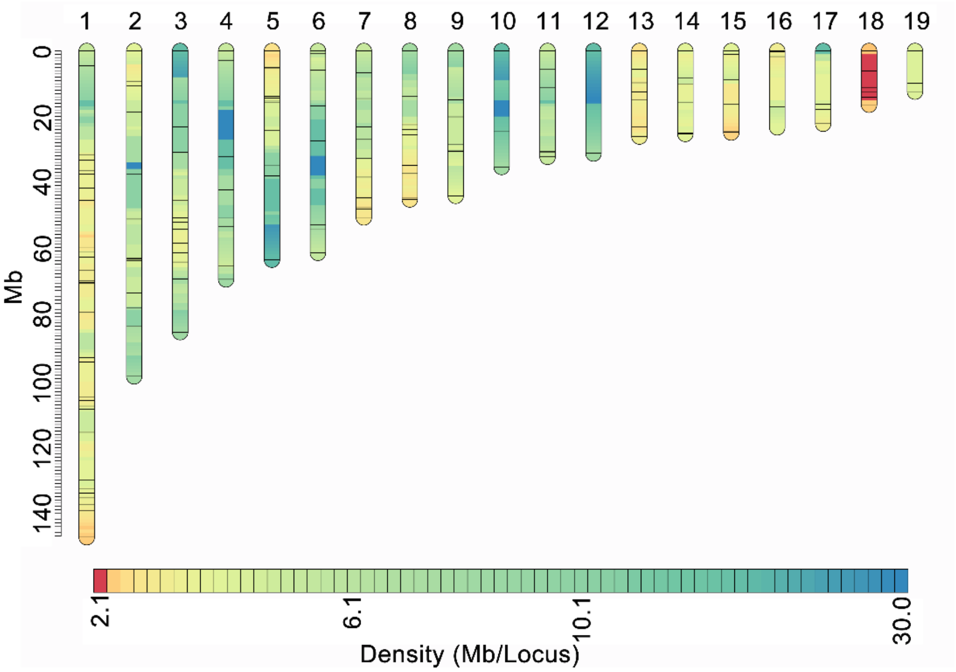

Supplemental Figure 18. Chromosomal positions and the density of introgressed alleles mapped onto the *D. catenatum* genome.

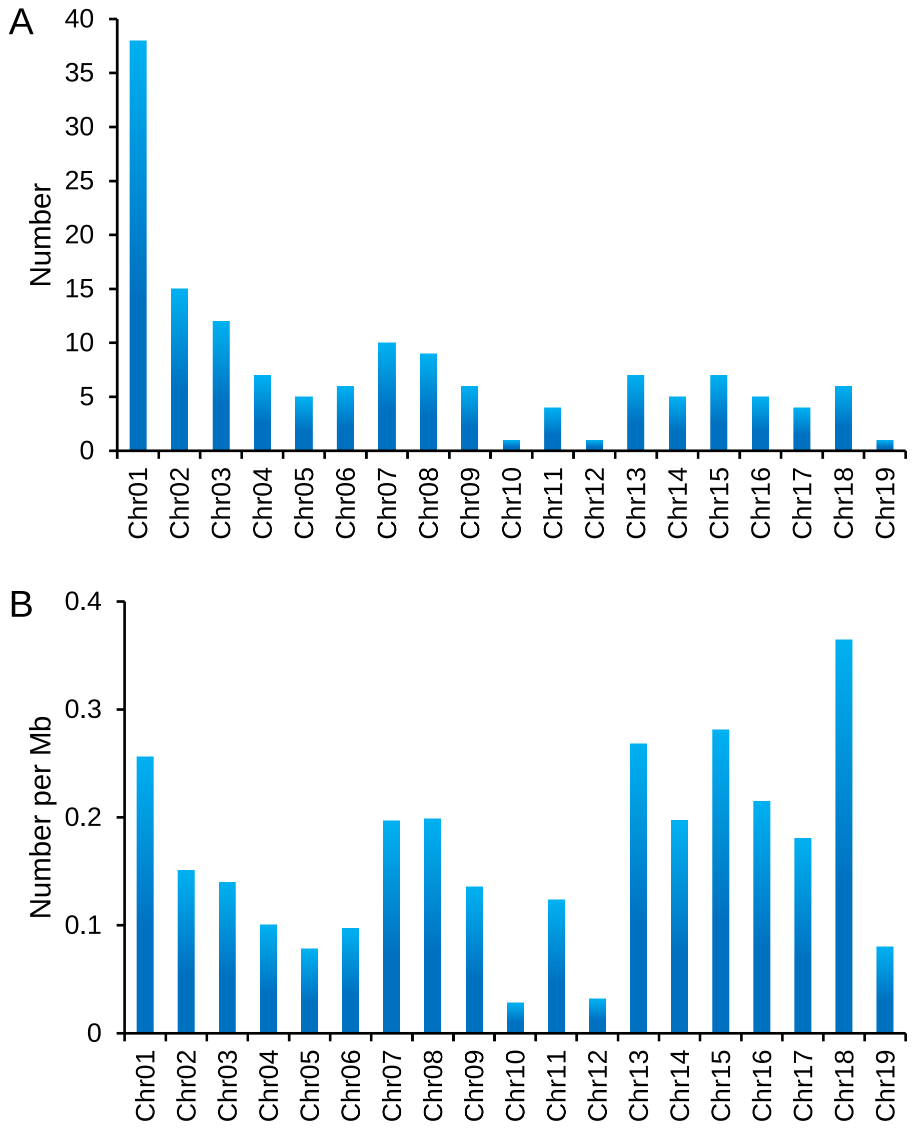

Supplemental Figure 19. Distribution patterns of introgressed genes in the *Dendrobium catenatum* genome.

(A) The distribution of introgressed genes across the 19 chromosomes of *D. catenatum*.

(B) The density of introgressed genes present in each of the 19 chromosomes of *D. catenatum*.

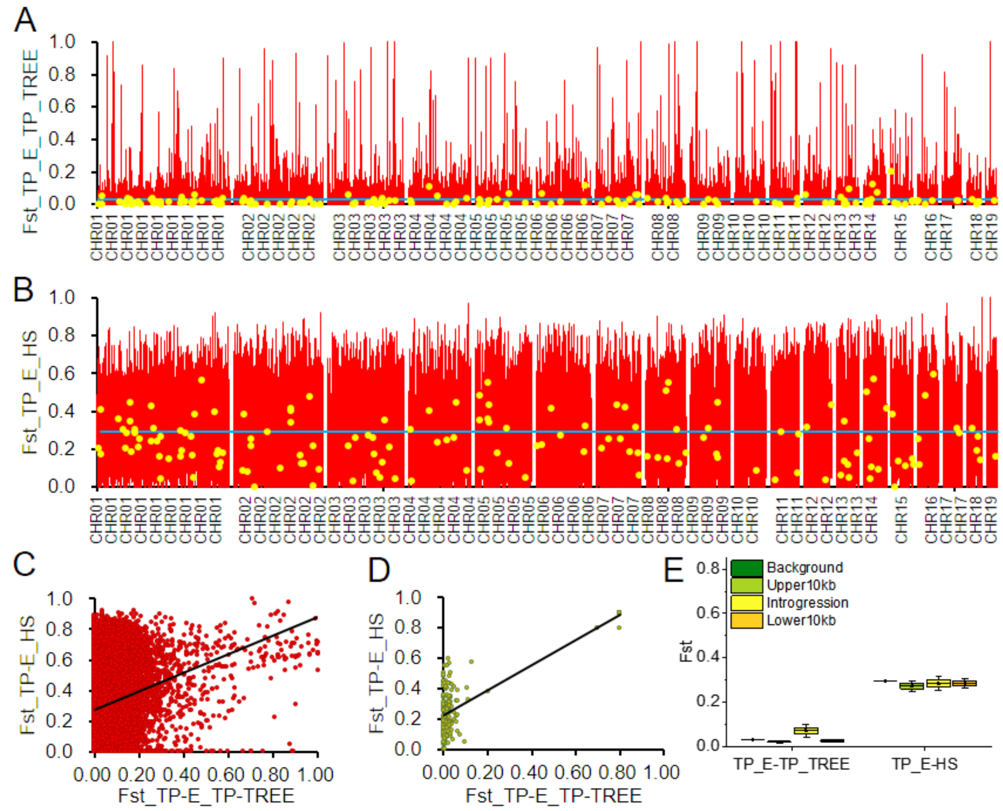

Supplemental Figure 20. Heterogeneity of genomic divergence (F_{ST}) among TP_E, TP_TREE and HS.

(A) Pairwise genetic divergence (F_{ST}) was analyzed in 10 kb sliding windows across 19 chromosomes between TP_TREE and TP_E.

(B) Pairwise genetic divergence (F_{ST}) was analyzed in 10 kb sliding windows across 19 chromosomes between TP_E and HS.

(C) Pairwise genetic divergence (F_{ST}) of TP_TREE and TP_E compared to HS and TP_E in the genome.

(D) Pairwise genetic divergence (F_{ST}) of TP_TREE and TP_E compared to HS and TP_E in introgressed regions.

(E) The boxplot illustrates the average pairwise genetic divergence (F_{ST}) across all comparisons within the genomic background, introgressed regions, and the upper and lower 10 kb regions.

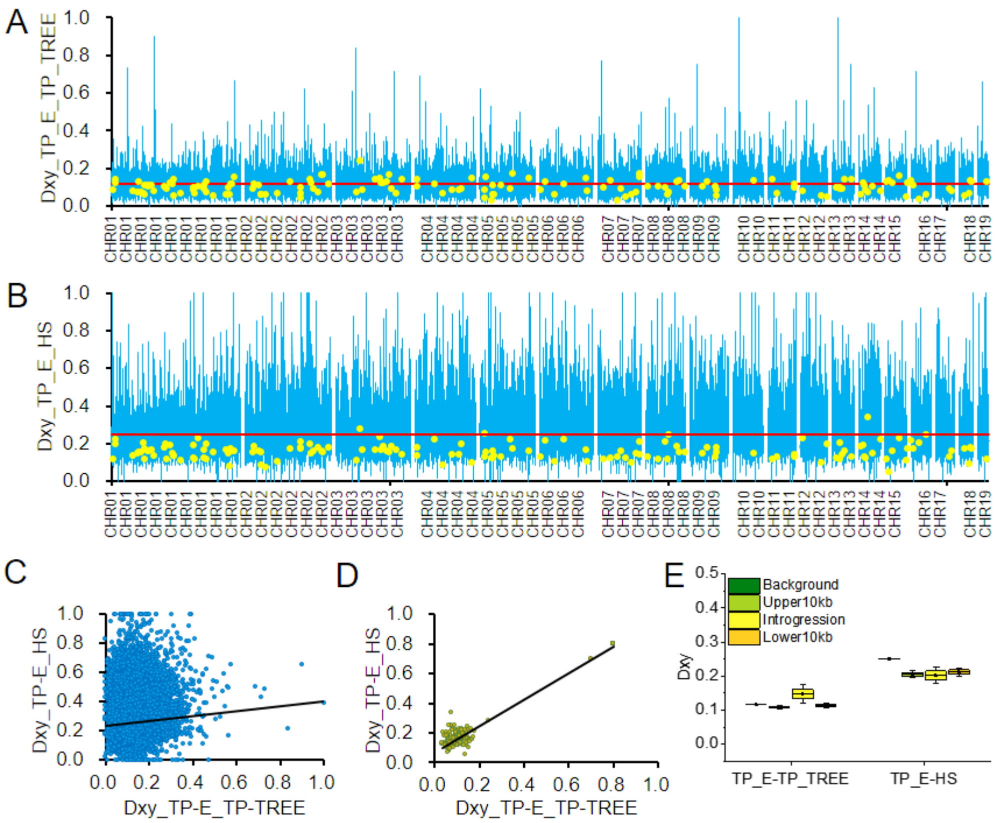

Supplemental Figure 21. Heterogeneity of absolute divergence (D_{xy}) among TP_E, TP_TREE, and HS.

(A) Pairwise absolute divergence (D_{xy}) was calculated in 10 kb sliding windows across 19 chromosomes between TP_TREE and TP_E.

(B) Pairwise absolute divergence (D_{xy}) was calculated in 10 kb sliding windows across 19 chromosomes between TP_E and HS.

(C) The pairwise absolute divergence (D_{xy}) of TP_TREE and TP_E, in comparison to HS and TP_E, within the genome.

(D) The pairwise absolute divergence (D_{xy}) of TP_TREE and TP_E, in comparison to HS and TP_E, within introgressed regions.

(E) The boxplot illustrates the average pairwise absolute divergence (D_{xy}) across all comparisons, including the genomic background, introgressed regions, and the upper and lower 10 kb regions.

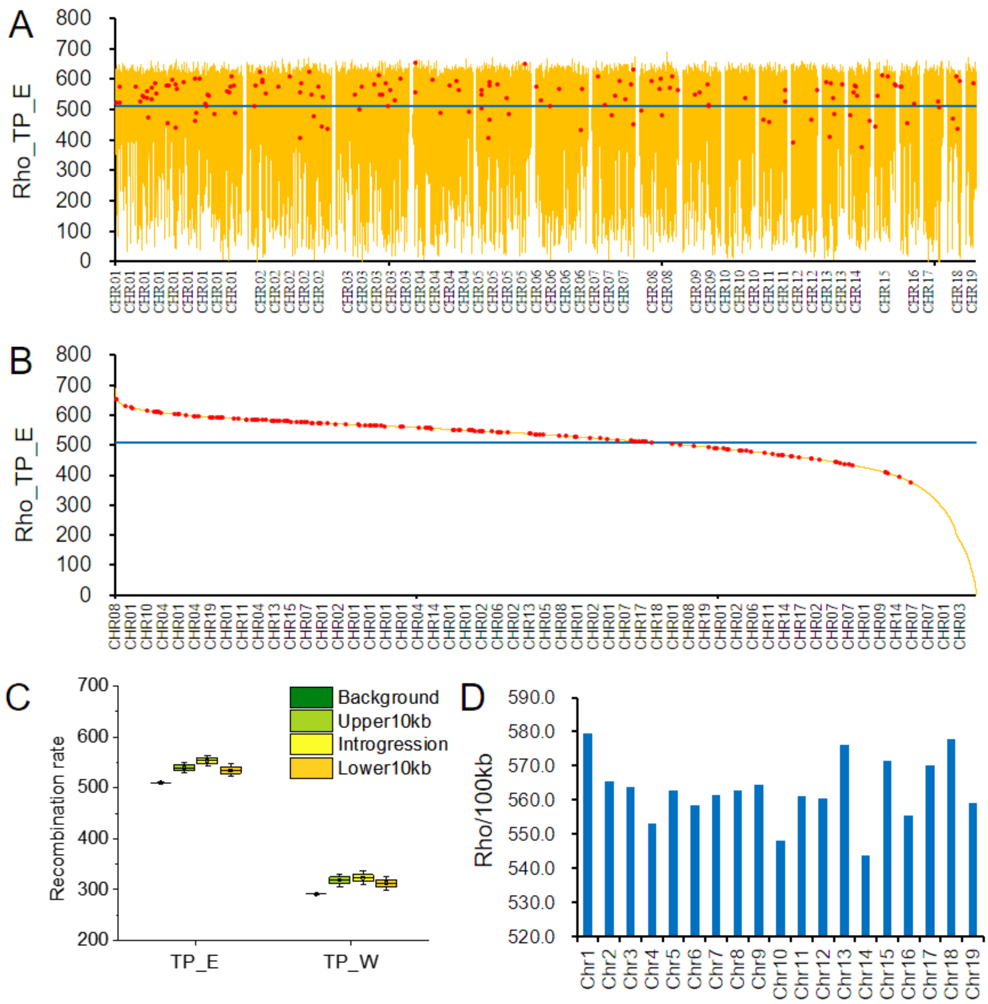

Supplemental Figure 22. The landscape of recombination rates across the genome and introgressed regions.

(A) The recombination rate is presented in 10 kb sliding windows across 19 chromosomes of TP_E. The genomic background is depicted in brown, while the introgressed regions are highlighted in red.

(B) The distribution pattern of recombination rate values in introgressed regions (red) is presented alongside the decline in the recombination rate within the surrounding genomic background (brown).

(C) The boxplot illustrates the average recombination rate across the genomic background, introgressed regions, and the upper and lower 10 kb regions.

(D) The average recombination rate across the 19 chromosomes of *D. catenatum*.

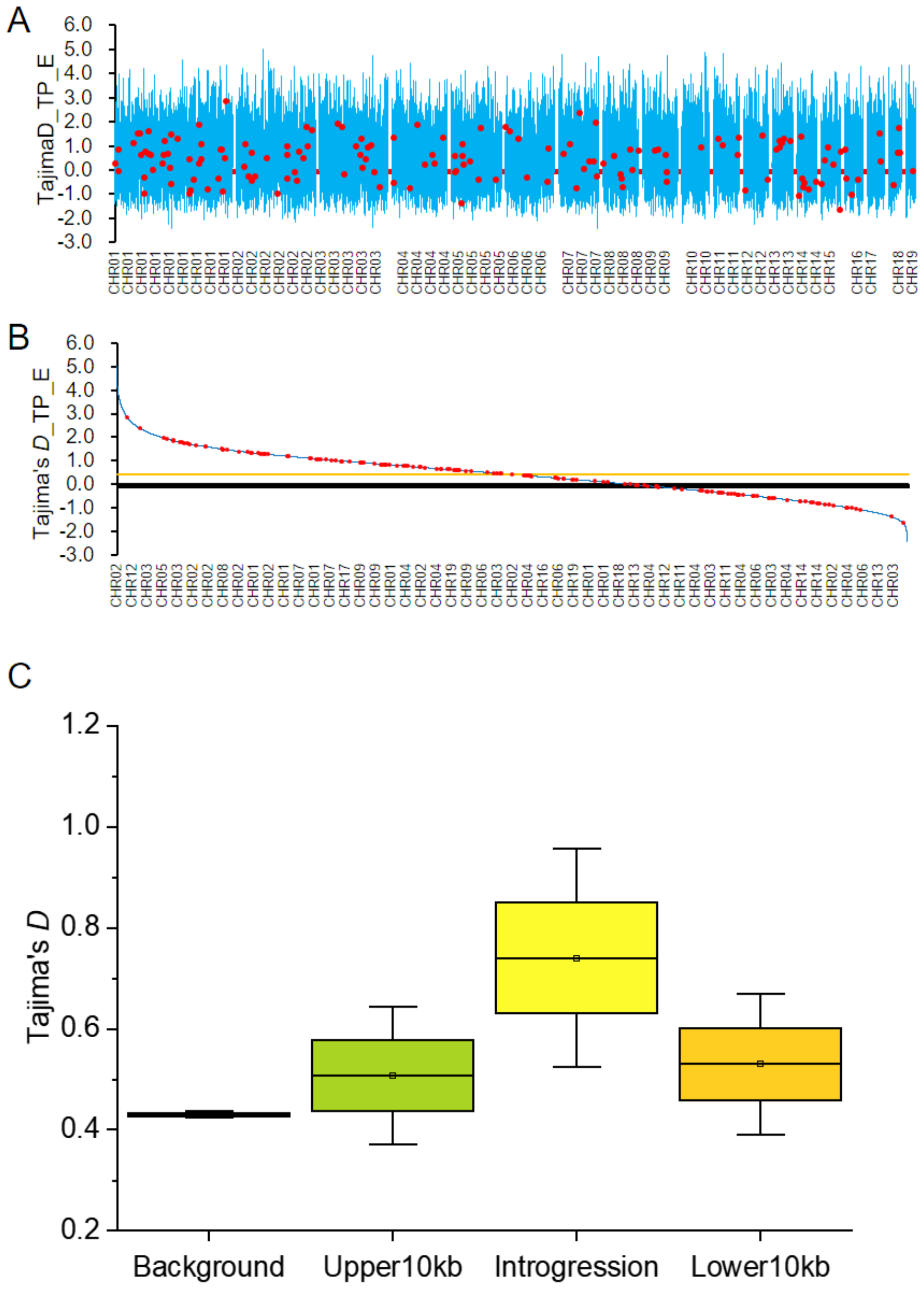

Supplemental Figure 23. The landscape of Tajima's *D* values across the genome and introgressed regions.

(A) Tajima's *D* values are presented in 10 kb sliding windows across 19 chromosomes of TP_E. The genomic background is indicated in blue, while the introgressed regions are highlighted in red.

(B) The distribution pattern of Tajima's *D* values in introgressed regions (red) is presented alongside the decline in the recombination rate within the genomic background (blue).

(C) The boxplot illustrates the average Tajima's *D* value across the genomic background, introgressed regions, and the upper and lower 10 kb regions.

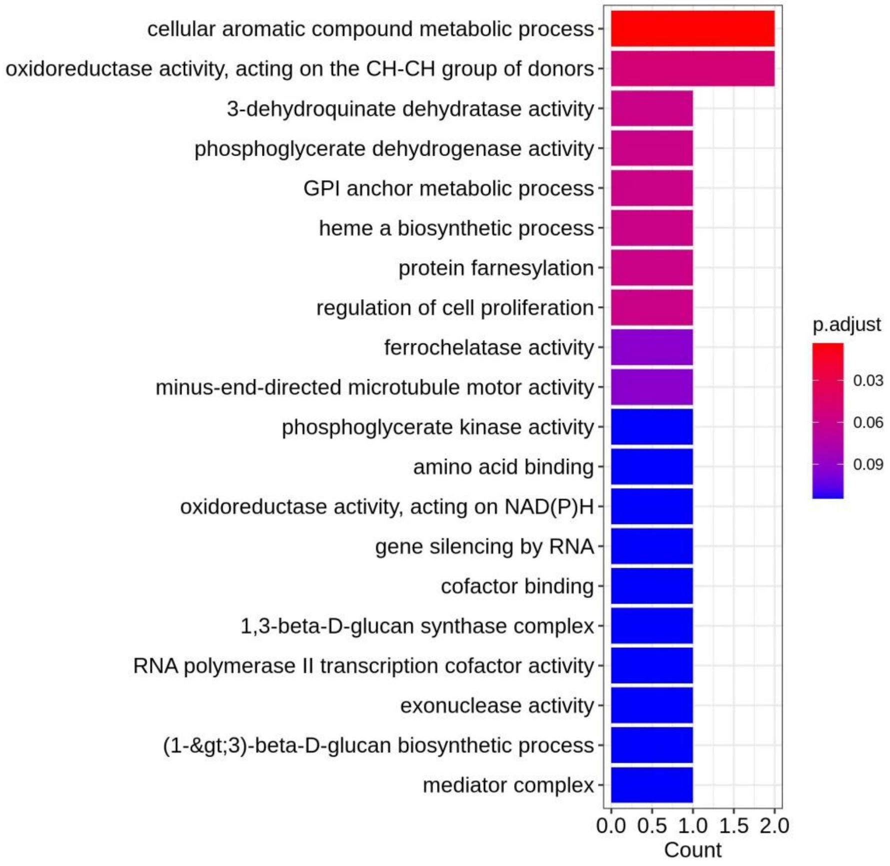

Supplemental Figure 24. GO term enrichment of introgressed genes.

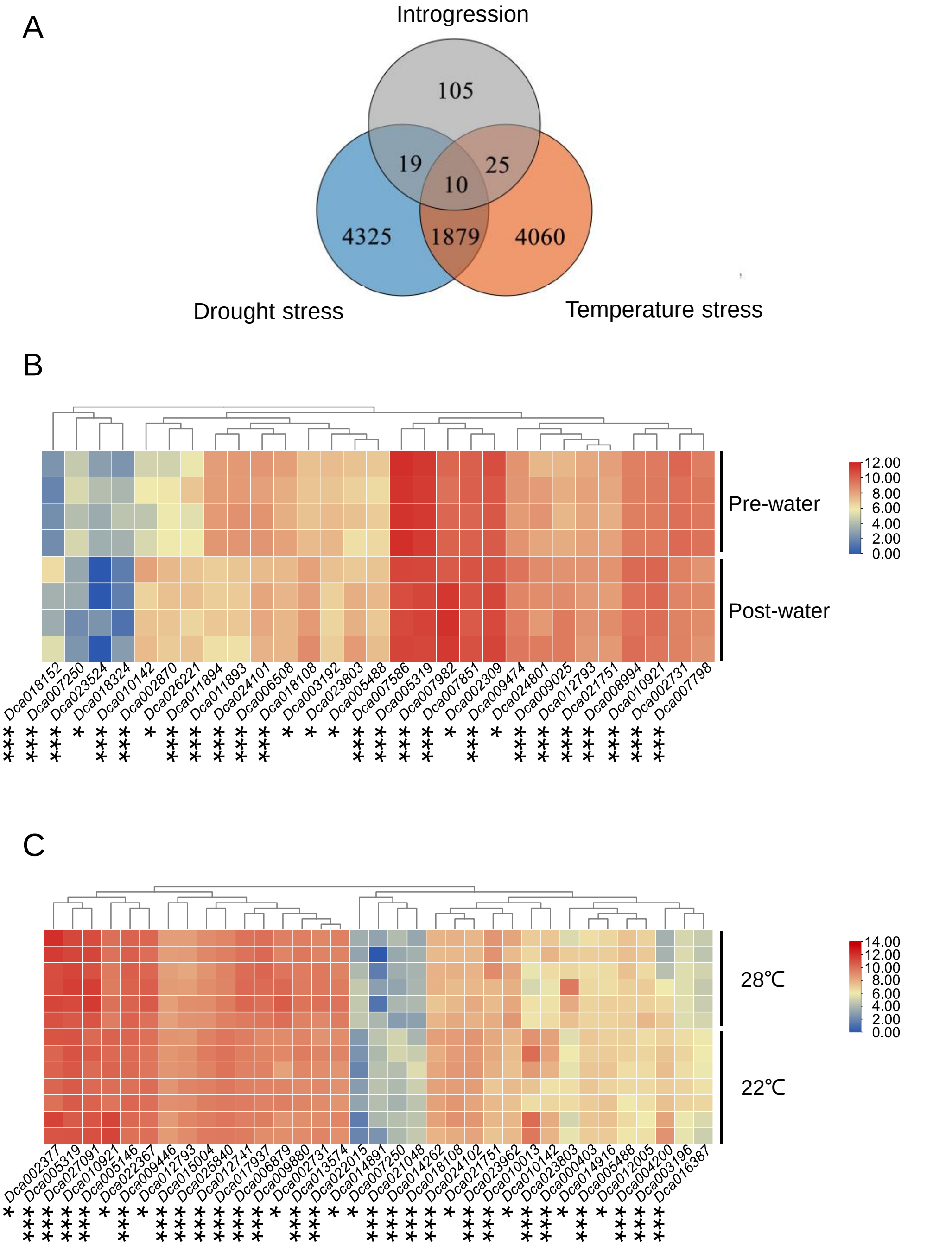

Supplemental Figure 25. Introgressed genes and their differential expression under abiotic stress conditions.

(A) The Venn diagram illustrates the number of introgressed genes in response to abiotic stress.

(B) The heatmap illustrates the expression of twenty-nine introgressed genes in response to drought stress.

(C) The heatmap illustrates the expression of thirty-five introgressed genes in response to temperature stress.

Asterisks denote significant differences (*, P < 0.05; **, P < 0.01; ***, P < 0.001) based on the Benjamini-Hochberg procedure.

A

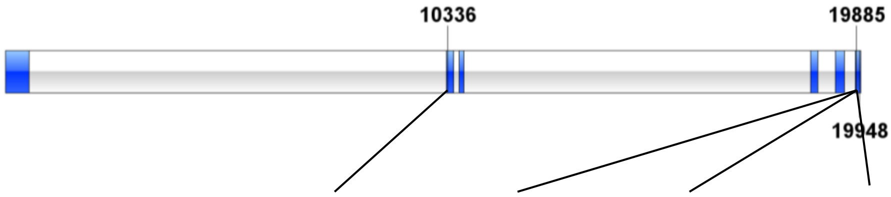

Species	No. of individuals	10336		19885		19947		19948	
		A	G	G	A	C	T	A	C
HS	31	77%	23%	61%	39%	81%	19%	81%	19%
TP_E	55	5%	95%	25%	75%	56%	44%	35%	65%
TP_M	24	25%	75%	33%	67%	50%	50%	21%	79%
TP_W	23	17%	83%	13%	87%	35%	65%	9%	91%

B

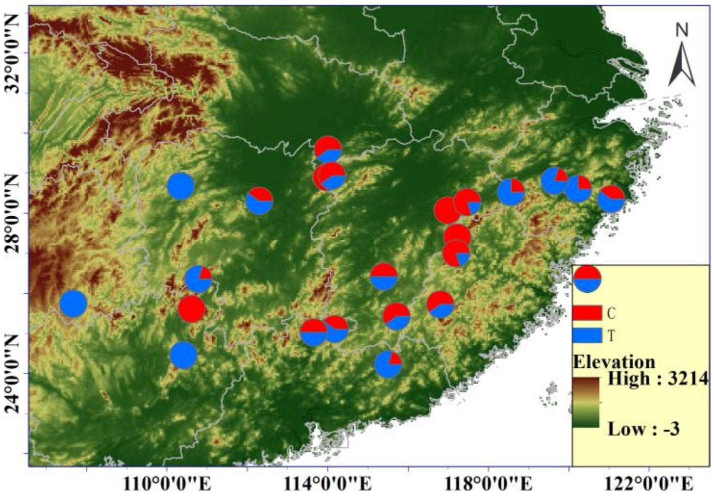

C

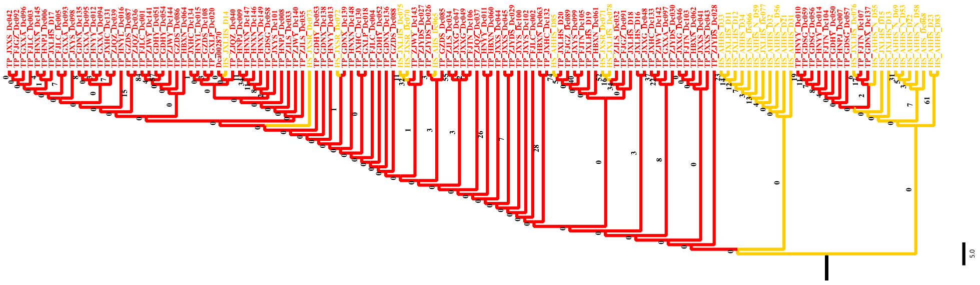

Supplemental Figure 26. Association of SNPs in *CDPK* with their geographic and phylogentic origins.

(A) SNPs in *Dendrobium huoshanense* and *D. catenatum* samples.

(B) The *D. huoshanense* and *D. catenatum* samples containing C at site 19947 are represented by a red pie chart, while the *D. catenatum* samples with T are denoted by a blue pie chart.

(C) The maximum likelihood phylogenetic tree of *CDPK* alleles detected in individuals of *D. huoshanense* (yellow) and *D. catenatum* (red).

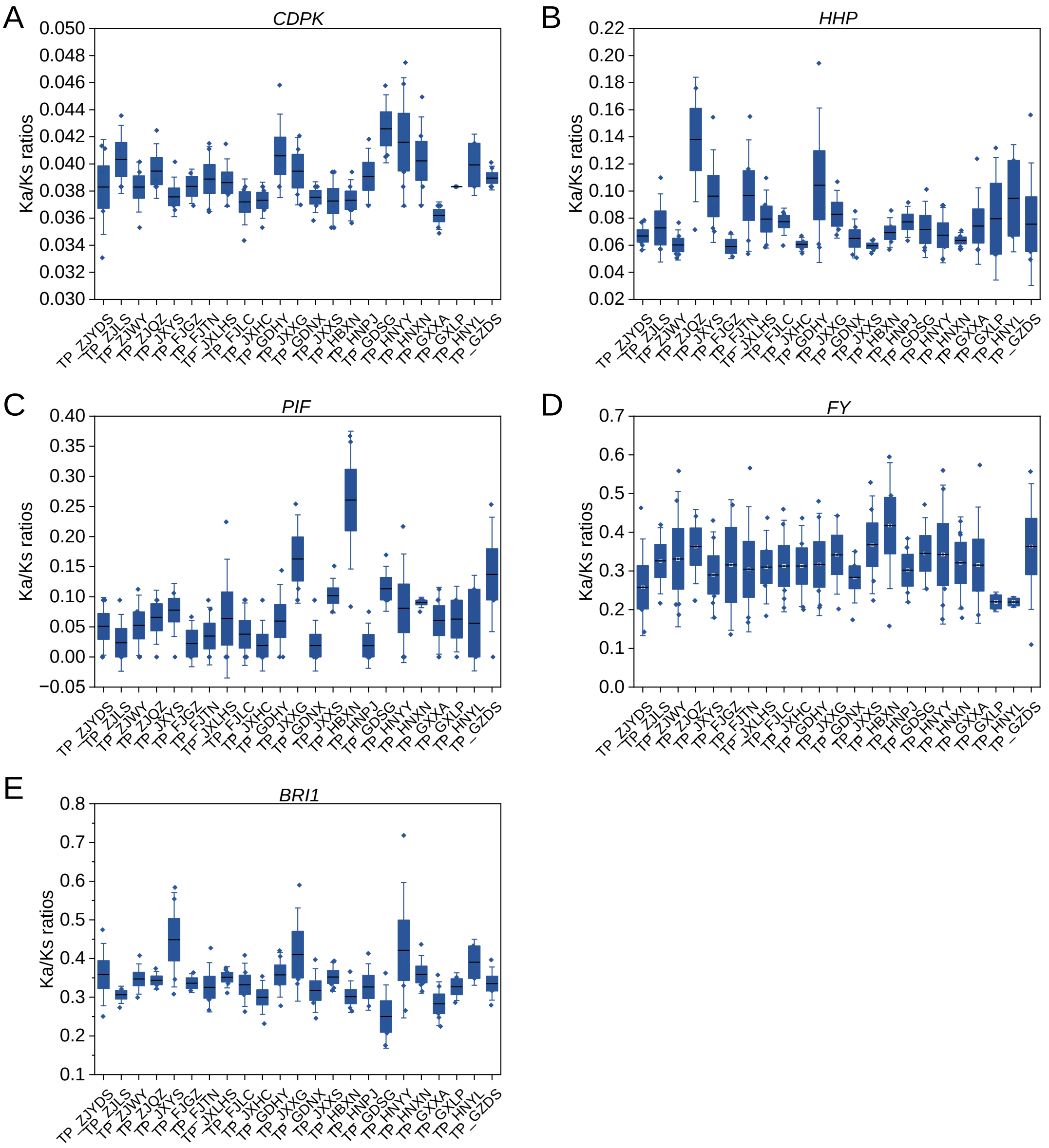

Supplemental Figure 27. Ka/Ks ratios of five introgressed genes between *D. huoshanense* and *D. catenatum*.

(A - E) The boxplot illustrates the Ka/Ks ratios of *CDPK*, *HHP*, *PIF*, *FY*, and *BRI1* between *D. huoshanense* and different *D. catenatum* populations, respectively.

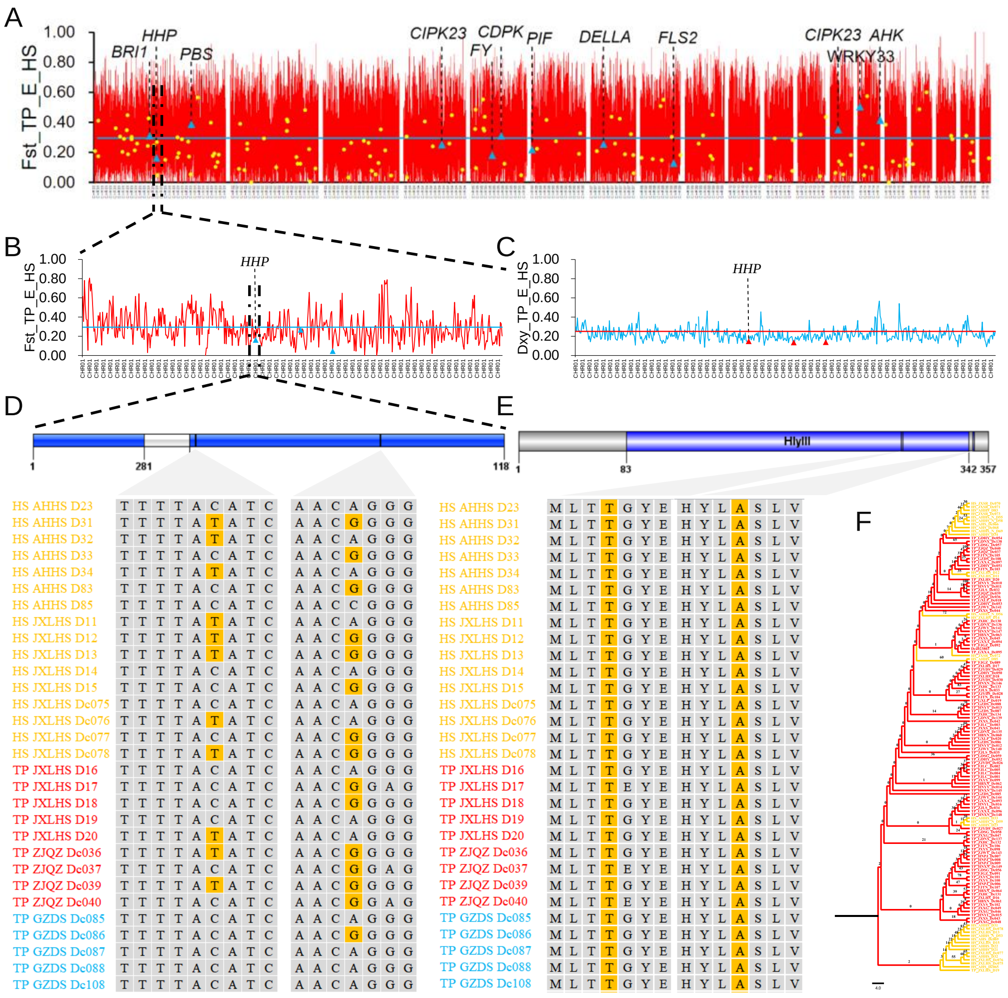

Supplemental Figure 28. Evidence of introgression at the *HHP* locus.

(A) Fixation index (F_{ST}) values were calculated in 10 kb windows across 19 chromosomes, comparing the eastern ecotype of *Dendrobium catenatum* (TP_E) with *D. huoshanense* (HS).

(B and C) Fixation index (F_{ST}) values (B) and absolute divergence (D_{xy}) values (C) across the *HHP* region.

(D and E) Representative substitutions of *HHP* in samples of *D. huoshanense* (yellow) and the eastern (red) and western (blue) ecotypes of *D. catenatum* are presented. Substitutions (brown) in nucleotides **(D)** and amino acids **(E)** are observed in the representative populations of *D. catenatum* and *D. huoshanense*.

(F) The maximum likelihood tree illustrates the *HHP* alleles detected in individuals of *D. huoshanense* (yellow) and *D. catenatum* (red).

The horizontal line represents the average value of the genome.

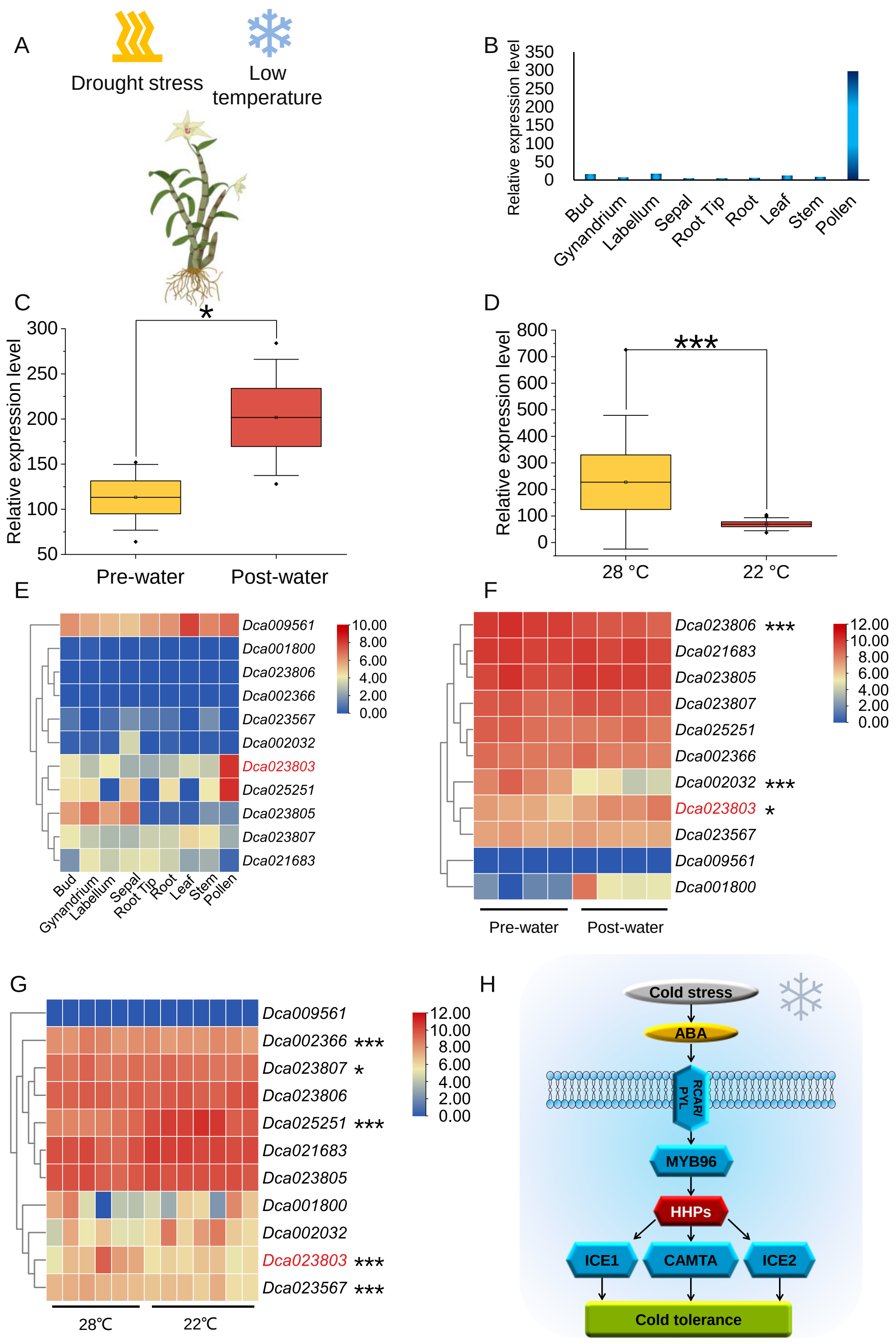

Supplemental Figure 29. Expression changes in *HHP* are correlated with the abiotic stress response in *Dendrobium catenatum*.

(A) Silhouette image of *Dendrobium catenatum* subjected to drought and low-temperature stress.

(B−G) The histogram and boxplot illustrate the transcriptome expression of *HHP* across nine tissues of *D. catenatum* **(B)**, as well as under drought stress **(C)** and temperature stress **(D)** conditions. Heatmaps illustrate the transcriptome expression of *HHP* and its paralogous

genes across the same nine tissues of *D. catenatum* **(E)**, as well as under drought stress **(F)** and temperature stress **(G)** conditions.

(H) A hypothetical cold stress signaling pathway in *D. catenatum*.

The introgressed genes are highlighted in red. Asterisks denote significant differences (*, $P < 0.05$; **, $P < 0.01$; ***, $P < 0.001$) based on the Benjamini-Hochberg procedure.

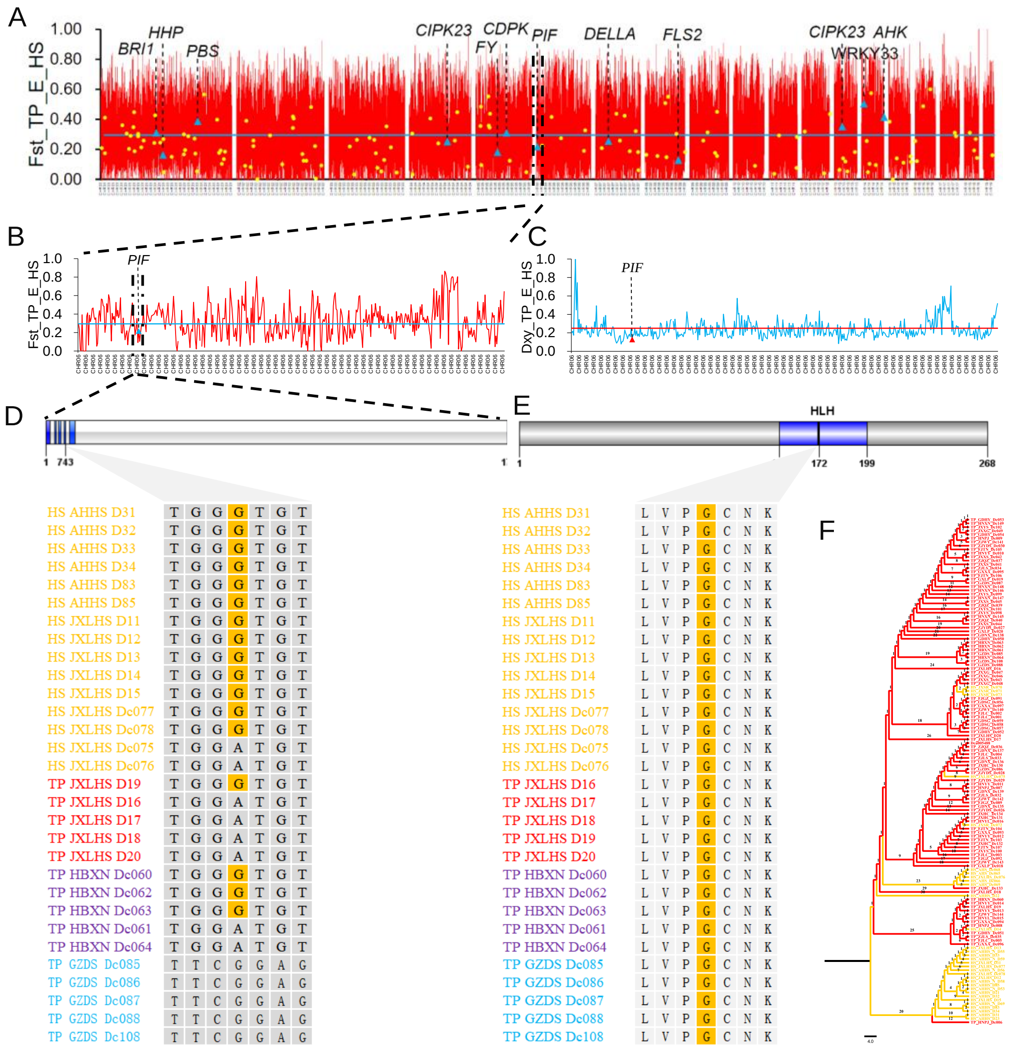

Supplemental Figure 30. Evidence of introgression at the *PIF* locus.

(A) Fixation index (F_{ST}) values were calculated in 10 kb windows across 19 chromosomes, comparing the eastern ecotype of *Dendrobium catenatum* (TP_E) with *D. huoshanense* (HS).

(B and C) Fixation index (F_{ST}) values **(B)** and absolute divergence (D_{xy}) values **(C)** across the *PIF* region.

(D and E) Representative substitutions of *PIF* in samples of *D. huoshanense* (yellow) and the eastern (red), central (purple), and western (blue) ecotypes of *D. catenatum* are presented. Substitutions (brown) in nucleotides **(D)** and amino acids **(E)** are observed in the representative populations of *D. catenatum* and *D. huoshanense*.

(F) The maximum likelihood tree of *PIF* alleles detected in individuals of *D. huoshanense* (yellow) and *D. catenatum* (red) is presented.

The horizontal line represents the average value of the genome.

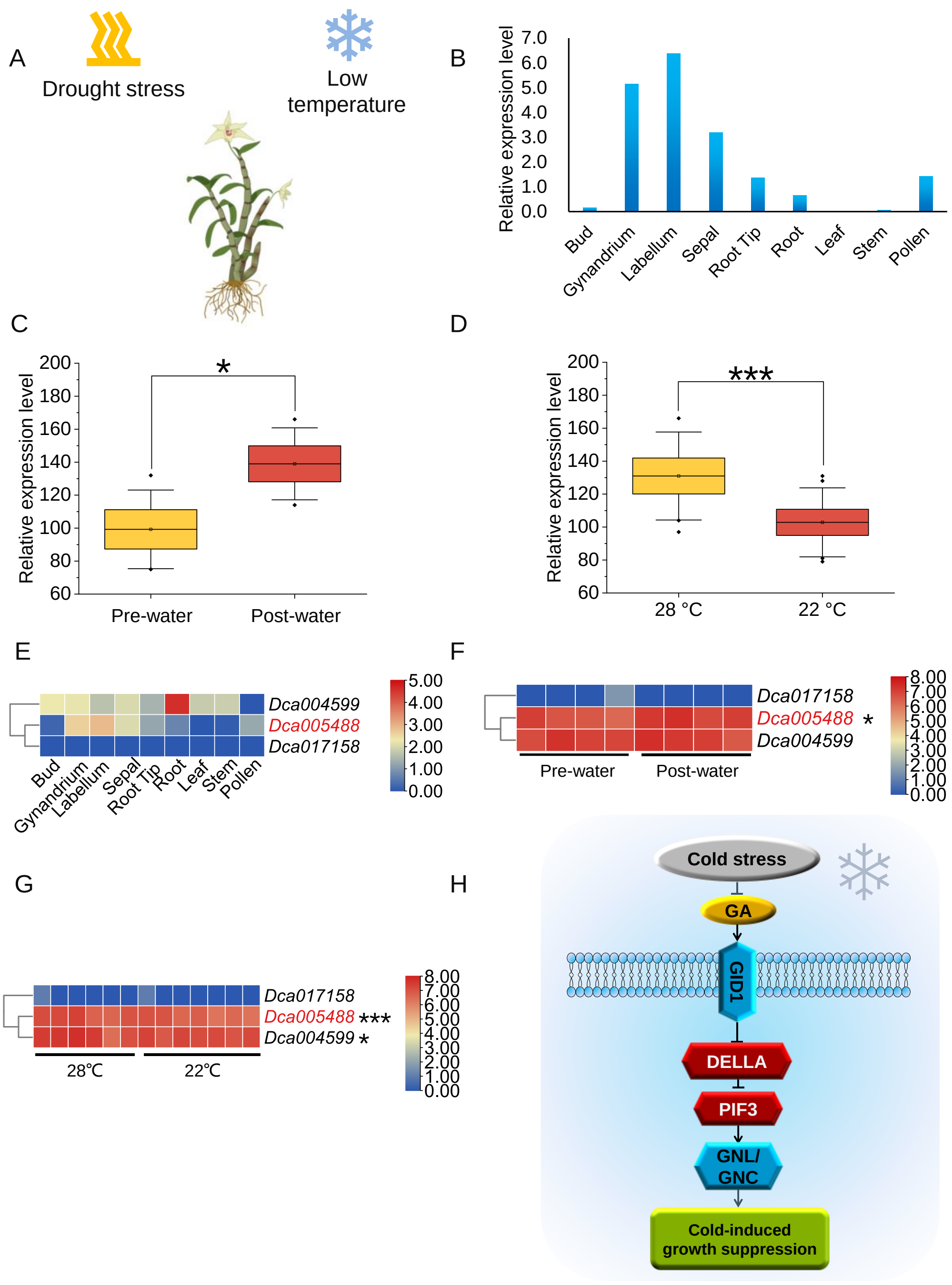

Supplemental Figure 31. Expression changes in *PIF* are correlated with the abiotic stress response in *Dendrobium catenatum*.

(A) Silhouette image of *D. catenatum* subjected to drought and low-temperature stress.

(B−G) The histogram and boxplot illustrate the transcriptome expression of *PIF* across nine tissues of *D. catenatum* **(B)**, as well as under drought stress **(C)** and temperature stress **(D)** conditions. Heatmaps illustrate the transcriptome expression of *PIF* and its paralogous genes across the same nine tissues of *D. catenatum* **(E)**, as well as under drought stress **(F)** and temperature stress **(G)** conditions.

(H) A hypothetical cold stress signaling pathway in *D. catenatum*.

The introgressed genes are highlighted in red. Asterisks denote significant differences (*, $P < 0.05$; **, $P < 0.01$; ***, $P < 0.001$) based on the Benjamini-Hochberg procedure.

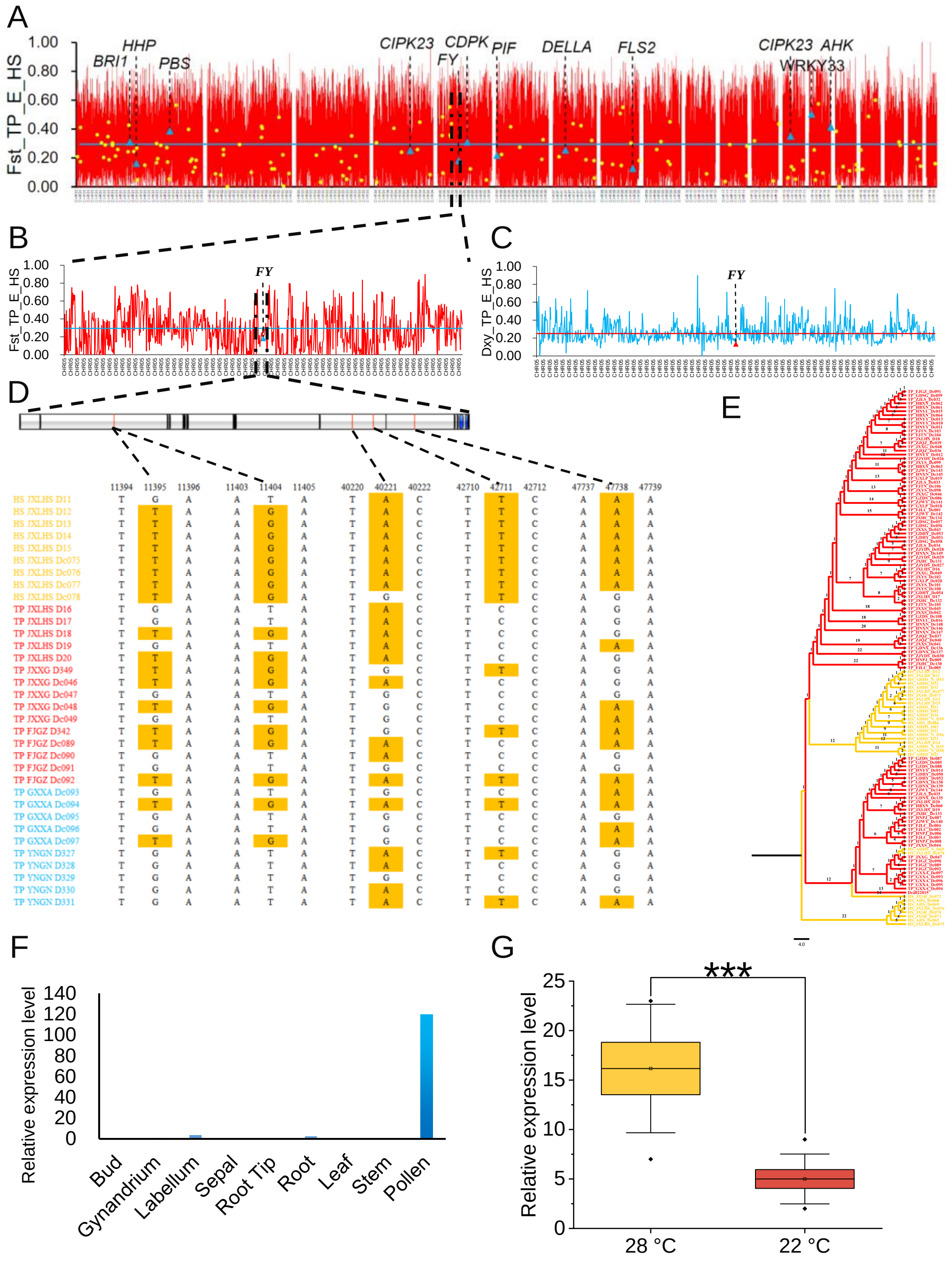

Supplemental Figure 32. Evidence of introgression at the *FY* locus and expression changes correlated with the abiotic stress response in *Dendrobium catenatum*.

(A) Fixation index (F_{ST}) values were calculated in 10 kb windows across 19 chromosomes, comparing the eastern ecotype of *D. catenatum* (TP_E) with *D. huoshanense* (HS).

(B-C) Fixation index (F_{ST}) values **(B)** and absolute divergence (D_{xy}) values **(C)** across the *FY* region.

(D) Representative substitutions of *FY* in samples of *D. huoshanense* (yellow) and the eastern (red) and western (blue) ecotypes of *D. catenatum* are presented. Substitutions (brown) in nucleotides are observed in the representative populations of *D. catenatum* and *D. huoshanense*.

(E) The maximum likelihood tree of *FY* alleles detected in individuals of *D. huoshanense* (yellow) and *D. catenatum* (red).
(F) The histogram illustrates the expression of the *FY* transcriptome across nine tissues of *D. catenatum*.
(G) The boxplot illustrates the expression levels of the *FY* transcriptome under drought stress conditions.
The horizontal line represents the average value of the genome. Asterisks denote significant differences (*, $P < 0.05$; **, $P < 0.01$; ***, $P < 0.001$) based on the Benjamini-Hochberg procedure.

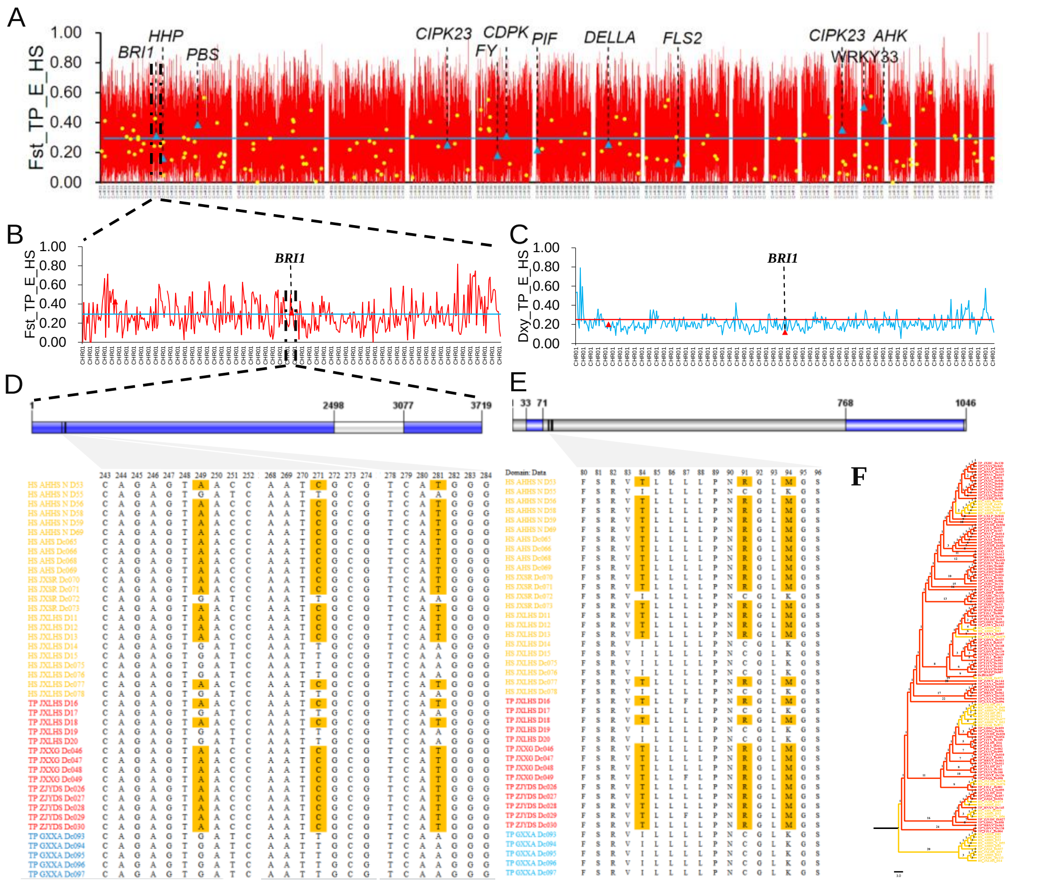

Supplemental Figure 33. Evidence of introgression at the *BRI1* locus.

(A) Fixation index (F_{ST}) values were calculated in 10 kb windows across 19 chromosomes, comparing the eastern ecotype of *Dendrobium catenatum* (TP_E) with *D. huoshanense* (HS).

(B-C) Fixation index (F_{ST}) values **(B)** and absolute divergence (D_{xy}) values **(C)** across the *BRI1* region.

(D−E) Representative substitutions of *BRI1* in samples of *D. huoshanense* (yellow) and the eastern (red) and western (blue) ecotypes of *D. catenatum* are presented. Substitutions (brown) in nucleotides **(D)** and amino acids **(E)** are observed in the representative populations of *D. catenatum* and *D. huoshanense*.

(F) The maximum likelihood tree of *BRI1* alleles detected in individuals of *D. huoshanense* (yellow) and *D. catenatum* (red).

The horizontal line represents the average value of the genome.

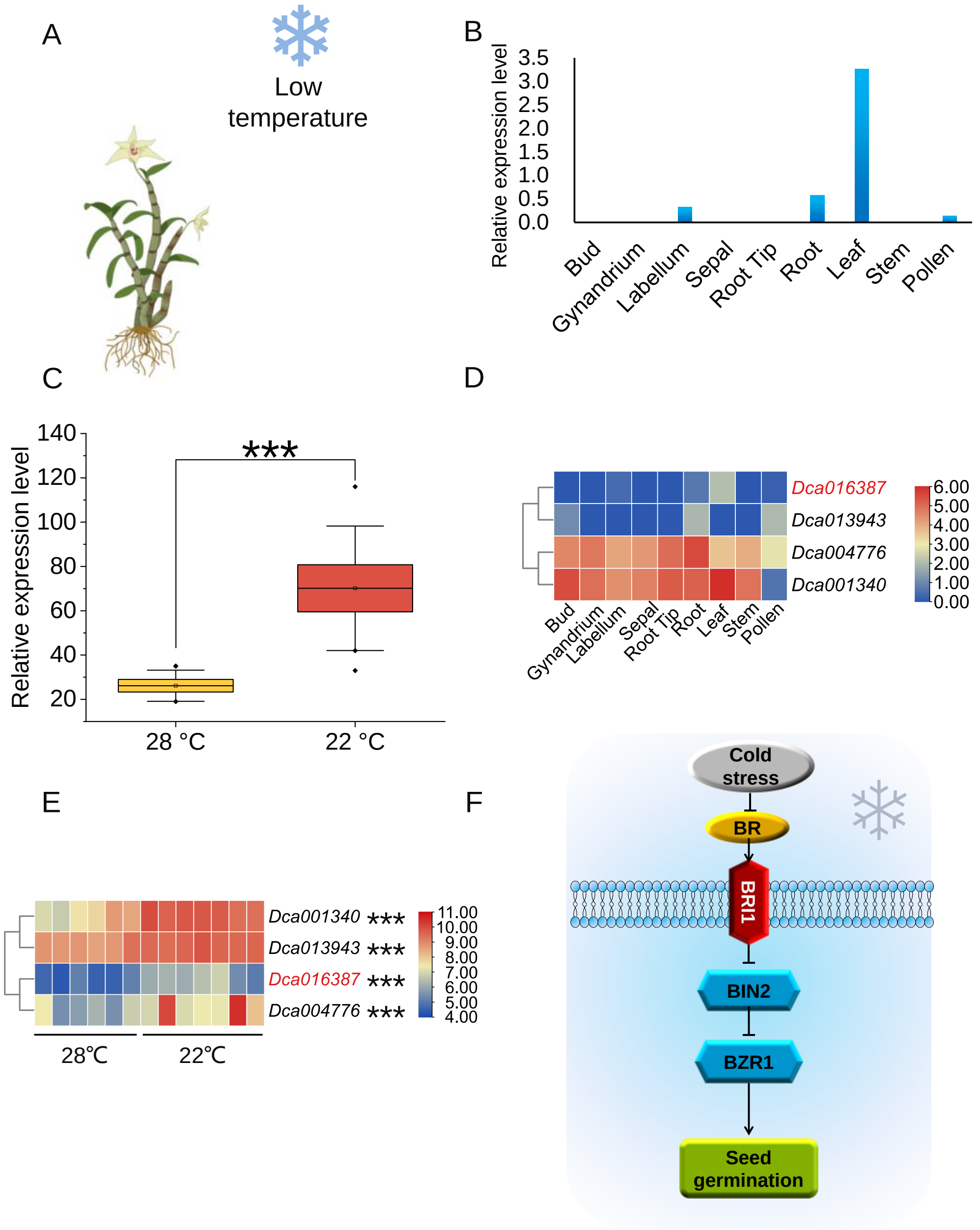

Supplemental Figure 34. Expression changes in *BRI1* are correlated with the abiotic stress response in *Dendrobium catenatum*.

(A) Silhouette image of *D. catenatum* subjected to low-temperature stress.

(B and C) The histogram and boxplot illustrate the transcriptome expression of *BRI1* across nine tissues of *D. catenatum* **(B)**, and under temperature stress **(C)** conditions.

(D and E) Heatmaps illustrate the expression of the transcriptome for *BRI1* and its paralogous genes across nine tissues of *D. catenatum* **(D)**, and under temperature stress **(E)** conditions.

(F) A hypothetical cold stress signaling pathway in *D. catenatum*.

The introgressed genes are highlighted in red. Asterisks denote significant differences (*, $P < 0.05$; **, $P < 0.01$; ***, $P < 0.001$) based on the Benjamini-Hochberg procedure.

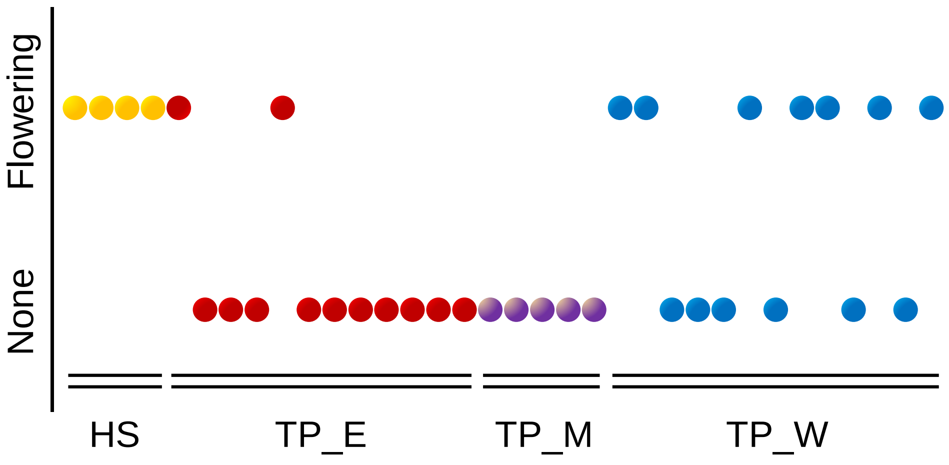

Supplemental Figure 35. The flowering phenology of *Dendrobium huoshanense* and *D. catenatum* samples under common garden conditions from April 24, 2018, to May 24, 2018.

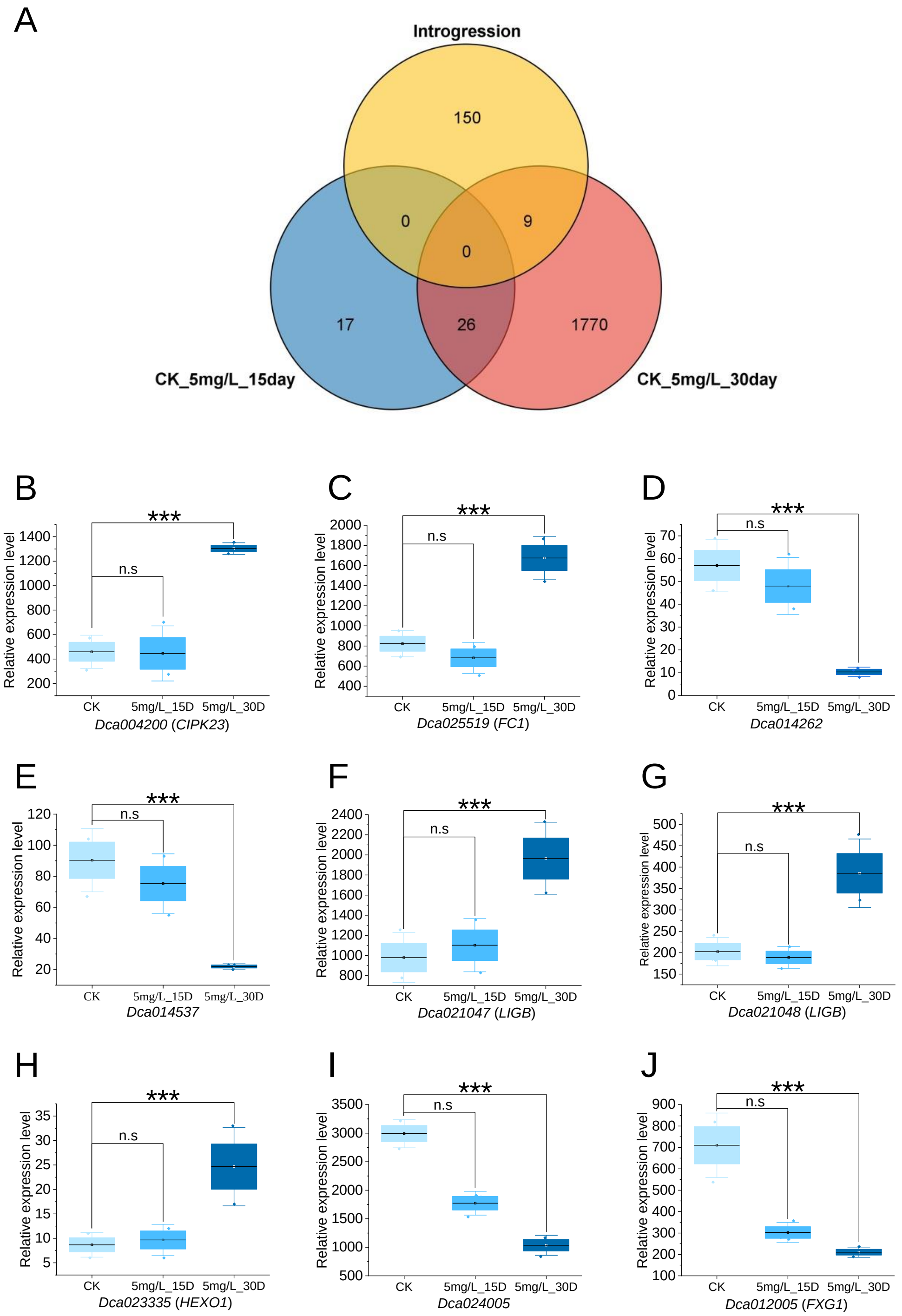

Supplemental Figure 36. Introgressed genes and their differential expression under cadmium (Cd) stress.

(A) The Venn diagram illustrates the number of introgressed genes and the differentially expressed gene responses to cadmium stress.

(B-J) The expression patterns of nine introgressed genes in response to cadmium stress. Asterisks denote significant differences (*, $P < 0.05$; **, $P < 0.01$; ***, $P < 0.001$) based on the Benjamini-Hochberg procedure and log2 fold change (log_2FC). “n.s” indicates no significant difference ($P > 0.05$).

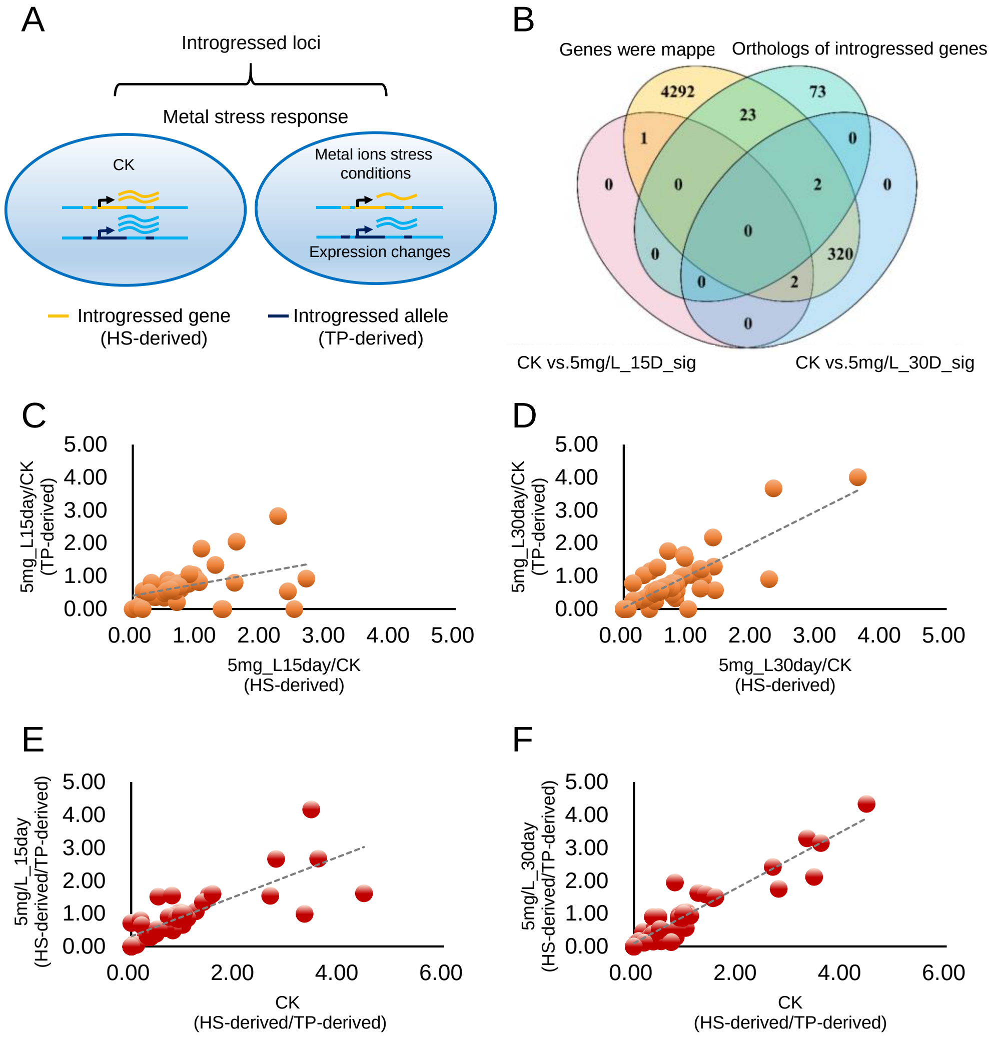

Supplemental Figure 37. The differential expression of introgressed genes and their alleles in response to cadmium (Cd) stress.

(A) Schematic illustration of introgressed genes and their alleles in response to different metal ion stresses.

(B) The Venn diagram illustrates the number of genes mapped to the *Dendrobium huoshanense* genome reference (Genes were mapped), the orthologs of introgressed genes, and the differentially expressed genes mapped to the *D. huoshanense* genome under 5mg/L cadmium for 15 days (5mg/L_15D_sig) and 30days (5mg/L_30D_sig).

(C) The pairwise ratio of the relative expression levels of HS-derived genes to TP-derived genes at 5mg/L_15D compared to the control (CK).

(D) The pairwise ratio of the relative expression levels of HS-derived genes to TP-derived genes at 5mg/L_30D compared to the control (CK).

(E) The pairwise ratio of the relative expression levels of HS-derived genes to TP-derived genes under 5mg/L_15D and control conditions, respectively.

(F) The pairwise ratio of the relative expression levels of HS-derived genes to TP-derived genes under 5mg/L_30D and control conditions, respectively.

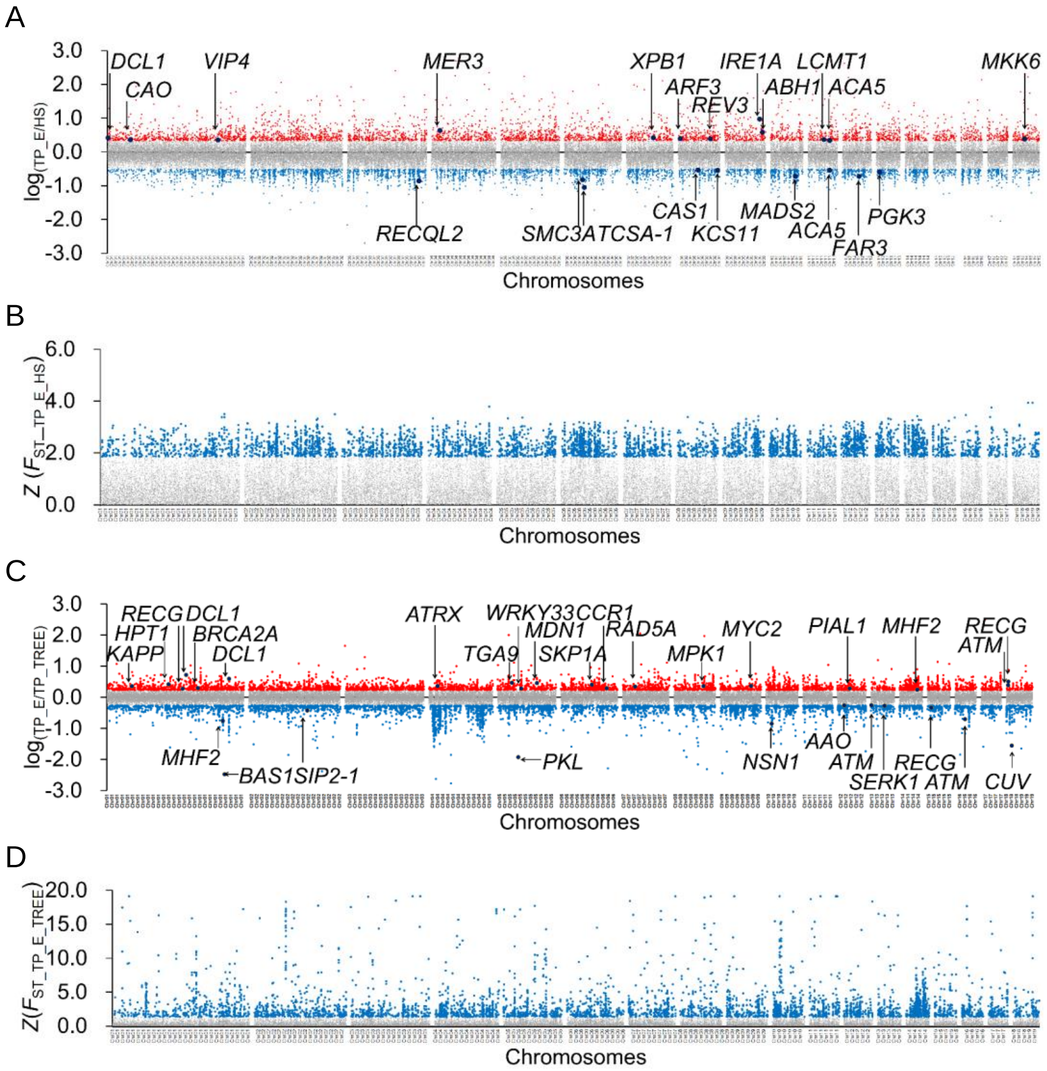

Supplemental Figure 38. Detection of positive selection in the ecotypes of *Dendrobium huoshanense* and *D. catenatum*.

(A–B). Positively selected genomic signatures between the eastern ecotype of *D. catenatum* (TP_E) and *D. huoshanense* (HS) populations were identified using θ_π ratios and Z(F_{ST}) metrics. Red and blue dots indicate genomic regions under significant selection ($p < 0.05$). Analyses were conducted using a sliding window size of 10 kb. Arrows highlight candidate genes associated with abiotic stress response, reproductive organ development, and regulation of the DNA damage response.

(C–D). Positively selected genomic signatures between the eastern (TP_E) and western (TP_TREE) ecotypes of *D. catenatum* populations were identified using θ_π ratios and Z(F_{ST}) metrics. Red and blue dots indicate genomic regions under significant selection ($p < 0.05$). Analyses were conducted using a sliding window size of 10 kb. Arrows highlight candidate genes associated with abiotic stress response, reproductive organ development, and regulation of the DNA damage response.

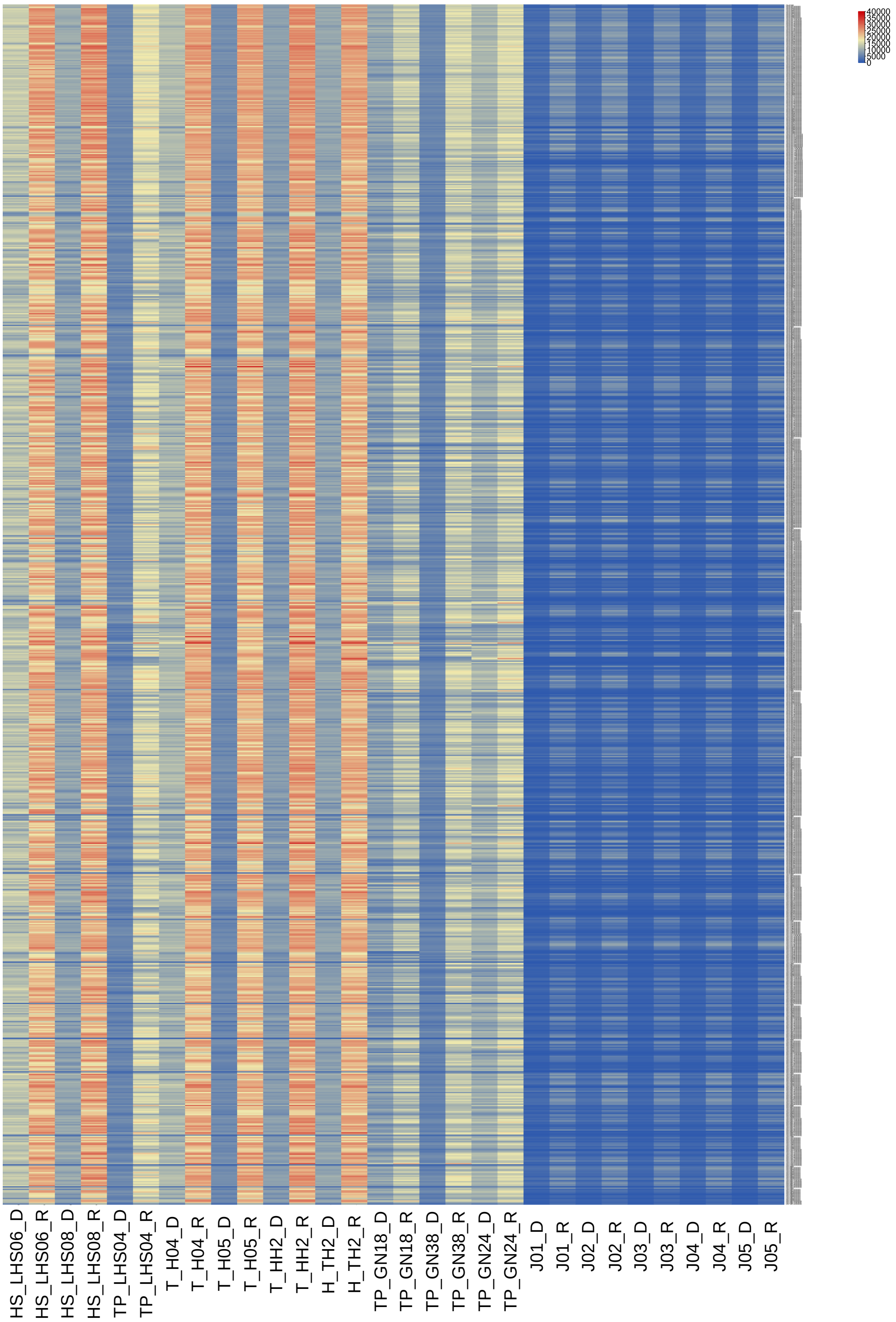

Supplemental Figure 39. Genomic variation density (per 1-Mb segment) across 19 chromosomes, detected using restriction site-associated DNA sequencing (RAD-Seq; D) and whole-genome resequencing (WGR; R).

Populations include *Dendrobium huoshanense* (HS), *D. catenatum* (TP), F_1 hybrids (T_H: ♀TP × ♂HS; H_T: ♀HS × ♂TP), and the outgroup *Flickingeria albopurpurea* (J).

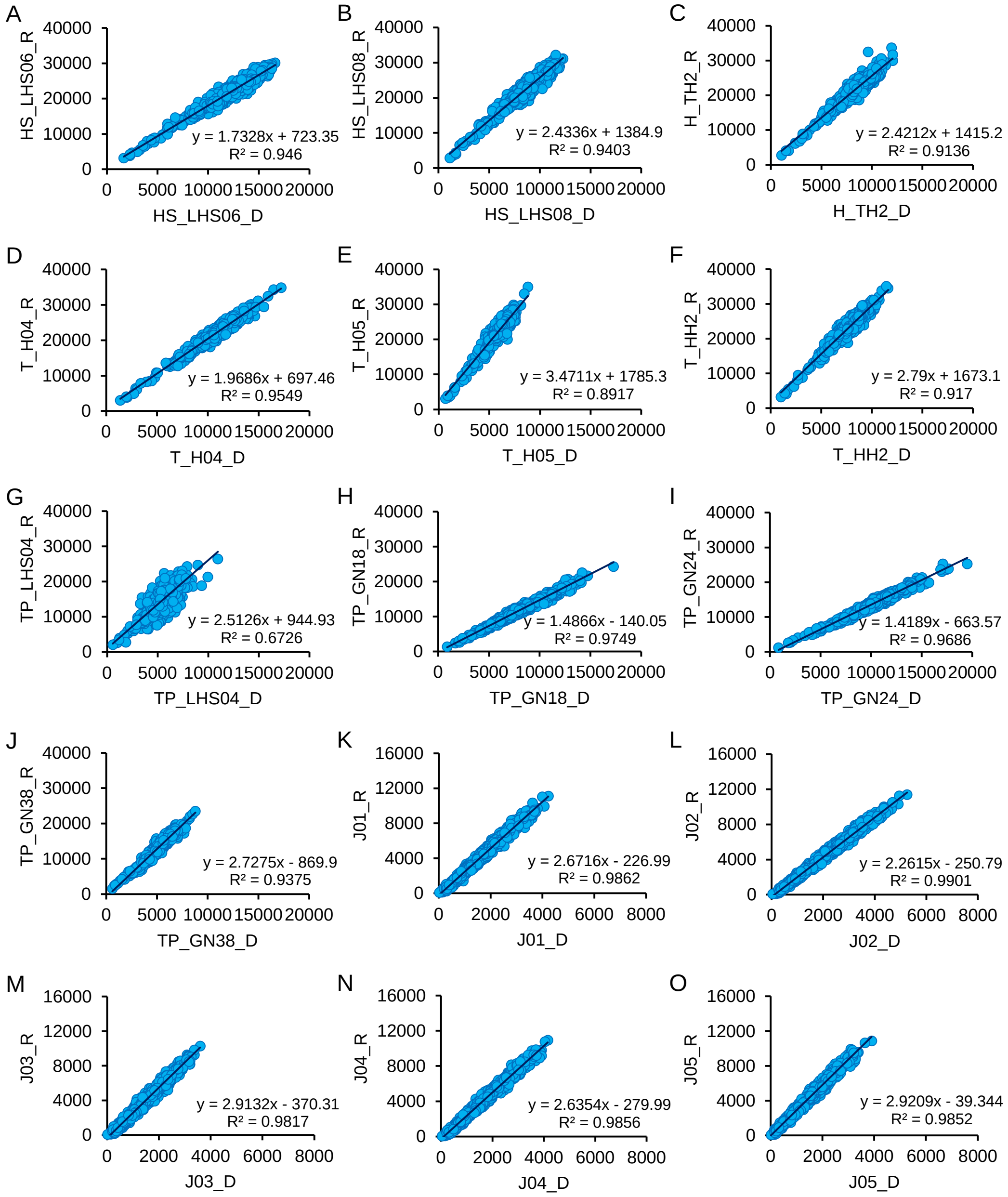

Supplemental Figure 40. Correlation of genomic variation density between restriction site-associated DNA sequencing (RAD-Seq; D) and whole-genome resequencing (WGR; R) across 19 chromosomes in *Dendrobium* lineages.

Variation density was quantified using a 1-Mb sliding genomic window. Panels A–B represent *D. huoshanense* (HS); panels C–F show F_1 hybrids of *D. huoshanense* × *D. catenatum* (T_H: ♀TP × ♂HS; H_T: ♀HS × ♂TP); panels G–J depict *D. catenatum* (TP); and panels K–O correspond to the outgroup *Flickingeria albopurpurea* (J).

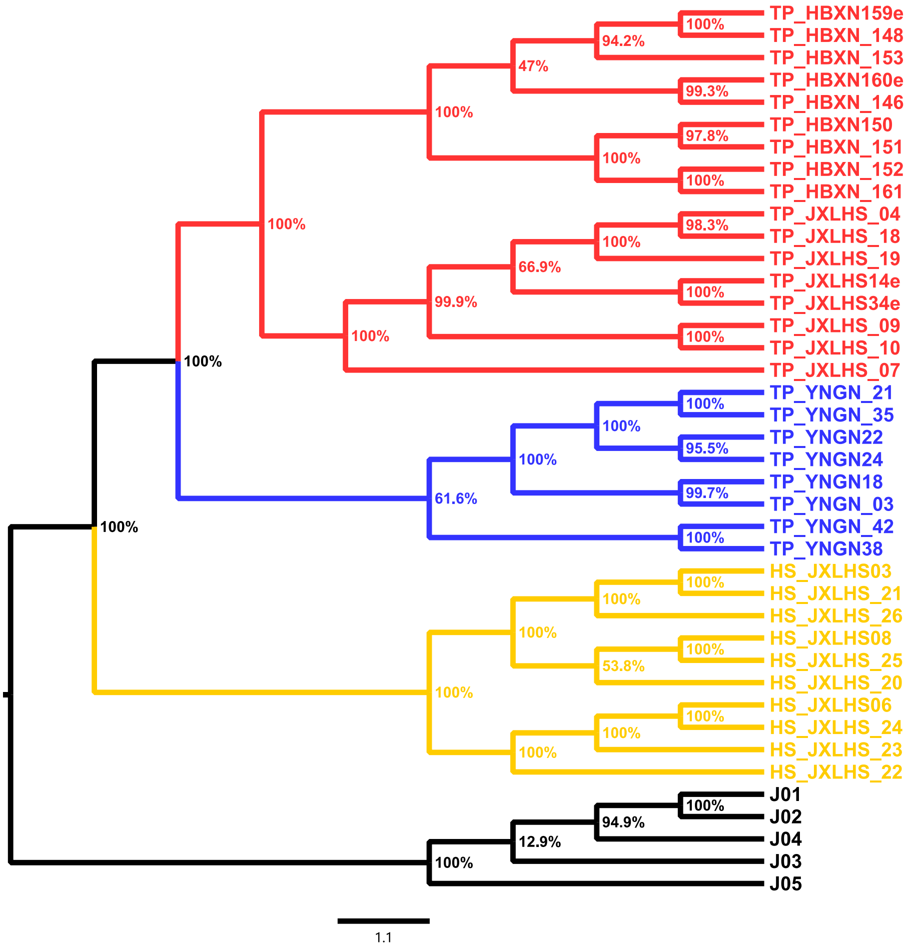

Supplemental Figure 41. Maximum likelihood phylogenetic analysis of *Dendrobium huoshanense* (HS) and *D. catenatum* (TP) populations.

The cladogram illustrates the evolutionary relationships among three populations of *D. catenatum* (TP_JXLHS, TP_HBXN, TP_YNGN) and one population of *D. huoshanense* (HS_JXLHS), with *Flickingeria albopurpurea* (J) serving as the outgroup. Colors correspond to specific habitats: red represents a lithophytic habitat, and blue represents an epiphytic habitat.

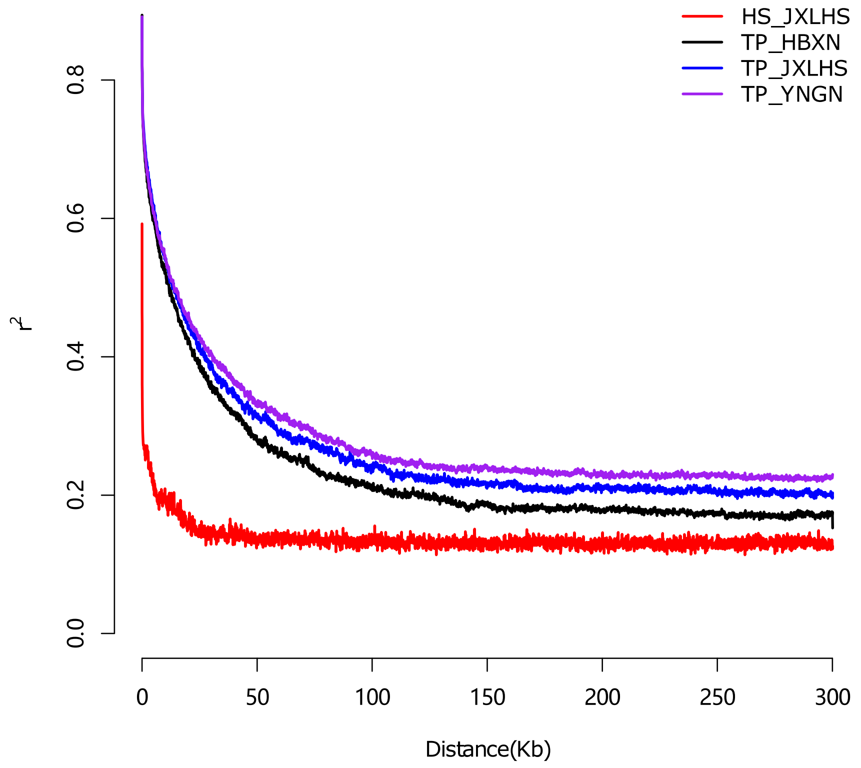

Supplemental Figure 42. Patterns of linkage disequilibrium (LD) decay (r^2) across genetic distances in four *Dendrobium* populations.

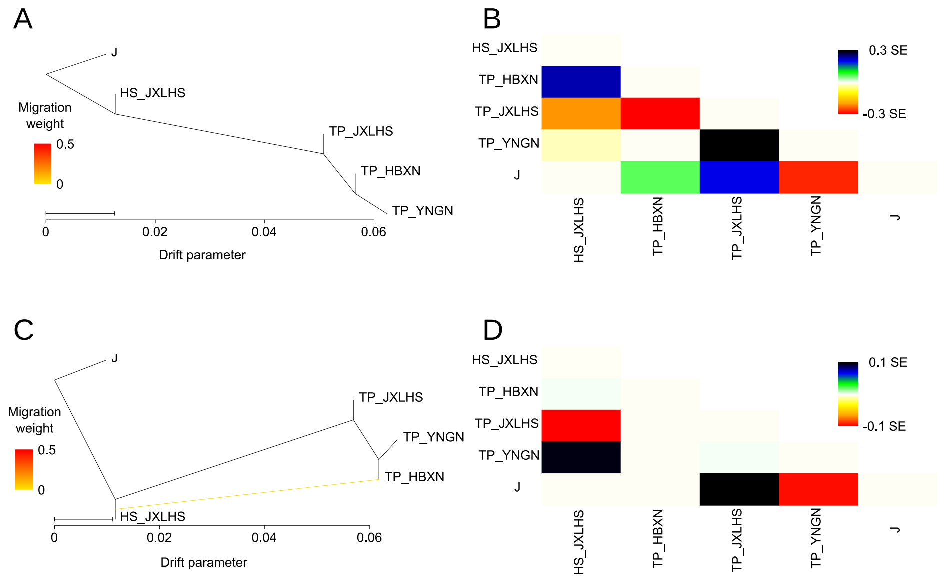

Supplemental Figure 43. Migration events between *Dendrobium huoshanense* and three *D. catenatum* populations inferred using TreeMix.

(A) The maximum likelihood tree was inferred using TreeMix without incorporating any migration edges.

(B) The residuals of the model fit, which do not account for migration events, exhibit high positive standard error (SE) values.

(C) The maximum likelihood tree inferred using TreeMix, allowing for one migration edge, is illustrated with arrows colored according to the migration weight.

(D) Residuals of the model fitted with a single migration event.

Supplemental Table 1. Overview of sample information and sequencing statistics.

Supplemental Table 2. Molecular diversity indices of three ecotypes of *Dendrobium catenatum* and *D. huoshanense*.

	N	π	H_O	H_E	Ts/Tv
HS	37	0.0968	0.2071	0.2001	2.1205
TP_E	60	0.0830	0.1635	0.1668	2.0472
TP_M	25	0.0741	0.1855	0.2121	1.9295
TP_W	34	0.0822	0.1831	0.1876	2.1503

Number of individuals (*N*), nucleotide diversity (π), observed heterozygosity (H_O), expected heterozygosity (H_E), and Transition-Transversion ratio (Ts/Tv) are listed in the genomic coding regions. The western (TP_W), central (TP_M), and eastern ecotypes (TP_E) of *D. catenatum* and *D. huoshanense* (HS) are listed in the table.

Supplemental Table 3. Pairwise F_{ST}s values between *Dendrobium huoshanense* and three ecotypes of *D. catenatum*.

	HS	TP_E	TP_M	TP_W
HS	0.0000			
TP_E	0.4386	0.0000		
TP_M	0.4367	0.0127	0.0000	
TP_W	0.4345	0.0229	0.0202	0.0000

The western (TP_W), central (TP_M), and eastern ecotypes (TP_E) of *D. catenatum* and *D. huoshanense* (HS) are listed in the table.

Supplemental Table 4. Differences in phenotypic traits of stems and flowers between *Dendrobium huoshanense* and *D. catenatum*.

	D. huoshanense	*D. catenatum*
No. of stems	355	414
Length (cm)	5.80 ± 2.65^{A}	14.97 ± 8.26^{B}
Diameter (cm)	0.69 ± 0.13^{A}	0.53 ± 0.11^{B}
Length-diameter ratio	8.64 ± 4.24^{A}	30.16 ± 19.40^{B}
Internode length (cm)	1.26 ± 0.36^{A}	1.65 ± 0.57^{B}
No. of internodes	5.75 ± 1.28^{A}	11.17 ± 3.21^{B}
No. of flowers	107	235
Dorsal sepal length (cm)	1.29 ± 0.19^{A}	1.77 ± 0.23^{B}
Dorsal sepal width (cm)	0.66 ± 0.11^{A}	0.63 ± 0.07^{A}
Length-width ratio of dorsal sepal	2.02 ± 0.34^{A}	2.83 ± 0.40^{B}
Petal length (cm)	1.28 ± 0.21^{A}	1.67 ± 0.20^{B}
Petal width (cm)	0.77 ± 0.14^{A}	0.56 ± 0.07^{B}
Length-width ratio of petal	1.71 ± 0.30^{A}	3.01 ± 0.46^{B}
Pedicel length (cm)	2.11 ± 0.44^{A}	1.97 ± 0.37^{A}

An identical superscript capital letter in the same row indicates no significant difference ($P > 0.05$), and different superscript capital letters in the same row indicate an extremely significant difference ($P < 0.01$).

Supplemental Table 5. Complex patterns of historical introgression between *Dendrobium catenatum* (TP_E, TP_M, and TP_W) and *D. huoshanense* (HS).

H1	H2	H3	H4	JK-*D*	V(JK-*D*)	*Z*
HS	TP_M	TP_E	J	0.8244	0.0000	766.52
HS	TP_W	TP_E	J	0.8096	0.0000	772.81
HS	TP_E	TP_M	J	0.8259	0.0000	854.72
HS	TP_W	TP_M	J	0.8154	0.0000	798.62
HS	TP_E	TP_W	J	0.8225	0.0000	851.81
HS	TP_M	TP_W	J	0.8267	0.0000	859.54
TP_E	TP_M	HS	J	-0.0047	0.0000	-1.85
TP_E	TP_W	HS	J	-0.0387	0.0000	-18.28
TP_E	TP_W	TP_M	J	-0.0420	0.0000	-27.59
TP_E	TP_M	TP_W	J	0.0261	0.0000	17.57
TP_M	TP_W	HS	J	-0.0345	0.0000	-16.54
TP_M	TP_W	TP_E	J	-0.0680	0.0000	-43.03

Supplemental Table 6. Complex patterns of historical introgression between *Dendrobium catenatum* (TP_E, TP_M, and TP_W) and *D. huoshanense* (HS).

H1	H2	H3	H4	JK-*D*	V(JK-*D*)	*Z*
HS	TP_M	TP_E	GCSH	0.7559	0	1359.88
HS	TP_W	TP_E	GCSH	0.7411	0	1258.78
HS	TP_E	TP_M	GCSH	0.7588	0	1341.43
HS	TP_W	TP_M	GCSH	0.7499	0	1219.71
HS	TP_E	TP_W	GCSH	0.7510	0	1327.55
HS	TP_M	TP_W	GCSH	0.7565	0	1296.79
TP_E	TP_M	HS	GCSH	-0.0069	0.000001	-5.72
TP_E	TP_W	HS	GCSH	-0.0224	0.000001	-18.42
TP_E	TP_W	TP_M	GCSH	-0.0383	0.000001	-46.16
TP_E	TP_M	TP_W	GCSH	0.0256	0.000001	32.70
TP_M	TP_W	HS	GCSH	-0.0153	0.000002	-11.04
TP_M	TP_W	TP_E	GCSH	-0.0638	0.000001	-74.97

Supplemental Table 7. The posterior means and 95% HPD credible intervals (CIs) for parameters in BPP analyses of a dataset comprising coding loci on chromosome 1 from *D. huoshanense* (HS) and the eastern ecotype (TP_E) of *D. catenatum* were evaluated under two models with varying introgression directions.

**Coding loci (225 loci)**	**Model I (HS to TP_E)**				**Model O (TP_E to HS)**			
Param	Mean	S.D	2.5%HPD	97.5%HPD	Mean	S.D	2.5%HPD	97.5%HPD
theta:HS	0.02254	0.00909	0.00596	0.04017	0.03457	0.01065	0.01346	0.05459
theta:TP_E	0.04918	0.01526	0.01995	0.07813	0.05248	0.01299	0.02602	0.07666
theta:R	0.28505	0.01563	0.25459	0.31582	0.28523	0.01554	0.25540	0.31621
theta:S	0.10273	0.00801	0.08765	0.11875	0.08937	0.00590	0.07801	0.10101
theta:H	0.11523	0.00615	0.10342	0.12743	0.12871	0.00800	0.11342	0.14457
tau:R	0.04220	0.00276	0.03689	0.04771	0.04204	0.00270	0.03684	0.04741
tau:S	0.00084	0.00062	0.00006	0.00203	0.00128	0.00074	0.00012	0.00268
tau:H	0.00084	0.00062	0.00006	0.00203	0.00128	0.00074	0.00012	0.00268
phi:S	n/a	n/a	n/a	n/a	0.02065	0.00573	0.01057	0.03207
phi:H	0.01078	0.00352	0.00521	0.01764	n/a	n/a	n/a	n/a

These analyses used a dataset comprising 205 coding loci on chromosome 1, obtained from *D. huoshanense* (HS) and the eastern ecotype (TP_E) of *D. catenatum*. “n/a” means the parameter does not exist in the model.

Supplemental Table 8. The posterior means and 95% HPD credible intervals (CIs) for parameters in BPP analyses of a dataset comprising non-coding loci on chromosome 1 from *D. huoshanense* (HS) and the eastern ecotype (TP_E) of *D. catenatum* were evaluated under two models with varying introgression directions.

**Non-coding loci (225 loci)**	**Model I (HS to TP_E)**				**Model O (TP_E to HS)**			
Param	Mean	S.D	2.5%HPD	97.5%HPD	Mean	S.D	2.5%HPD	97.5%HPD
theta:HS	0.01687	0.00218	0.01277	0.02115	0.04327	0.00563	0.03218	0.05426
theta:TP_E	0.12712	0.01123	0.10592	0.14944	0.10973	0.01535	0.08246	0.14014
theta:R	0.15078	0.00966	0.13219	0.17003	0.16030	0.01008	0.14089	0.18034
theta:S	0.29333	0.04667	0.20537	0.38647	0.04494	0.00614	0.03339	0.05693
theta:H	0.02311	0.00303	0.01718	0.02907	0.04920	0.00742	0.03515	0.06240
tau:R	0.02067	0.00190	0.01696	0.02441	0.01925	0.00191	0.01537	0.02297
tau:S	0.00322	0.00052	0.00227	0.00423	0.00311	0.00155	0.00097	0.00591
tau:H	0.00322	0.00052	0.00227	0.00423	0.00311	0.00155	0.00097	0.00591
phi:S	n/a	n/a	n/a	n/a	0.02455	0.00751	0.01107	0.03951
phi:H	0.07577	0.01082	0.05564	0.09763	n/a	n/a	n/a	n/a

These analyses used a dataset comprising 225 non-coding loci on chromosome 1, obtained from *D. huoshanense* (HS) and the eastern ecotype (TP_E) of *D. catenatum*. “n/a” means the parameter does not exist in the model.

Supplemental Table 9. Bayes factors for comparing two introgression models for *D. huoshanense* (HS) and the eastern ecotype (TP_E) of *D. catenatum* datasets, calculated using thermodynamic integration with 16 Gaussian quadrature points.

Bij	Thermodynamic Integration
	16 points
Non-coding loci (*L*=225 loci)	
*B*IO	1.34E+13
Coding loci (*L*=205 loci)	
*B*IO	1.51E+04

The two models are Model I (HS to TP_E) and Model O (TP_E to HS). Bayes factor *Bij* indicates the evidence supporting model *i* over model *j*. We employ a 1% cutoff value: if *Bij* exceeds 100, it signifies strong support for model *i* and rejection of model *j*; if *Bij* is less than 0.01, it signifies strong support for model *j* and rejection of model *i*; values between 0.01 and 100 imply no strong preference for either model.

Supplemental Table 10. Mean values of molecular diversity indices in coding regions.

Type	Genomic Background				Introgressed Regions			
Group	HS	TP_E	TP_M	TP_W	HS	TP_E	TP_M	TP_W
N	37	60	25	34	37	60	25	34
Π	0.0968	0.0830	0.0741	0.0822	0.1435	0.0697	0.0505	0.0543
H_O	0.2071	0.1635	0.1855	0.1831	0.0678	0.1802	0.1876	0.1787
H_E	0.2001	0.1668	0.2121	0.1876	0.1632	0.1740	0.2110	0.1897
Ts/Tv	2.1205	2.0472	1.9295	2.1503	1.7500	1.7000	1.4966	1.5632
RTSS	0.3484	0.3589	0.2450	0.3179	0.6087	0.2663	0.1513	0.1806
RTVS	0.1643	0.1753	0.1270	0.1478	0.3478	0.1567	0.1011	0.1155
RPS	0.4951	0.5143	0.3610	0.4499	0.9214	0.4164	0.2469	0.2935
RPSS	0.1850	0.0403	0.0097	0.0330	0.6204	0.0209	0.0014	0.0199

Number of individuals (*N*), nucleotide diversity (π), observed heterozygosity (H_O), expected heterozygosity (H_E), Transition-Transversion ratio (Ts/Tv), ratio of transitions sites (RTSS), ratio of transversions sites (RTVS), ratio of polymorphic sites (RPS) and ratio private substitutions sites (RPSS) are listed in the genomic and introgressed coding regions. The western (TP_W), central (TP_M), and eastern ecotypes (TP_E) of *D. catenatum* and *D. huoshanense* (HS) are listed in the table.

Supplemental Table 11. Sample size and genetic diversity of *Dendrobium huoshanense* and *D. catenatum* populations.

Ts/Tv, Ratio of transition and transversion sites. RTSS, Ratio of transition sites. RTVS, Ratio of transversion sites. RPS, Ratio of polymorphic sites. RPSS, Ratio of private substitution sites.

Supplemental Table 12. Sample size and genetic diversity of *Dendrobium huoshanense* and *D. catenatum* populations in introgressed regions.

Ts/Tv, Ratio of transition and transversion sites. RTSS, Ratio of transition sites. RTVS, Ratio of transversion sites. RPS, Ratio of polymorphic sites. RPSS, Ratio of private substitution sites.

Supplemental Table 13. Mean values of molecular diversity indices.

	Genomic background	Introgressed regions
Sites/Kb	90.9805	102.8901
Pi $_{TP\_E}$	0.1062	0.0926
Pi $_{TP\_TREE}$	0.1075	0.1007
Pi $_{HS}$	0.1299	0.1016
D_{xy} $_{TP\_E\ vs\ TP\_TREE}$	0.1167	0.1022
D_{xy} $_{TP\_E\ vs\ HS}$	0.2505	0.1603
D_{xy} $_{TP\_TREE\ vs\ HS}$	0.2551	0.1729
F_{ST} $_{TP\_E\ vs\ TP\_TREE}$	0.0310	0.0202
F_{ST} $_{TP\_E\ vs\ HS}$	0.2945	0.2408
F_{ST} $_{TP\_TREE\ vs\ HS}$	0.2964	0.2283
Rho $_{TP\_E}$	509.9926	542.7045
Rho $_{TP\_W}$	291.3666	322.7760
Tajima‘s D $_{TP\_E}$	0.4308	0.4322
Tajima’s D $_{TP\_TREE}$	0.5518	0.5119

The western in arboreal habitat (TP_TREE) and eastern ecotypes (TP_E) of *Dendrobium catenatum* and *D. huoshanense* (HS) are listed in the table.

Supplemental Table 14. The Gene Ontology (GO) terms associated with introgressed genes.

GO ID	GO Term	GO category	Number of genes
GO:0005515	protein binding	MF	18
GO:0005524	ATP binding	MF	13
GO:0008270	zinc ion binding	MF	10
GO:0004672	protein kinase activity	MF	8
GO:0004713	protein tyrosine kinase activity	MF	8
GO:0003824	catalytic activity	MF	6
GO:0000166	nucleotide binding	MF	5
GO:0003723	RNA binding	MF	5
GO:0003700	DNA-binding transcription factor activity	MF	4
GO:0046872	metal ion binding	MF	4
GO:0004553	hydrolase activity, hydrolyzing O-glycosyl compounds	MF	3
GO:0003964	RNA-directed DNA polymerase activity	MF	3
GO:0003676	nucleic acid binding	MF	3
GO:0043565	sequence-specific DNA binding	MF	3
GO:0016627	oxidoreductase activity, acting on the CH-CH group of donors	MF	2
GO:0016491	oxidoreductase activity	MF	2
GO:0003777	microtubule motor activity	MF	2
GO:0016788	hydrolase activity, acting on ester bonds	MF	2
GO:0017111	nucleoside-triphosphatase activity	MF	2
GO:0004842	ubiquitin-protein transferase activity	MF	2
GO:0019829	ATPase-coupled cation transmembrane transporter activity	MF	2
GO:0008198	ferrous iron binding	MF	2
GO:0000287	magnesium ion binding	MF	2
GO:0008017	microtubule binding	MF	2
GO:0016616	oxidoreductase activity, acting on the CH-OH group of donors, NAD or NADP as acceptor	MF	1
GO:0016651	oxidoreductase activity, acting on NAD(P)H	MF	1
GO:0008569	ATP-dependent microtubule motor activity, minus-end-directed	MF	1
GO:0004523	RNA-DNA hybrid ribonuclease activity	MF	1
GO:0004185	serine-type carboxypeptidase activity	MF	1
GO:0004527	exonuclease activity	MF	1
GO:0004222	metalloendopeptidase activity	MF	1
GO:0004563	beta-N-acetylhexosaminidase activity	MF	1
GO:0016787	hydrolase activity	MF	1
GO:0004425	indole-3-glycerol-phosphate synthase activity	MF	1
GO:0003855	3-dehydroquinate dehydratase activity	MF	1
GO:0004618	phosphoglycerate kinase activity	MF	1

GO:0004617	phosphoglycerate dehydrogenase activity	MF	1
GO:0016887	ATPase activity	MF	1
GO:0003924	GTPase activity	MF	1
GO:0004325	ferrochelatase activity	MF	1
GO:0004764	shikimate 3-dehydrogenase (NADP+) activity	MF	1
GO:0016760	cellulose synthase (UDP-forming) activity	MF	1
GO:0003843	1,3-beta-D-glucan synthase activity	MF	1
GO:0008168	methyltransferase activity	MF	1
GO:0015205	nucleobase transmembrane transporter activity	MF	1
GO:0043682	copper transmembrane transporter activity, phosphorylative mechanism	MF	1
GO:0022857	transmembrane transporter activity	MF	1
GO:0051539	4 iron, 4 sulfur cluster binding	MF	1
GO:0016597	amino acid binding	MF	1
GO:0005509	calcium ion binding	MF	1
GO:0005525	GTP binding	MF	1
GO:0051287	NAD binding	MF	1
GO:0003735	structural constituent of ribosome	MF	1
GO:0046983	protein dimerization activity	MF	1
GO:0016021	integral component of membrane	CC	9
GO:0016020	membrane	CC	7
GO:0005871	kinesin complex	CC	2
GO:0005622	intracellular	CC	2
GO:0005669	transcription factor TFIID complex	CC	1
GO:0005852	eukaryotic translation initiation factor 3 complex	CC	1
GO:0000148	1,3-beta-D-glucan synthase complex	CC	1
GO:0016592	mediator complex	CC	1
GO:0005965	protein farnesyltransferase complex	CC	1
GO:0005737	cytoplasm	CC	1
GO:0005840	ribosome	CC	1
GO:0030117	membrane coat	CC	1
GO:0005856	cytoskeleton	CC	1
GO:0006468	protein phosphorylation	BP	8
GO:0008152	metabolic process	BP	6
GO:0055114	oxidation-reduction process	BP	5
GO:0006355	regulation of transcription, DNA-templated	BP	4
GO:0005975	carbohydrate metabolic process	BP	4
GO:0006075	(1 -> 3)-beta-D-glucan biosynthetic process	BP	4
GO:0016192	vesicle-mediated transport	BP	3
GO:0006278	RNA-dependent DNA biosynthetic process	BP	3
GO:0006886	intracellular protein transport	BP	3
GO:0015074	DNA integration	BP	3
GO:0007018	microtubule-based movement	BP	2
GO:0007165	signal transduction	BP	2

GO:0006725	cellular aromatic compound metabolic process	BP	2
GO:0006629	lipid metabolic process	BP	2
GO:0006812	cation transport	BP	2
GO:0006508	proteolysis	BP	2
GO:0031047	gene silencing by RNA	BP	1
GO:0006352	DNA-templated transcription, initiation	BP	1
GO:0006396	RNA processing	BP	1
GO:0045454	cell redox homeostasis	BP	1
GO:0042127	regulation of cell population proliferation	BP	1
GO:0030833	regulation of actin filament polymerization	BP	1
GO:0006357	regulation of transcription by RNA polymerase II	BP	1
GO:0006412	translation	BP	1
GO:0006351	transcription, DNA-templated	BP	1
GO:0044237	cellular metabolic process	BP	1
GO:0009186	deoxyribonucleoside diphosphate metabolic process	BP	1
GO:0009116	nucleoside metabolic process	BP	1
GO:0006505	GPI anchor metabolic process	BP	1
GO:0006096	glycolytic process	BP	1
GO:0006511	ubiquitin-dependent protein catabolic process	BP	1
GO:0030244	cellulose biosynthetic process	BP	1
GO:0006784	heme A biosynthetic process	BP	1
GO:0006783	heme biosynthetic process	BP	1
GO:0006564	L-serine biosynthetic process	BP	1
GO:0009058	biosynthetic process	BP	1
GO:0018343	protein farnesylation	BP	1
GO:0015851	nucleobase transport	BP	1
GO:0006825	copper ion transport	BP	1
GO:0015743	malate transport	BP	1
GO:0030001	metal ion transport	BP	1
GO:0015914	phospholipid transport	BP	1
GO:0015031	protein transport	BP	1
GO:0006904	vesicle docking involved in exocytosis	BP	1
GO:0055085	transmembrane transport	BP	1

Supplemental Table 15. KEGG pathways of introgressed genes.

MapID	MapTitle	*P* value	Gene number
dct01100	Metabolic pathways	0	46
dct01110	Biosynthesis of secondary metabolites	3.00E-18	35
dct04626	Plant-pathogen interaction	1.00E-132	33
dct04075	Plant hormone signal transduction	0	22
dct01230	Biosynthesis of amino acids	9.00E-161	19
dct03018	RNA degradation	1.00E-20	17
dct01200	Carbon metabolism	3.00E-10	14
dct00010	Glycolysis / Gluconeogenesis	0	10
dct03013	RNA transport	0	10
dct00130	Ubiquinone and other terpenoid-quinone biosynthesis	0	9
dct00030	Pentose phosphate pathway	0	9
dct00940	Phenylpropanoid biosynthesis	0	9
dct03015	mRNA surveillance pathway	9.00E-78	8
dct00710	Carbon fixation in photosynthetic organisms	5.00E-21	7
dct00052	Galactose metabolism	2.00E-146	6
dct00051	Fructose and mannose metabolism	3.00E-143	6
dct00520	Amino sugar and nucleotide sugar metabolism	0	6
dct00511	Other glycan degradation	2.00E-43	6
dct04016	MAPK signaling pathway - plant	6.00E-117	6
dct00604	Glycosphingolipid biosynthesis - ganglio series	4.00E-43	4
dct04120	Ubiquitin mediated proteolysis	0	4
dct00513	Various types of N-glycan biosynthesis	1.00E-86	4
dct00531	Glycosaminoglycan degradation	3.00E-09	4
dct00603	Glycosphingolipid biosynthesis - globo and isoglobo	2.00E-25	4
dct04141	Protein processing in endoplasmic reticulum	0	3
dct00220	Arginine biosynthesis	1.00E-07	3
dct00860	Porphyrin and chlorophyll metabolism	0	3
dct04144	Endocytosis	0	3
dct01210	2-Oxocarboxylic acid metabolism	6.00E-15	3
dct00965	Betalain biosynthesis	4.00E-09	3
dct00230	Purine metabolism	0	2
dct00240	Pyrimidine metabolism	3.00E-61	2
dct00400	Phenylalanine, tyrosine and tryptophan biosynthesis	3.00E-169	2
dct00440	Phosphonate and phosphinate metabolism	2.00E-177	2
dct00480	Glutathione metabolism	0	2
dct00564	Glycerophospholipid metabolism	0	2
dct00900	Terpenoid backbone biosynthesis	6.00E-22	2
dct01040	Biosynthesis of unsaturated fatty acids	0	2
dct01212	Fatty acid metabolism	0	2
dct00062	Fatty acid elongation	0	2
dct00190	Oxidative phosphorylation	1.00E-71	2

Supplemental Table 16. List of introgressed genes with putative functions related to the abiotic stress response.

ID	*P*_Value	Gene	Term
Dca001860	0	*NFS2*	Iron incorporation into metallo-sulfur cluster
Dca023803	3.00E-143	*HHP1*	Response to salt stress
Dca023805	5.00E-21	*HHP1*	Response to salt stress
Dca009082	0	*TKL-1*	Could act as a stress sensor involved in adaptation process
Dca002113	0	*ECR*	Wax biosynthetic process
Dca019076	3.00E-18	*AHK2*	Response to osmotic stress/ Response to salt stress
Dca004998	1.00E-71	*WRKY33*	Response to cold/ Response to osmotic stress/ Response to salt stress
Dca001590	0	*CSLD5*	Response to osmotic stress/response to salt stress

Supplemental Table 17. List of introgressed genes with putative functions related to organ development.

ID	*P*_Value	Gene	Term
Dca014947	3.00E-10	*CO*	Flower development/<br>Regulation of flower development
Dca022015	0	*FY*	Flower development
Dca016387	9.00E-161	*BRI1*	Pollen exine formation/<br>Leaf development/<br>Positive regulation of flower development/<br>Regulation of seedling development/<br>Response to UV-B
Dca016049	1.00E-132	*AP2*	Specification of floral organ identity/<br>Seed development/<br>Plant ovule development/<br>Flower development
Dca002626	1.00E-20	*CDC48A*	Pollen germination/<br>Pollen tube growth
Dca021033	0	*EMB3004*	Embryo development ending in seed dormancy
Dca001019	9.00E-78	*GAI*	Regulation of seed dormancy process/<br>Regulation of seed germination
Dca015004	2.00E-146	*IGPS*	Tryptophan biosynthetic process
Dca026733	2.00E-43	*ILL9*	Auxin metabolic process
Dca002377	0	*BIG*	Inflorescence morphogenesis/<br>Lateral root formation
Dca022816	4.00E-43	*MPK13*	Lateral root formation
Dca012576	0	*VCS*	Leaf morphogenesis
Dca001590	0	*CSLD5*	Involved in stem and root growth
Dca002623	1.00E-07	*NRPC2*	Required for the development of mature gametophytes

Supplemental Table 18. List of introgressed genes with putative functions related to DNA repair.

ID	*P*_Value	Gene	Term
Dca007887	0	*KIN5A*	Mitotic cytokinesis
Dca007593	1.00E-86	*MER3*	Reciprocal meiotic recombination
Dca024102	6.00E-117	*MER3*	Reciprocal meiotic recombination
Dca022609	3.00E-09	*MHF2*	Acts in the same pathway as FANCM to restrain class II meiotic crossing over (CO), and acts with FANCM during meiosis to repair interstrand cross-links (ICLs).
Dca028034	2.00E-25	*MHF2*	Acts in the same pathway as FANCM to restrain class II meiotic crossing over (CO), and acts with FANCM during meiosis to repair interstrand cross-links (ICLs).
Dca012584	0	*PA200*	Involved in DNA damage response
Dca014891	0	*TSO2*	DNA repair
Dca010921	0	*4CL1*	Phenylpropanoid metabolic process
Dca016387	9.00E-161	*BRI1*	Response to UV-B

Supplemental Table 19. List of introgressed genes with putative functions related to the defense response.

ID	*P*_Value	Gene	Term
Dca023963	0	*FLS2*	Defense response to bacterium
Dca012741	4.00E-09	*PBS1*	Defense response to bacterium
Dca004998	1.00E-71	*WRKY33*	Defense response to bacterium

Supplemental Table 20. Significantly enriched Gene Ontology (GO) terms for biological processes associated with the 255 genes under selection between the eastern ecotype of *Dendrobium catenatum* (TP_E) and *D. huoshanense* (HS) populations, identified using θ_π ratios and $Z(F_{ST})$ metrics.

Supplemental Table 21. Significantly enriched KEGG pathways for the 255 selected genes differentiating the eastern ecotype of *Dendrobium catenatum* (TP_E) and *D. huoshanense* (HS) populations, identified using θ_π ratios and $Z(F_{ST})$ metrics.

Supplemental Table 22. Significantly enriched Gene Ontology (GO) terms for biological processes associated with the 299 genes under selection between the eastern (TP_E) and western (TP_TREE) ecotypes of *Dendrobium catenatum* populations, identified using θ_π ratios and $Z(F_{ST})$ metrics.

Supplemental Table 23. Significantly enriched KEGG pathways for the 299 selected genes differentiating the eastern (TP_E) and western (TP_TREE) ecotypes of *Dendrobium catenatum* populations, identified using θ_{π} ratios and $Z(F_{ST})$ metrics.

Supplemental Table 24. Regional SNP distribution in 15 RAD-seq samples.

Supplemental Table 25. Regional variation counts across 15 individual resequencing datasets.

Supplemental Table 26. Molecular diversity indices for genomic background and coding regions.

Type	Genomic_background					Genomic_coding_regions				
**Group**	HS_JXLHS	TP_JXLHS	TP_HBXN	TP_YNGN	F1_Hybrids	HS_JXLHS	TP_JXLHS	TP_HBXN	TP_YNGN	F1_Hybrids
N	10	8	9	8	4	10	8	9	8	4
**Π**	0.1018	0.2676	0.2814	0.3615	0.3084	0.0664	0.2733	0.2938	0.3581	0.3235
H_O	0.5502	0.5479	0.5218	0.5733	0.6366	0.5399	0.5100	0.5025	0.5549	0.6294
H_E	0.3642	0.3704	0.3538	0.3877	0.4519	0.3572	0.3566	0.3502	0.3824	0.4484
**Ts/Tv**	2.5211	1.5801	1.5747	1.6040	1.5983	2.2259	1.6015	1.6165	1.6722	1.5940
**RTSS**	0.2061	0.4542	0.5005	0.5904	0.4333	0.1315	0.4819	0.5306	0.5995	0.4543
**RTVS**	0.0818	0.2874	0.3178	0.3681	0.2711	0.0591	0.3009	0.3282	0.3585	0.2850
**RPS**	0.2794	0.7224	0.7955	0.9325	0.6824	0.1857	0.7666	0.8389	0.9364	0.7215
**RPSS**	0.0007	0.0018	0.0029	0.0284	0.0001	0.0002	0.0012	0.0023	0.0175	0.0001

The number of individuals (N), nucleotide diversity (π), observed heterozygosity (H_O), expected heterozygosity (H_E), transition-to-transversion ratio (T_S/T_V), ratio of transition sites (RTSS), ratio of transversion sites (RTVS), ratio of polymorphic sites (RPS), and ratio of private substitutions sites (RPSS) are presented for the genomic coding regions. The western (TP_W), central (TP_M), and eastern (TP_E) ecotypes of *Dendrobium catenatum* and *D. huoshanense* (HS) are included in the table.

Supplemental Table 27. Pairwise F_{ST} values between *Dendrobium huoshanense* and three *D. catenatum* populations.

	**HS_JXLHS**	**TP_HBXN**	**TP_JXLHS**	**TP_YNGN**
**HS_JXLHS**	0	0.2485	0.2700	0.2944
**TP_HBXN**	0.2460	0	0.0493	0.0514
**TP_JXLHS**	0.2605	0.0382	0	0.0679
**TP_YNGN**	0.2828	0.0380	0.0539	0

D. huoshanense from Longhushan Mountain, Jiangxi Province (HS_JXLHS); *D. catenatum* from Longhushan Mountain, Jiangxi Province (TP_JXLHS); *D. catenatum* from Xianning County, Hubei Province (TP_HBXN); and *D. catenatum* from Guangnan County, Yunnan Province (TP_YNGN). The upper triangle displays F_{ST} values based on genomic coding regions, while the lower triangle presents F_{ST} values based on the genomic background.

Supplemental Table 28. Comparative analysis of f_3 statistics, standard errors (SE), and *Z*-scores between *Dendrobium huoshanense* (HS) and three geographic populations of *D. catenatum* (TP).

A	B	C	f_3(A; B, C)	Standard error	Z-score
HS_JXLHS	TP_HBXN	TP_JXLHS	0.0412	0.0050	8.2058
TP_HBXN	TP_JXLHS	HS_JXLHS	0.0039	0.0005	7.9885
TP_JXLHS	TP_HBXN	HS_JXLHS	-0.0011	0.0008	-1.4118
HS_JXLHS	TP_HBXN	TP_YNGN	0.0469	0.0051	9.1170
TP_HBXN	TP_YNGN	HS_JXLHS	-0.0019	0.0006	-3.1424
TP_YNGN	TP_HBXN	HS_JXLHS	0.0060	0.0017	3.5805
HS_JXLHS	TP_JXLHS	TP_YNGN	0.0425	0.0048	8.9391
TP_JXLHS	TP_YNGN	HS_JXLHS	-0.0024	0.0008	-3.2108
TP_YNGN	TP_JXLHS	HS_JXLHS	0.0104	0.0015	6.9982

D. huoshanense from Longhushan Mountain, Jiangxi Province (HS_JXLHS); *D. catenatum* from Longhushan Mountain, Jiangxi Province (TP_JXLHS); *D. catenatum* from Xianning County, Hubei Province (TP_HBXN); and *D. catenatum* from Guangnan County, Yunnan Province (TP_YNGN).
